# The mechanistic study of codonopsis pilosula on laryngeal squamous cell carcinoma based on network pharmacology and experimental validation

**DOI:** 10.3389/fphar.2025.1542116

**Published:** 2025-04-25

**Authors:** Huina Guo, Yichen Lou, Xiaofang Hou, Xiaoya Guan, Yujia Guo, Qi Han, Xuting Xue, Ying Wang, Long He, Zhongxun Li, Chunming Zhang

**Affiliations:** ^1^ Shanxi Key Laboratory of Otorhinolaryngology Head and Neck Cancer, First Hospital of Shanxi Medical University, Taiyuan, China; ^2^ Shanxi Province Clinical Medical Research Center for Precision Medicine of Head and Neck Cancer, First Hospital of Shanxi Medical University, Taiyuan, China; ^3^ The First Clinical Medical College of Shanxi Medical University, Taiyuan, China; ^4^ Department of Otolaryngology Head and Neck Surgery, First Hospital of Shanxi Medical University, Taiyuan, China

**Keywords:** codonopsis pilosula, laryngeal squamous cell carcinoma, MAPK3, network pharmacology, experimenntal validation

## Abstract

**Introduction:**

Laryngeal squamous cell carcinoma (LSCC) is a common malignant tumor of the head and neck, with poor prognosis for advanced patients, and there is an urgent need to find new treatment strategies. Codonopsis pilosula, a traditional Chinese medicinal herb, possesses various pharmacological activities, but its antitumor effects and mechanisms in LSCC are still unclear. The aim of this study was to systematically investigate the potential antitumor mechanism of Codonopsis pilosula in LSCC.

**Methods:**

In this study, we screened the effective compounds and targets of Codonopsis pilosula by TCMSP, ETCM and BATMAN-TCM databases, and screened targets related to LSCC by combining DisGeNET, GeneCards database and Cytoscape software. KEGG pathway enrichment analysis was utilized to explore the related signaling pathways. The core targets were further screened based on TCGA and GEO database analysis, and molecular docking was carried out to predict their binding ability to effective compounds. The presence of key compounds was verified by LC-MS, the MAPK3 expression was detected by qPCR in LSCC tissues, and the effects of MAPK3 knockdown on proliferation, migration, invasion, cell cycle, and apoptosis of LSCC cells were evaluated by cellular function assays.

**Results:**

In this study, 22 targets of Codonopsis pilosula that might regulate LSCC were screened based on network pharmacology. KEGG pathway enrichment analysis showed that Codonopsis pilosula-LSCC targets were mainly involved in HIF-1, TNF, IL-17 and FoxO signaling pathways. Based on TCGA and GEO database analysis, MAPK3 was identified as the core target of Codonopsis pilosula-LSCC. The molecular docking results showed that a variety of effective compounds from Codonopsis pilosula had strong binding abilities to MAPK3, among them, Caprylic Acid, Emodin and Luteolin have been confirmed by LC-MS. QPCR analysis indicated that MAPK3 was highly expressed in LSCC tissues. MAPK3 knockdown significantly inhibits LSCC cell proliferation, migration and invasion. It also suppresses LSCC cell growth by blocking the cell cycle and inducing apoptosis.

**Conclusion:**

Codonopsis pilosula exerts antitumor effects in LSCC through the regulation of MAPK3 and multiple signaling pathways, providing a theoretical basis for its clinical application.

## 1 Introduction

Laryngeal squamous cell carcinoma (LSCC) is one of the most common malignant tumors of the head and neck (Zhao et al., 2023; Guo et al., 2024a; Sun et al., 2024), with a large number of new cases occurring globally every year, especially in northern China. In the early stages of the disease, LSCC lacks obvious clinical symptoms, and the anatomical location of the larynx further increases the difficulty of early detection, resulting in the majority of patients being in the advanced stage of LSCC at the time of diagnosis (Wang Z. et al., 2022; Liberale et al., 2023). Despite the continuous improvement of various treatments, including surgery, radiotherapy, and chemotherapy, the overall prognosis of LSCC is still unsatisfactory and the mortality rate remains high (Wang J. et al., 2022). Studies have shown that smoking, alcohol consumption, and air pollution are risk factors for LSCC pathogenesis (Zheng et al., 2023), and many molecular markers for early diagnosis, treatment, prediction, and prognosis have also been screened (Yang et al., 2023b; Zhao et al., 2024; Zhu et al., 2024), but unfortunately, there is still a lack of satisfactory therapeutic strategies for LSCC. In addition, existing treatments are usually accompanied by severe functional sequelae, such as vocal cord damage and dysphagia (Li et al., 2024), which greatly affects patients’ quality of life and demonstrates the limitations of current therapies. Therefore, the urgent need for novel therapeutic approaches is emphasized.

**FIGURE 1:**
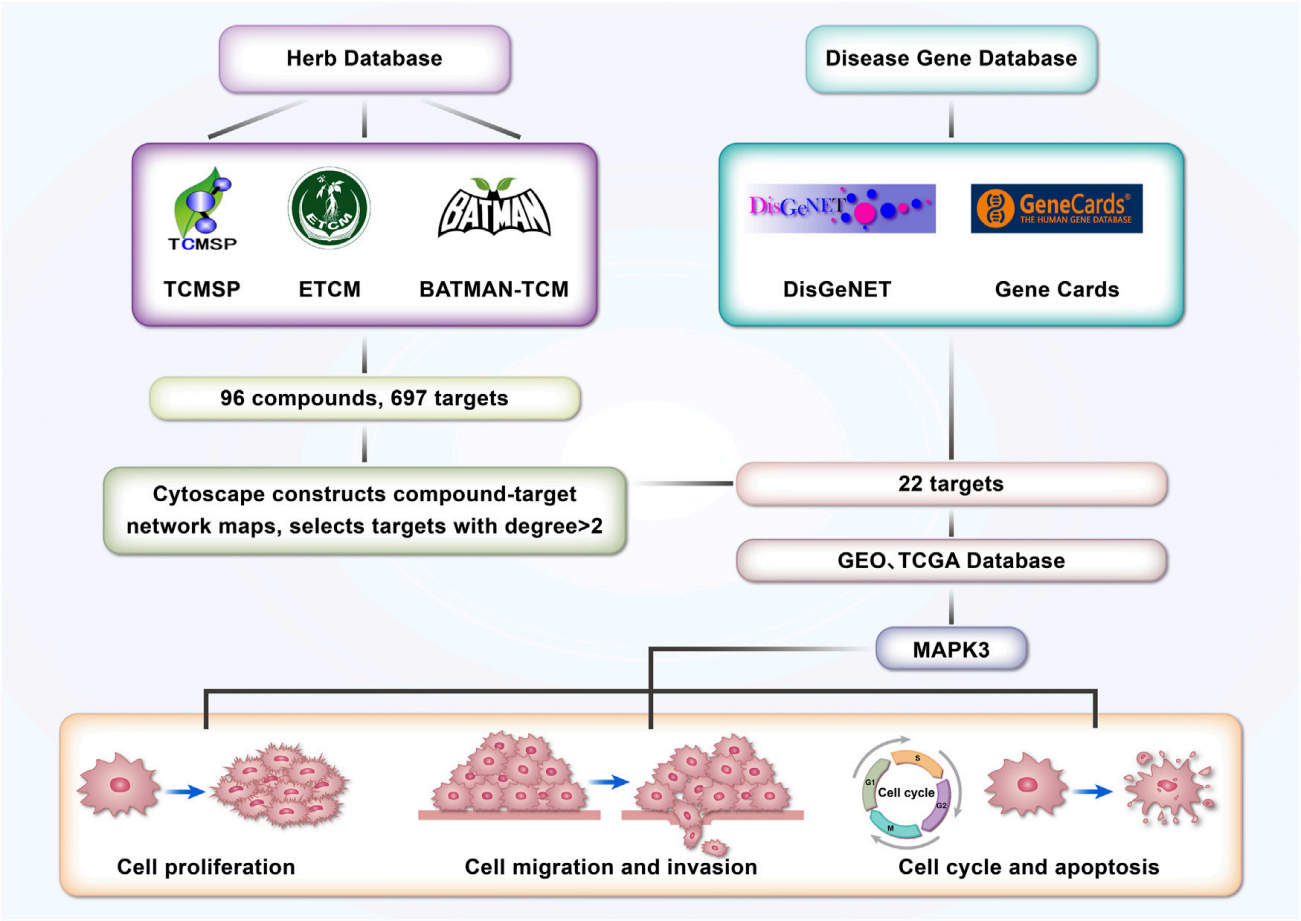
Article strategy and flowchart.

Codonopsis pilosula, a Chinese herb with a long history (Dong et al., 2023), has gradually received extensive attention from the medical community (Guo et al., 2024b) because of its various pharmacological effects such as antioxidant, anticancer, and anti-inflammatory (Tian and Tang, 2022). Recent studies have shown that Codonopsis pilosula and its active ingredients play critical roles in a variety of diseases (Guo et al., 2024b), such as the cardiovascular system (Lee et al., 2019; Shin et al., 2019), nervous system (Xie et al., 2024), digestive system (He et al., 2022), and immune system (Choi et al., 2023). However, whether Codonopsis pilosula has an effect on LSCC has not been fully investigated. In this study, 22 target genes were screened from LSCC through a network pharmacology approach combined with a database of LSCC-related disease genes. Further analysis using TCGA and GEO databases showed that MAPK3 plays a significant role in the treatment of LSCC with Codonopsis pilosula. This study provides a valuable theoretical basis for the molecular mechanism of Codonopsis pilosula in LSCC treatment and its potential clinical application. Figure 1 shows the research idea.

## 2 Materials and methods

### 2.1 Screening of active ingredients and targets of codonopsis pilosula

In this study, we screened the active ingredients and their targets of Codonopsis pilosula from TCMSP, ETCM and BATMAN-TCM databases. These databases contain abundant information on Chinese medicine compounds and their related targets, which provide reliable data support for the pharmacological study of Codonopsis pilosula and ensure the scientific basis for the investigation of the mechanism of action of Codonopsis pilosula in this study.

### 2.2 Acquisition of LSCC related disease genes

The DisGeNT database (https://www.disgenet.org/) and GeneCards database (https://www.genecards.org/) were utilized to obtain disease gene data associated with LSCC. Screening of these two databases identified disease genes associated with LSCC and provided the basis for network pharmacology analysis.

### 2.3 Compound-target network construction and core target screening of codonopsis pilosula

In this study, cytoscape 3.6.0 software was used to construct the compound-target interaction network diagram of Codonopsis pilosula. Subsequently, the Draw Venn Diagram (http://bioinformatics.psb.ugent.be/webtools/Venn/) tool was utilized to generate a Venn diagram of Codonopsis pilosula targets and LSCC disease genes to identify the intersecting genes between them. By screening these common genes, the core key targets were further screened using the String website and cytoscape software.

### 2.4 Molecular docking

Molecular docking is the prediction of binding affinity by modeling the interaction between ligands and receptor molecules. Using PubChem, we obtained the 2D structure of Codonopsis pilosula compounds and the protein receptor of MAPK3 from RCSB PDB. Autodock Vina molecular docking experiments were performed on the SailVina platform. These experiments were performed to simulate the binding process of Codonopsis pilosula compounds and MAPK3. At the same time, the pdb file and pdbqt file were converted into formats with OpenBabel software. The results of molecular docking between Codonopsis pilosula compounds and MAPK3 were finally visualized by Pymol software.

### 2.5 High performance liquid chromatography-mass spectrometry (LC-MS)

In this study, LC-MS was used to analyze the composition of Codonopsis pilosula extract. The Codonopsis pilosula extracts used in the study were purchased from Selleck, United States (Product No. E3141). The analysis was performed by high performance liquid chromatography (HPLC) coupled with mass spectrometry (MS) utilizing a Waters HSS T3 (100 × 2.1 mm, 1.8 μm) column equipped with an electrospray ionization (ESI) source. The samples were stored in an autosampler at 4°C throughout the analysis and were injected and analyzed according to optimized mobile phase gradient conditions. The resulting raw data were pre-processed by Progenesis QI (Waters Corporation, Milford, United States) software for baseline correction, peak identification, peak matching, retention time correction and peak alignment to generate a data matrix containing retention time, mass-to-charge ratio (m/z) and peak intensity. Subsequently, peaks containing secondary mass spectrometry (MS2) data were identified using Sanshu Biotech’s own secondary mass spectrometry database of herbal medicines and corresponding cleavage patterns. The match of MS2 was mainly evaluated by the secondary fragmentation score, which has a full score of 1. Higher scores indicate more reliable identification results. When the Score exceeds 0.5, the identification results are considered hign-confident. This method can effectively identify the main chemical components in Codonopsis pilosula extracts and provide data support for active ingredient analysis.

### 2.6 Tissue samples

The LSCC and their corresponding paracancerous tissue samples used in this study were obtained from surgical patients in the Department of Otolaryngology Head and Neck Surgery, the First Hospital of Shanxi Medical University. A total of 14 LSCC and adjacent normal tissue (ANM) samples were collected in this study, and none of the patients had received radiotherapy or chemotherapy interventions before surgery. The samples were assayed for MAPK3 expression levels by qPCR. To ensure ethical compliance, this study was approved by the Ethics Committee of the First Hospital of Shanxi Medical University (Ethics Approval No. KYLL-2025-017). Supplementary Table S1 details the clinical characteristics of LSCC patients.

### 2.7 Cell culture

In this study, LSCC cell line FD-LSC-1 (kindly provided by Professor Liang Zhou) (Gao et al., 2020; He et al., 2025) was cultured in BEGM medium containing 10% fetal bovine serum (FBS), and TU-686 cell line (YS2283, YaJi Biological, China) was grown in 1,640 medium containing 10% FBS. The cells were all cultured in a constant temperature incubator at 37°C with 5% CO_2_. Cell lines were regularly tested for *mycoplasma* using the *Mycoplasma* Detection Kit (FM321, TransGen, China). The TU-686 cell line was identified by STR and its cell lineage information was consistent with the ATCC database description. The FD-LSC-1 cell line was not characterized by STR, but to ensure experiments reliability, we regularly performed *mycoplasma* testing. When the cell density reached about 70%, the cells were transfected according to the instructions on the transfection reagents Lipofectamine 3,000 (L3000015, Thermo, United States).

### 2.8 siRNA synthesis

Two siRNA sequences targeting MAPK3 isoform1 (NM_002746.3) were used in this study: si-MAPK3-1: sence: 5′- GCU​ACU​UCC​UCU​ACC​AGA​UTT -3′, antisence: 5′- AUC​UGG​UAG​AGG​AAG​UAG​CTT-3'; si-MAPK3-2: sence: 5′- GAC​CGG​AUG​UUA​ACC​UUU​ATT -3′, antisence: 5'-UAA​AGG​UUA​ACA​UCC​GGU​CTT -3′, and negative control (NC) were synthesized with GenePharma (Shanghai, China). These siRNAs were used to transfect LSCC cell to silence the MAPK3 gene.

### 2.9 CCK8 assay

The CCK8 assay detects cell proliferation. The CCK8 kit used in this study was purchased from Yeasen (40203ES88, Shanghai, China). During the experiment, 3 × 10^3^ FD-LSC-1 cells and 2.5 × 10^3^ TU-686 cells were first inoculated in each well of the 96-well cell plate. After the cells were attached to the wall, 10 µL of CCK8 solution was added to each well according to the time points (0, 24, 48, 72, 96, 120 h) and incubated at 37°C for 1 h. Finally, the absorbance value of each well was measured at 450 nm with SpectraMax i3x Multi-Mode Microplate Reader (Molecular Devices, United States).

### 2.10 RTCA

In this study, the RTCA instrument (ACEA, Unites States) was used to detect cell index changes. TU-686 and FD-LSC-1 cells grown in the logarithmic phase were collected and counted. 50 μL of complete medium was taken to moisten the E-plate (300600890, ACEA, United States) and a blank control was run on the RTCA system to ensure background signal stabilization. 100 μL cell suspension containing 3 × 10^3^ FD-LSC-1 or 2.5 × 10^3^ TU-686 cells was spread evenly in each well of the E-plate, and allowed to stand at room temperature for 30 min. After standing, the E-plate was placed into the RTCA instrument for real-time monitoring. Start the RTCA software and set the sample collection every 4 h for a total of 24 times. This will enable you to obtain the cell index of the cells at different time points.

### 2.11 Transwell assay

Transwell assay was used to assess the migration and invasion ability of the cells in this experiment. Cells were suspended in serum-free medium and added to the upper chamber of the transwell (353,097, FALCON, United States). In the migration assay, no matrix gel was added to the upper chamber. In the invasion assay, the upper chamber was pre-coated with matrix gel. Next, complete medium containing serum was added to the lower chamber of the transwell and placed in an incubator at 37°C, 5% CO_2_ for 48 h. This was for cell migration or invasion across the chamber pore membrane. Subsequently, the cells in the upper chamber were gently wiped out, fixed in 4% paraformaldehyde, and stained with crystal violet. The number of cells crossing the pore membrane was counted microscopically to detect cell migration or invasion ability.

### 2.12 Cell cycle

In this study, the Cell Cycle and Apoptosis Analysis Kit (40301ES60, Yeasen Biotechnology, China) was used to analyze the cell cycle. Cells were selected in the logarithmic phase, trypsin digested and centrifuged to collect 2 × 10^5^ cells. Cells were washed with pre-cooled PBS and fixed in 70% pre-cooled ethanol at 4°C for 2 h. Cells were washed twice with pre-cooled PBS. To each 500 µL Staining Solution, 10 µL PI Solution and 10 µL RNase A Solution were added and mixed thoroughly to ensure a homogeneous staining solution. Add 500 µL of the configured PI Staining Solution to each sample, gently mix to make cell suspension, incubate at 37°C, avoiding light for 30 min, and then detect by flow cytometry (ACEA, Unites States).

### 2.13 Apoptosis

In this study, apoptosis was detected using Annexin V-APC/7-ADD Apoptosis Detection Kit (KGA1106, KeyGEN Biotech, China). Cells in the logarithmic growth phase were selected, cells were digested with EDTA-free trypsin, and 2 × 10^5^ cells were collected by centrifugation and washed twice in PBS. 500 μL of binding buffer was added, and the cells were gently blown to form a cell suspension. 5 μL Annexin V-APC and 5 µL 7-ADD stain were added to each sample, and the reaction was carried out at room temperature, darkly, reaction for 10 min. Finally, flow cytometry (ACEA, Unites States) was performed.

### 2.14 Data collection

We used several publicly available databases, including the TCGA database and the GEO database. The GEO database contains sequencing data GSE127165 (Wu et al., 2020), microarray databases GSE51985 (Lian et al., 2013), and GSE143224 (Nicolau-Neto et al., 2020). We processed and analyzed the data using Limma software in R to identify differences in LSCC and ANM genes.

### 2.15 Statistical analysis

This study was statistically analyzed with GraphPad Prism 9.0 software. Differences in data between two groups were compared using a two-tailed Student’s t-test, while differences in data between more than two groups were assessed via one-way ANOVA. The experiment results were expressed as mean ± standard deviation. The experiments were repeated three times for each group to ensure data reliability and consistency. Differential genes between the LSCC group and the ANM group in the GEO and TCGA databases were statistically analyzed with the Wilcoxon Rank Sum Test. Batch correlation analysis was performed using the Spearman method. P < 0.05 was considered statistically significant.

## 3 Results

### 3.1 Screening of effective components and targets of codonopsis pilosula

“Codonopsis pilosula” was entered into the TCMSP database. The effective compounds of Codonopsis pilosula were screened according to the criteria of oral bioavailability (OB) ≥20% and drug-likeness (DL) ≥0.10, and the potential targets of these compounds were predicted. All target gene names were converted to “GENE SYMBOL” format by the STRING database. A total of 32 effective compounds and 154 targets were discovered (Supplementary Table S2).

“Codonopsis pilosula” was entered into the, ETCM database. Based on the criteria of Druglikeness Weight of “moderate” and “good”, 13 active compounds and 211 targets were screened (Supplementary Table S3).

In the BATMAN-TCM database, “DANG SHEN” was entered, and 58 active compounds and 492 targets were obtained according to the screening condition of Confidence Score cutoff to 0.99 (LR = 1,626) (Supplementary Table S4).

**FIGURE 2:**
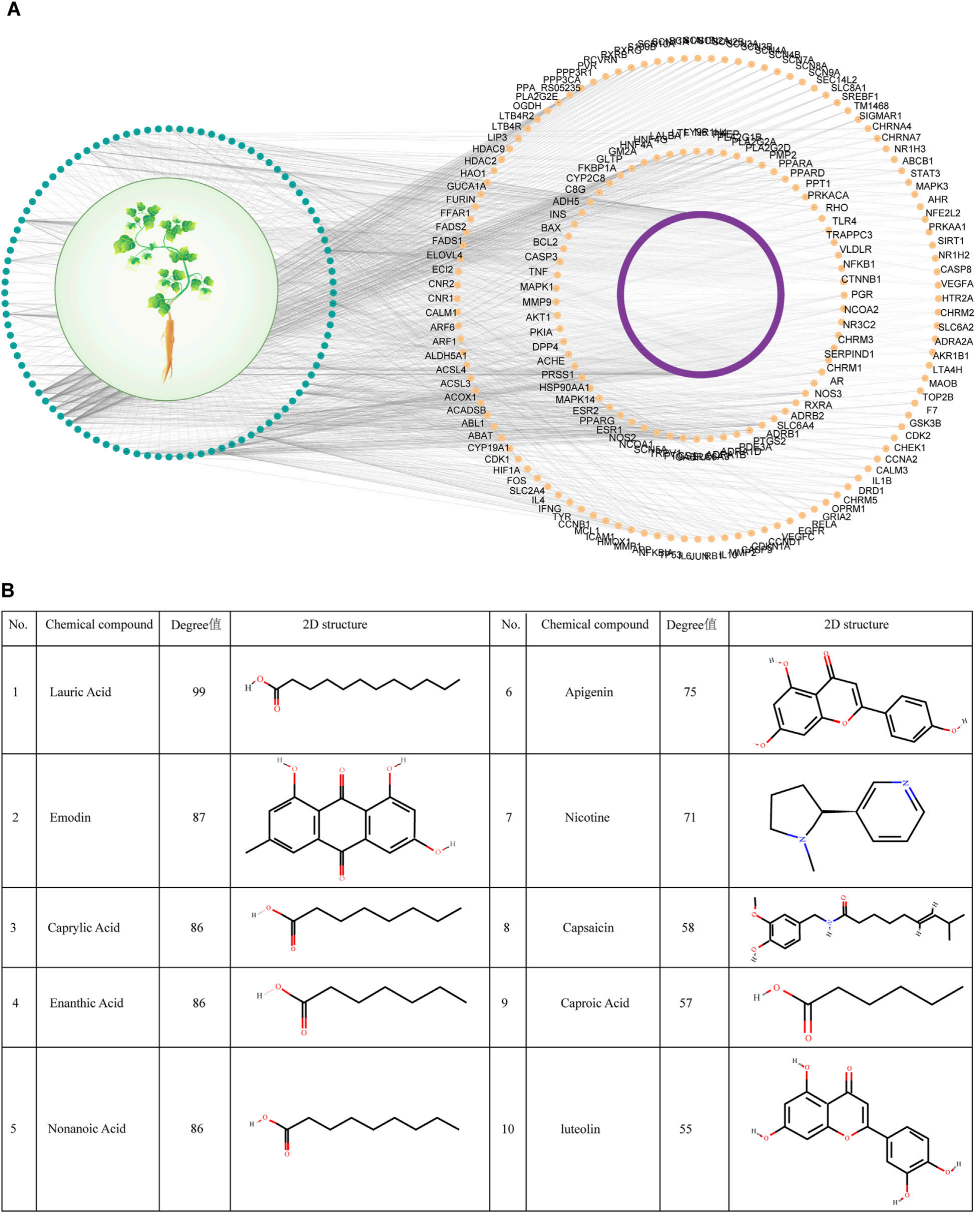
Screening of effective compounds and targets for Codonopsis pilosula. **(A)** Effective compound-target network diagram of Codonopsis pilosula. The green color represents Codonopsis pilosula compounds, the purple and yellow colors represent the corresponding targets of Codonopsis pilosula compounds, and the yellow color represents the 179 key targets of Codonopsis pilosula with a degree >2. **(B)** Effective compounds with the most targeted interactions in the Codonopsis pilosula effective compounds-targets network diagram.

The compounds and their targets for Codonopsis pilosula from the three databases were taken and pooled. 96 effective compounds and 697 targets were finally obtained. Cytoscape software was utilized to construct the interaction network diagram of effective compounds-targets of Codonopsis pilosula (Figure 2A). The topology analysis yielded an average degree value of 2.35 for all targets. Because the degree value is an integer, to ensure higher network centrality of the screened targets, targets with degree>2 were selected for further analysis in this study. Eventually, 179 key targets were screened (Figure 2A; Supplementary Table S5). According to the degree value magnitude, the effective compounds with the most targeted interactions in the network were obtained (Figure 2B).

### 3.2 Screening of key targets of codonopsis pilosula in LSCC

**FIGURE 3:**
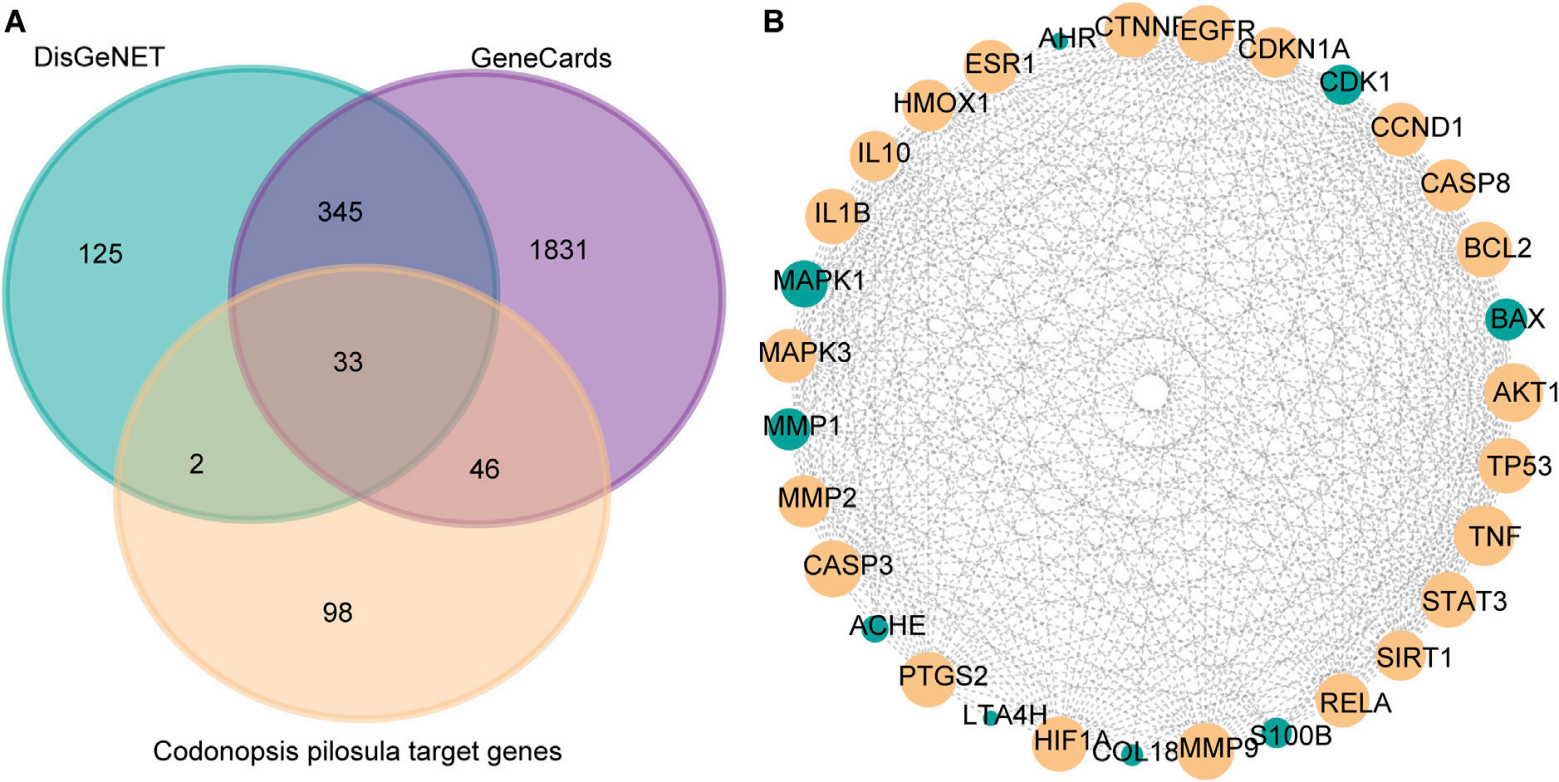
Screening of key targets for Codonopsis pilosula in LSCC. **(A)** Venn diagram showing the intersection of LSCC disease genes obtained from DisGeNET, GeneCards database with Codonopsis pilosula targets. 33 targets were obtained. **(B)** Based on Cytoscape software topology analysis, targets with degrees>21 were considered key targets. The yellow nodes represent 22 selected key targets.

DisGeNET is a comprehensive database covering information on diseases’ association with genes. It is widely used to study the relationship between genes and diseases. In this study, 504 genes associated with LSCC were screened by DisGeNET. Meanwhile, GeneCards, as a comprehensive human gene database, is specialized in diseases. GeneCards provides gene-disease association data, which can help researchers quickly identify genes associated with specific diseases. In this study, GeneCards was utilized to screen 2,254 genes correlated with LSCC. In order to screen potential molecular targets of Codonopsis pilosula in LSCC, this study intersected the LSCC disease genes obtained from DisGeNET and GeneCards with 179 Codonopsis pilosula targets identified by Cytoscape analysis. A total of 33 common targets were obtained using the Draw Venn Diagram tool (Figure 3A).

Next, we put 33 targets through the STRING website to construct a protein-protein interaction (PPI) network map. Among them, FADS1 and FADS2 did not have significant gene interactions with other genes, so they were removed from the analysis. Afterward, topology analysis was performed using cytoscape software, and 22 targets with degree values >21 were filtered out (Figure 3B).

### 3.3 KEGG pathway enrichment analysis of codonopsis pilosula

**FIGURE 4:**
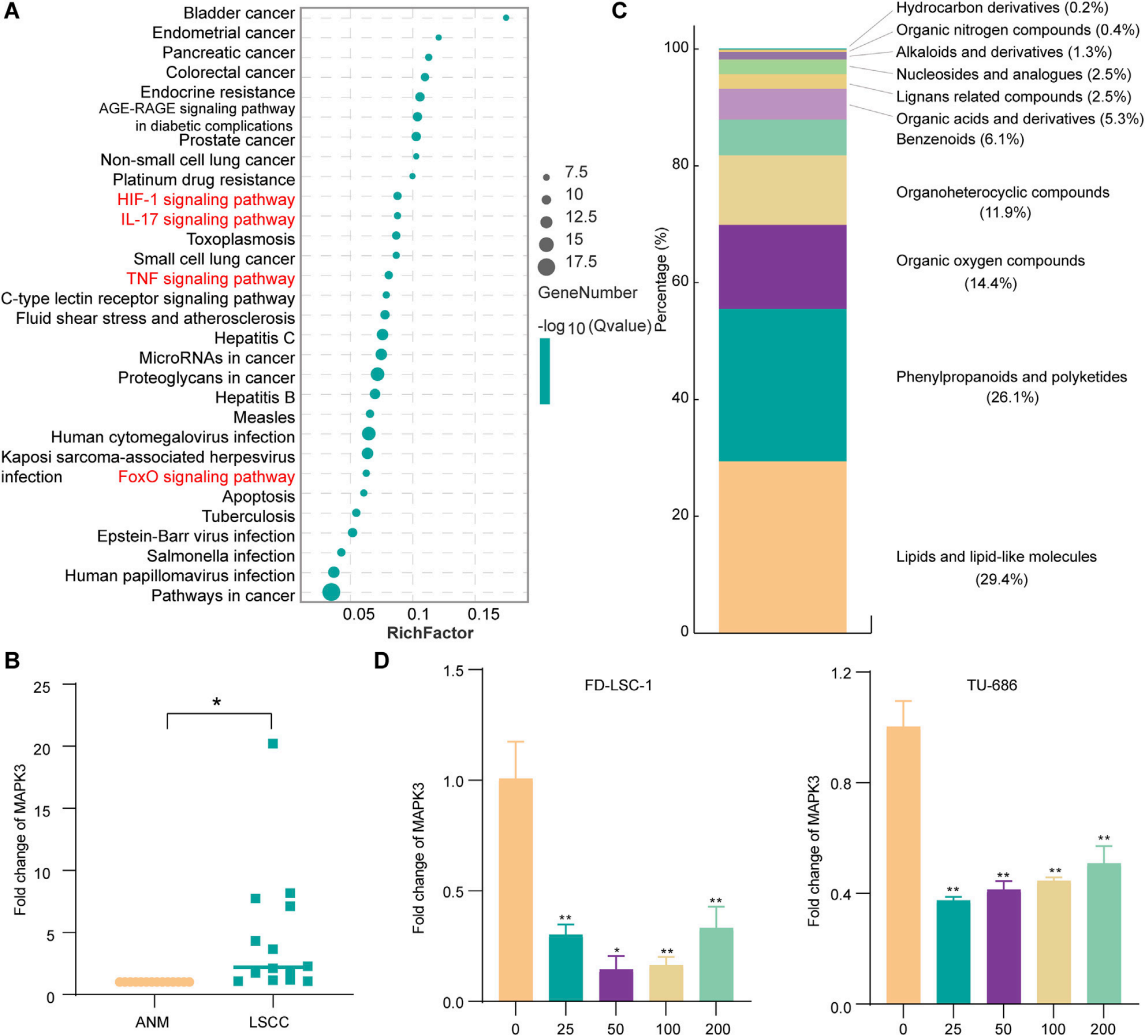
MAPK3 as a potential core target for Codonopsis pilosula action in LSCC. **(A)** The first 30 pathways were mapped according to the proportion of KEGG pathway-enriched genes and p-values. **(B)** MAPK3 expression was detected by qPCR assay in LSCC and ANM tissues. **(C)** Classification of compounds identified in Codonopsis pilosula extract by LC-MS analysis. **(D)** qPCR to detect MAPK3 expression after treatment with FD-LSC-1 and TU-686 at different concentrations of Codonopsis pilosula. Error bars represent the standard deviation (SD) of three independent experiments. *p < 0.05, **p < 0.01.

In order to further investigate the potential mechanism of action of the Codonopsis pilosula targets, this study utilized the STRING website to analyze the KEGG pathway enrichment of the 22 screened Codonopsis pilosula-LSCC targets. The results showed that 149 relevant pathways were found. Based on the ranking of false discovery rates, we selected the top 30 key pathways for in-depth analysis (Figure 4A). In addition, KEGG pathway analysis showed that 18 genes were associated with cancer pathways. This indicates that Codonopsis pilosula plays a crucial role in cancer development. The signaling pathways involved in Codonopsis pilosula-LSCC target include HIF-1 signaling pathway, TNF signaling pathway, IL-17 signaling pathway, and FoxO signaling pathway. These pathways play a crucial role in cancer cell proliferation, migration, invasion and immune regulation. They jointly affect tumorigenesis and development through multiple mechanisms.

### 3.4 MAPK3 as a potential key target of codonopsis pilosula acting on LSCC

To further investigate the role of Codonopsis pilosula targets in LSCC. In this study, we used bioinformatics to analyze the expression of 22 Codonopsis pilosula-LSCC targets in TCGA and GEO databases. The results showed that only MAPK3 exhibited significant differences in TCGA database and GEO database (GSE127165, GSE143224, GSE51985) (Supplementary Figures S1-S5). MAPK3, also known as ERK1, is an important signaling molecule in the cell. It mainly regulates biological processes, such as cell proliferation, differentiation, migration, etc. As a key component in the ERK1/2 signaling pathway, it plays a pro-cancer role in LSCC(Ma et al., 2022; Yang et al., 2023a) and head and neck squamous cell carcinoma (Luo et al., 2022; Savic et al., 2023; Xu et al., 2024). In addition, we analyzed MAPK3 expression in 14 pairs of LSCC and ANM tissues in this study. qPCR results showed that MAPK3 was highly expressed in LSCC tissues (Figure 4B). In addition, in this study, the composition of Codonopsis pilosula was analyzed using LC-MS to identify a variety of compounds, listed in Supplementary Table S6, and the identified compounds were classified using the ClassFire algorithm (Djoumbou Feunang et al., 2016). Codonopsis pilosula mainly contains phenylpropanoids and polyketides, lipids and lipid-like molecules, organic oxygen compounds and organoheterocyclic compounds and other categories (Figure 4C). Next, we treated LSCC cell lines FD-LSC-1 and TU-686 with different concentrations (0, 25, 50, 100, and 200 μg/mL) of Codonopsis pilosula extract for 24 h and analyzed MAPK3 expression by qPCR. The results showed that MAPK3 expression was significantly downregulated in LSCC cells under different Codonopsis pilosula concentrations (Figure 4D). Therefore, MAPK3 may be an effective target for Codonopsis pilosula in LSCC.

### 3.5 Molecular docking of codonopsis pilosula compounds with MAPK3

**FIGURE 5:**
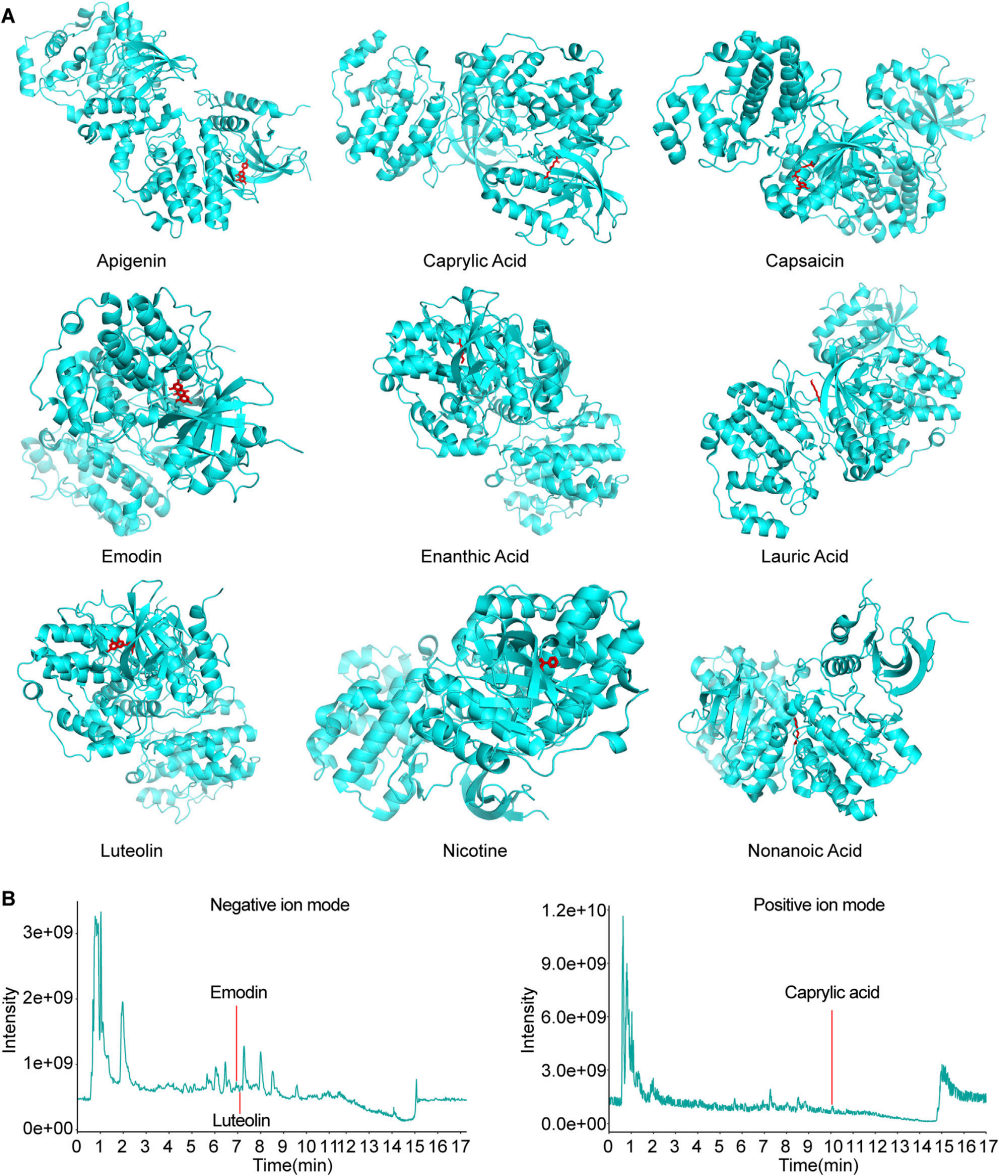
Molecular docking and LC-MS total ion chart (TIC) map analysis of effective compounds of Codonopsis pilosula with MAPK3. **(A)** Molecular docking of effective compounds of Codonopsis pilosula with MAPK3. **(B)** The compounds TIC of Codonopsis pilosula were identified by LC-MS. Different compounds were detected in positive and negative ion modes, and the chromatographic peaks of the active compounds of interest in the corresponding modes were labeled.

Cytoscape software was utilized to construct the effective compound-target network map of Codonopsis pilosula, from which the 10 effective compounds with the most targeted interactions were obtained: Apigenin, Caproic Acid, Caprylic Acid, Capsaicin, Emodin, Enanthic Acid, Lauric Acid, Luteolin, Nicotine, and Nonanoic Acid, and used these compounds as ligands for subsequent studies. Next, we performed molecular docking analysis of these ligands and the receptor MAPK3 using AutoDock Vina. The results showed that except for Caproic Acid, the remaining nine ligands have multiple potential binding sites to the receptor MAPK3. Specifically, Apigenin, Caprylic Acid, Capsaicin, Emodin, Enanthic Acid, Lauric Acid, Luteolin, Nicotine, and Nonanoic Acid had a free energy of binding to the receptor MAPK3 of −9.6 kcal/mol, −6.4 kcal/mol, −9.6 kcal/mol, −10.8 kcal/mol, −5.6 kcal/mol, −7.0 kcal/mol, −10.4 kcal/mol, −6.9 kcal/mol, −6.6 kcal/mol (Figure 5A). Thus, the molecular docking results revealed the key interactions between Codonopsis pilosula effective compounds and the receptor MAPK3. This provided an important theoretical basis for experimental validation.

Further combined with LC-MS analysis, only Caprylic Acid, Emodin and Luteolin were successfully detected among the above 10 compounds (Figure 5B). The other compounds were not detected, probably because their contents in Codonopsis pilosula were lower than the detection limit of LC-MS. They were affected by factors such as Codonopsis pilosula batch, origin difference, extraction process and assay method. This result suggests that although network pharmacological predictions can be used to screen for potentially active compounds, experimental validation still needs to take into account the distribution of compound contents in actual samples as well as the limitations of detection techniques. The results further support the potential interaction of specific compounds in Codonopsis pilosula extract with MAPK3. They provide an important basis for subsequent experimental studies.

### 3.6 Knockdown MAPK3 inhibits proliferation, migration and invasion of LSCC cells

**FIGURE 6:**
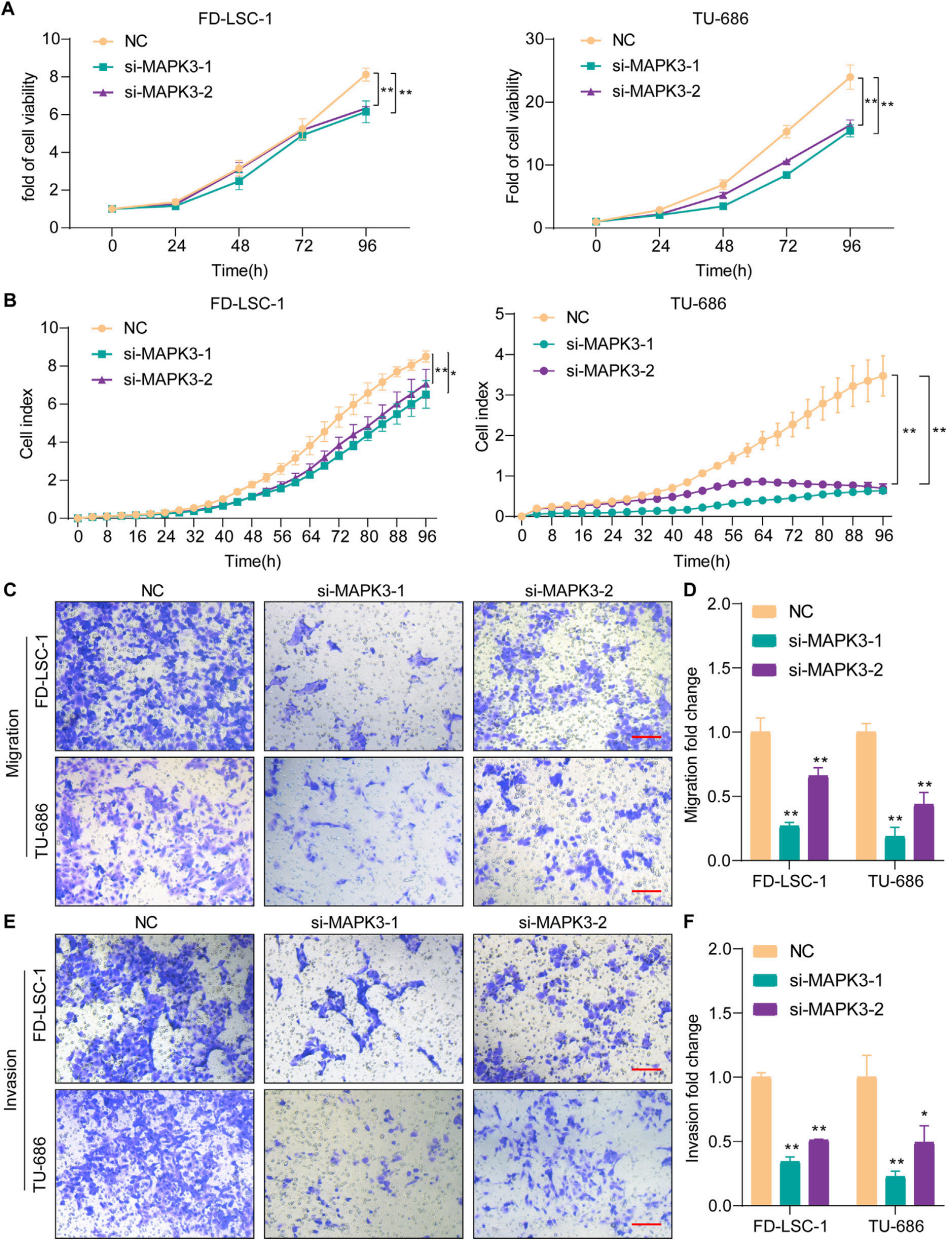
Knockdown MAPK3 inhibits LSCC cell proliferation, migration and invasion. **(A, B)** Cell proliferation was determined by CCK8 **(A)** and RTCA**(B)** after MAPK3 knockdown in FD-LSC-1 and TU-686 cells. **(C–F)** Migration **(C, D)** and invasion **(E, F)** were examined by transwell assays after MAPK3 silencing in FD-LSC-1 and TU-686 cells. Scale bar, 50 μm. Error bars represent the SD of three independent experiments. *p < 0.05, **p < 0.01.

To further investigate MAPK3 function in LSCC. We assessed si-MAPK3’s effect on cell viability by CCK8 and RTCA assays. The LSCC cell lines FD-LSC-1 and TU686 cells were transfected with si-MAPK3 or negative control NC. The knockdown efficiency was demonstrated by qPCR (Supplementary Figure S6). The results of the CCK8 assay showed that the cell viability of the si-MAPK3 group was significantly reduced compared to the control group (Figure 6A). Meanwhile, the RTCA assay to monitor cell index changes in real time showed that the cell index was significantly decreased in the si-MAPK3 group (Figure 6B). Together, these results indicate that MAPK3 plays a key role in maintaining cell viability. The proliferation ability of LSCC cells was considerably reduced after MAPK3 knockdown.

To further verify the effect of MAPK3 on LSCC, we detected its effect on LSCC cells by Transwell assay. The results showed that the migration (Figures 6C, D) and invasion (Figures 6E, F) abilities of FD-LSC-1 and TU-686 cells were significantly reduced after knockdown of MAPK3 with significant differences. The above results suggest that MAPK3 plays a crucial role in regulating LSCC cell migration and invasion.

### 3.7 Effect of MAPK3 on LSCC cell cycle and apoptosis

**FIGURE 7:**
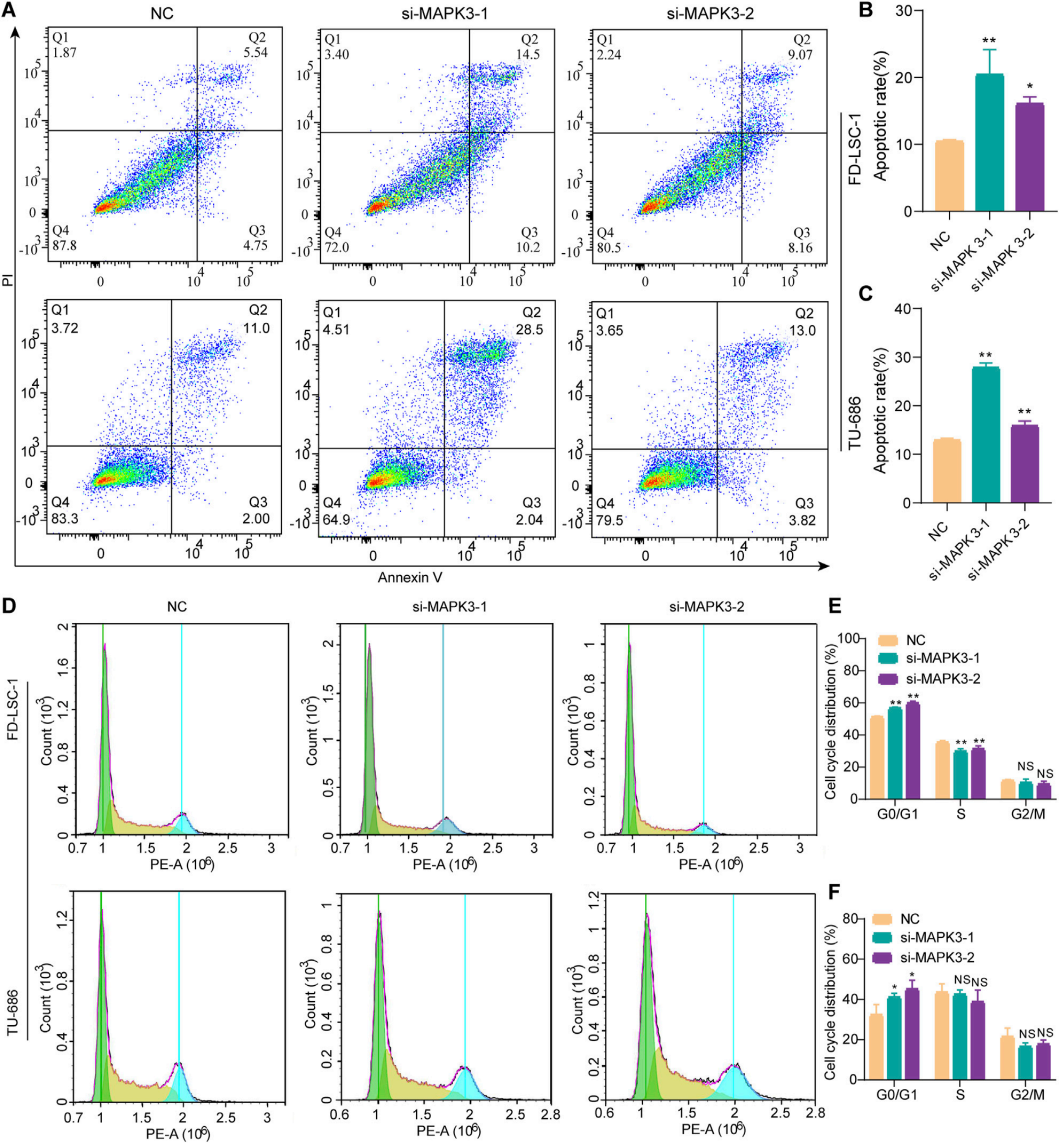
Effect of MAPK3 knockdown on apoptosis and cell cycle of LSCC cells. **(A–C)** The percentage of apoptotic cells was detected by flow cytometry after transfection of FD-LSC-1 and TU-686 cells with si-MAPK3. **(D–F)** FD-LSC-1 and TU-686 cells were transfected with si-MAPK3, PI staining was performed, and the cell cycle distribution was detected by flow cytometry. Error bars represent the SD of three independent experiments. *p < 0.05, **p < 0.01.

The effect of MAPK3 knockdown on apoptosis in LSCC was examined by flow cytometry. The results showed that MAPK3 knockdown significantly increased apoptosis rates compared with the control group (Figures 7A–C). These results indicated that inhibition of MAPK3 induces apoptosis in LSCC cells. In addition, the results of cell cycle analysis showed that cells in the si-MAPK3 group were significantly accumulated in the G0/G1 phase, compared with the control group (Figures 7D–F). This suggests that knockdown of MAPK3 not only promotes apoptosis but also may inhibit cell proliferation by regulating the cell cycle.

## 4 Discussion

LSCC is a common malignant tumor in the head and neck (Li Y. et al., 2022), and its occurrence is closely associated with a variety of risk factors, including smoking and alcohol abuse (Liberale et al., 2023). Early symptoms of LSCC patients are not obvious, resulting in most patients being in advanced stages when diagnosed (Patel et al., 2019). For patients with advanced stages, treatment usually requires partial or total laryngectomy combined with radiotherapy (Li D. et al., 2022). However, this type of treatment severely affects patients’ vocal and swallowing functions, leading to a significant decline in patients’ quality of life. It is also prone to drug resistance, which ultimately leads to treatment failure, a recurrence rate and other adverse consequences (Yang et al., 2023b). In current clinics, more effective and targeted therapeutic methods are urgently needed. Based on the potential antitumor effects of traditional Chinese medicine Codonopsis pilosula, this study explores its therapeutic effects on LSCC. It provides new pharmacological options for LSCC patients, reduces the side effects associated with traditional treatments, and improves the quality of life and survival rate of patients.

Traditional Chinese medicines are widely known for their abundant resources, simple concoctions, precise therapeutic effects and high economic benefits. However, due to the complexity of its composition, the specific mechanism of action of its single active ingredient has not been fully elucidated. This limits the promotion of Chinese medicine worldwide (Cao M. et al., 2019). As a traditional Chinese medicine with a long history, Codonopsis pilosula has attracted widespread attention in the medical field in recent years (Guo et al., 2024b). Codonopsis pilosula has demonstrated a significant role in the prevention and treatment of a variety of cancers. It has been shown to exert significant therapeutic effects in diseases such as colon cancer (Cao Y. et al., 2019), gastric precancerous lesions (He et al., 2022), and hepatocellular carcinoma (Liu et al., 2022). In this study, based on the traditional Chinese medicine database and combined with cytoscape software, we constructed the effective compound-target interaction network of Codonopsis pilosula and screened 22 potential targets of Codonopsis pilosula that might be directed against LSCC by cross-analyzing them with LSCC-related disease databases. In further analysis of the TCGA database and GEO database (GSE127165, GSE143224, GSE51985), only MAPK3 was significantly different, but showed low expression in LSCC. However, the expression of MAPK3 showed an upregulation trend in the clinical samples examined by qPCR in this study. MAPK3, also known as extracellular signal-regulated kinase 1 (ERK1), is a protein-coding gene belonging to the MAP kinase family (Wang X. et al., 2022), which is responsible for mediating the transmission of signals from the extracellular to the intracellular compartments, thereby regulating a series of cellular processes, including apoptosis, cell proliferation and migration (Flores et al., 2019; Liu et al., 2023). High expression of MAPK3 plays an active role in several signaling pathways related to cell proliferation as well as differentiation (Shi et al., 2021; Wang X. et al., 2022). MAPK3, as a critical component involved in the MAPK/ERK cascade pathway (Yang et al., 2019), and inhibition of MAPK3 can significantly inhibit cancer-stroma interactions and tumor metastasis (Yan et al., 2019). In addition, studies have shown that MAPK3 is involved in diabetic retinopathy development through autophagy regulation (Wang N. et al., 2022). Wang et al. Researchers found that cryptotanshinone, as a compound implicated in apoptosis, enhances MAPK3 expression, while inhibition of the MAPK signaling pathway can reverse cryptotanshinone-mediated apoptosis in cardiomyocytes (Wang et al., 2021). In this study, the proliferation, migration and invasion ability of LSCC cell lines was significantly reduced by knockdown of MAPK3. Meanwhile, knockdown of MAPK3 effectively induced apoptosis in LSCC cells and blocked the cell cycle at the G0/G1 phase, which in turn inhibited cell proliferation. These results suggest that MAPK3 has a pro-carcinogenic role in LSCC. Based on this, we hypothesized that MAPK3 expression is upregulated in LSCC patients and plays a role in disease progression.

Although there was some contradiction between the differences between MAPK3 in this study and those in TCGA and GEO databases. This might be related to group differences in sample sources, sample composition, different stages of tumor progression, and differences in experimental techniques and data processing methods. And the expression of MAPK3 was significantly reduced under different concentrations of Codonopsis pilosula treatment, although a clear concentration dependence was not presented. This result may be affected by the time of action or the concentration of action, which also provides an additional direction for subsequent studies. However, this does not affect MAPK3’s role as a key target for Codonopsis pilosula in LSCC treatment. Therefore, MAPK3 was established as a key herbal target of Codonopsis pilosula in the treatment of LSCC. This suggests that Codonopsis pilosula has potential application in the treatment of the MAPK3 target.

Codonopsis pilosula and MAPK3 have highly complex and diverse mechanisms of action. In existing studies, Codonopsis pilosula has been widely demonstrated to influence diseases through multi-components, multi-targets, and multi-pathways. For example, in the studies of esophageal cancer (Tian and Tang, 2022) and hepatocellular carcinoma (Liu et al., 2022), Codonopsis pilosula has been shown to exert anti-tumor effects by regulating multiple targets and multiple signaling pathways. Codonopsis pilosula’s multi-targeting effect makes it a potential multifunctional natural drug. In the present study, network pharmacological analysis revealed that Codonopsis pilosula might affect LSCC development by modulating several key signaling pathways. These include HIF-1, TNF, IL-17 and FoxO signaling pathways. Among them, HIF-1 adapts cancer cells to the local hypoxic environment and pre-adapts them to proliferate at distant metastatic sites by promoting anaerobic metabolism, cell survival mechanisms and angiogenesis, while triggering epithelial to mesenchymal transition through epigenetic reprogramming to induce cancer metastasis (Bhattacharya et al., 2024). In bone-invasive pituitary adenomas, TNFα activates the MAPK pathway autocrinely and promotes MMP9 expression, further accelerating membrane invasion (Wu et al., 2024). In addition, Li et al. demonstrated that the IL-17 signaling pathway was significantly enriched for upregulated genes in the GEO LSCC database, and MAPK3 was one of the pivotal genes involved in this pathway (Qi et al., 2021). FOXO, as a true tumor suppressor, regulates cellular behaviors such as cell cycle arrest and apoptosis through transcriptional programs, thereby inhibiting disease progression (Lees et al., 2023). In addition, Codonopsis pilosula has shown effective anti-tumor activity in vivo experiments. For example, in the S180 solid tumor mouse model, a 100 mg/kg dose of Codonopsis glucosamine inhibited tumor growth more significantly than a 50 mg/kg dose, and was able to significantly elevate the expression of TNF-α(Fan et al., 2023). Therefore, future studies will continue to investigate the optimal dose of Codonopsis pilosula in LSCC and its fine regulation mechanism on multiple signaling pathways.

In this study, we preliminarily explored the potential role of Codonopsis pilosula in the treatment of LSCC through network pharmacology and experimental validation, and identified MAPK3 as a possible key target. However, there are still some limitations that need to be further investigated. First, Codonopsis pilosula’s chemical composition is complex, and its main active components and specific mechanisms of action have not been fully elucidated. In this study, we used LC-MS to analyze the composition of Codonopsis pilosula extracts, identified a variety of compounds, and classified them using the ClassFire algorithm, which showed that Codonopsis pilosula extracts are mainly rich in phenylpropanoids and polyketides, lipids and lipid-like molecules, organic oxygen compounds and organoheterocyclic compounds. In addition, we screened potentially active compounds related to LSCC in combination with network pharmacology analysis. We evaluated their interactions with MAPK3 by molecular docking analysis. The results showed that Apigenin, Caprylic Acid, Capsaicin, Emodin, Enanthic Acid, Lauric Acid, Luteolin, Nicotine, and Nonanoic Acid had strong binding free energy with MAPK3. However, further verification of the actual presence of these compounds in Codonopsis pilosula extracts by LC-MS analysis showed that only Caprylic Acid, Emodin and Luteolin were identified within the detection range. This result may be related to the fact that the content of some compounds in Codonopsis pilosula was lower than the detection limit, the differences in chemical composition among different batches and origins, or the effect of the extraction process.

Second, although the present study suggests that Codonopsis pilosula may regulate the biological behavior of LSCC cells through MAPK3, the actual mechanism of action of Codonopsis pilosula may involve multiple signaling pathways. The regulation of MAPK3 activity, as a key component of the MAPK/ERK signaling pathway, may be co-influenced by other signaling pathways. In addition, different classes of compounds contained in Codonopsis pilosula may affect the LSCC process through synergistic or antagonistic effects. Therefore, future studies should further explore the network of action of Codonopsis pilosula in LSCC. They should allso resolve its multi-omics regulatory mechanism by combining metabolomics and transcriptomics analyses. In addition, since commercial sources of Codonopsis pilosula were used in this study, and different sources, batches and preparation processes may lead to differences in chemical compositions, which in turn may affect the reproducibility of the experimental results, future studies should further standardize the sources and quality control of Codonopsis pilosula to ensure the stability and generalizability of the results.

In addition, Codonopsis pilosula’s role in traditional Chinese medicine treatment is often achieved by combining it with other herbs to achieve optimal efficacy. In many traditional prescriptions, Codonopsis pilosula is usually paired with other herbs in specific ratios (Ma et al., 2023; Meng et al., 2023). Therefore, it is worth further research and exploration to see whether Codonopsis pilosula can exert more significant antitumor effects when paired with other herbs in LSCC treatment. In the future, the in-depth analysis of the pairing scheme based on Codonopsis pilosula and its mechanism of tumorigenesis and development will provide new research ideas and therapeutic strategies for the comprehensive treatment of LSCC.

## 5 Conclusion

In this study, we screened 96 compounds and 697 targets of Codonopsis pilosula by network pharmacology. We also screened 22 targets related to LSCC by combining the DisGeNET and GeneCards databases. KEGG pathway enrichment analysis showed that Codonopsis pilosula targets were involved in multiple cancer-related pathways. Further combined with LC-MS analysis, a variety of compounds were identified in Codonopsis pilosula extracts. Among them, Caprylic Acid, Emodin and Luteolin were detected in LC-MS analysis and showed strong binding ability to MAPK3, which further supported the hypothesis that Codonopsis pilosula might regulate LSCC cells’ biological behavior through MAPK3. In addition, MAPK3 knockdown significantly inhibited cell proliferation, migration and invasion, and induced apoptosis.

In summary, this study preliminarily explored the potential role of Codonopsis pilosula in the treatment of LSCC at both the levels of network pharmacology and experimental validation, revealing that it may affect the occurrence and development of LSCC through the modulation of MAPK3, which provides a new research direction for the treatment of LSCC. However, the complexity Codonopsis pilosula’s chemical composition and the specific regulatory mechanisms of its different components on MAPK3 still need further study. Future studies should be based on the quality control of standardized Codonopsis pilosula to explore in depth the mechanism of action of its active components and their potential for clinical applications.

## Data Availability

The data presented in the study are deposited in the OMIX repository, accession number OMIX009804, and are publicly available at: https://ngdc.cncb.ac.cn/omix/release/OMIX009804.
