# Supplementary material for: The mechanistic study of codonopsis pilosula on laryngeal squamous cell carcinoma based on network pharmacology and experimental validation
Source: Front Pharmacol. 2025 Apr 25;16:1542116. doi: 10.3389/fphar.2025.1542116 (PMC12061682; doi:10.3389/fphar.2025.1542116)
Supplement: Supplementary file 1 [file DataSheet1.zip › Supplementary Material/Supplementary_Figure S5.docx]

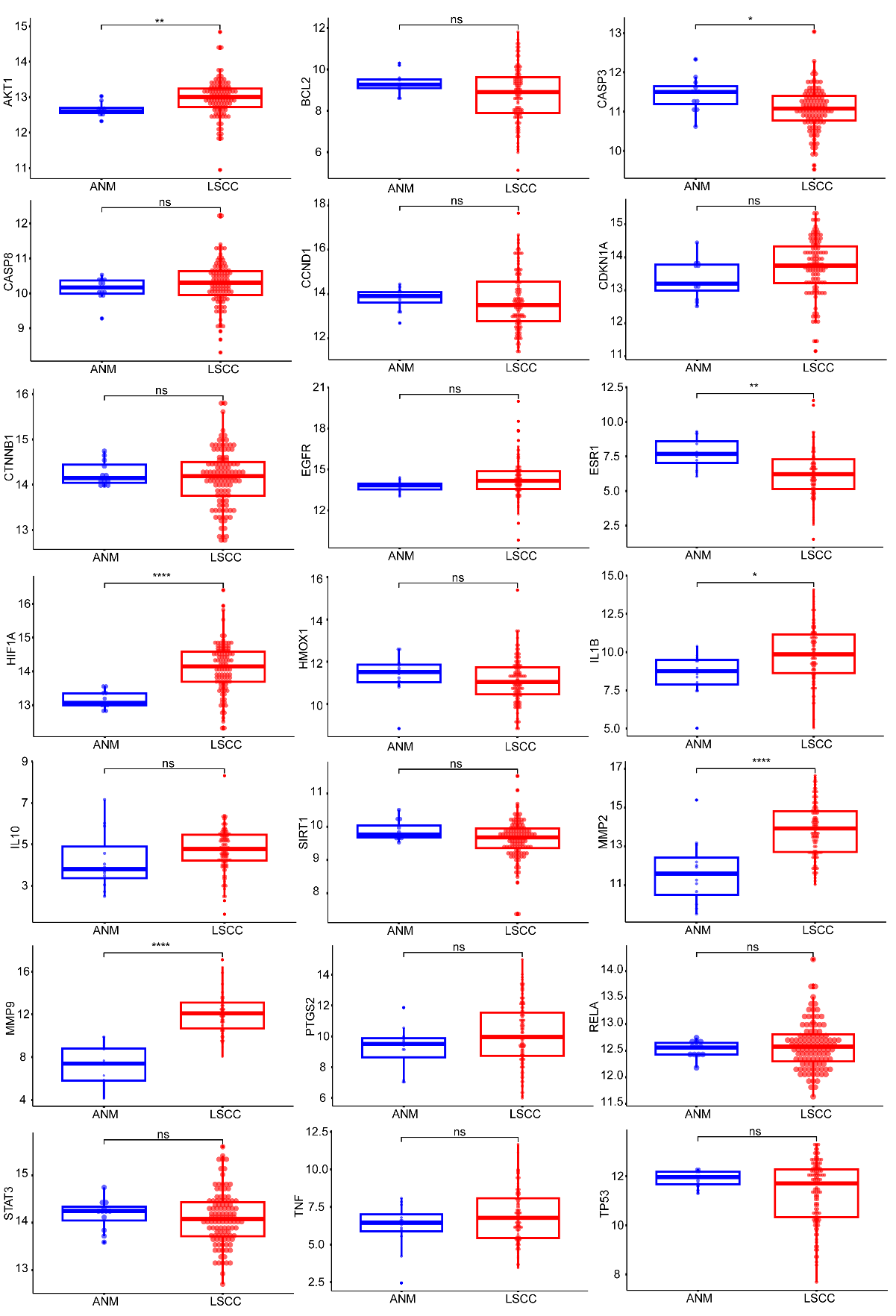
**Supplementary Figure S5.** The expression of 21 codonopsis pilosula-LSCC targets in the TCGA database
