# Supplementary material for: The mechanistic study of codonopsis pilosula on laryngeal squamous cell carcinoma based on network pharmacology and experimental validation
Source: Front Pharmacol. 2025 Apr 25;16:1542116. doi: 10.3389/fphar.2025.1542116 (PMC12061682; doi:10.3389/fphar.2025.1542116)
Supplement: Supplementary file 1 [file DataSheet1.zip › Supplementary Material/Supplementary_Table S1.docx]

**Supplementary Table S1.** Clinical information of 14 LSCC patients samples for qPCR of MAPK3.

| NO. | Age | Gender | T Stage | Cervical lymph node metastasis | Clinical stage |
| --- | --- | --- | --- | --- | --- |
| 1 | 66 | female | T3 | N+ | IV |
| 2 | 65 | female | T4 | N0 | IV |
| 3 | 70 | female | T4 | N+ | IV |
| 4 | 77 | female | T4 | N+ | IV |
| 5 | 67 | female | T4 | N0 | IV |
| 6 | 76 | female | T4 | N+ | IV |
| 7 | 65 | female | T3 | N0 | III |
| 8 | 61 | female | T3 | N0 | III |
| 9 | 65 | female | T3 | N+ | III |
| 10 | 61 | female | T2 | N0 | II |
| 11 | 64 | female | T3 | N0 | III |
| 12 | 57 | female | T3 | N0 | III |
| 13 | 71 | female | T4 | N0 | IV |
| 14 | 56 | female | T1 | N0 | I |
