# Supplementary material for: The mechanistic study of codonopsis pilosula on laryngeal squamous cell carcinoma based on network pharmacology and experimental validation
Source: Front Pharmacol. 2025 Apr 25;16:1542116. doi: 10.3389/fphar.2025.1542116 (PMC12061682; doi:10.3389/fphar.2025.1542116)
Supplement: Supplementary file 1 [file DataSheet1.zip › Supplementary Material/Supplementary_Table S2.docx]

**Supplementary Table S2.** Effective compounds and targets of Codonopsis pilosula.

| **Chemical compound** | **Target name** | **String gene mapping** |
| --- | --- | --- |
| [poriferasta-7,22E-dien-3beta-ol](https://tcmsp-e.com/molecule.php?qn=1006) | [Progesterone receptor](https://tcmsp-e.com/target.php?qt=209) | PGR |
| [poriferasta-7,22E-dien-3beta-ol](https://tcmsp-e.com/molecule.php?qn=1006) | [Nuclear receptor coactivator 2](https://tcmsp-e.com/target.php?qt=3276) | NCOA2 |
| [poriferasta-7,22E-dien-3beta-ol](https://tcmsp-e.com/molecule.php?qn=1006) | [Mineralocorticoid receptor](https://tcmsp-e.com/target.php?qt=252) | NR3C2 |
| [2-methoxyfuranodiene](https://tcmsp-e.com/molecule.php?qn=1160) | [Muscarinic acetylcholine receptor M3](https://tcmsp-e.com/target.php?qt=16) | CHRM3 |
| [2-methoxyfuranodiene](https://tcmsp-e.com/molecule.php?qn=1160) | [Thrombin](https://tcmsp-e.com/target.php?qt=17) | SERPIND1 |
| [2-methoxyfuranodiene](https://tcmsp-e.com/molecule.php?qn=1160) | [Muscarinic acetylcholine receptor M1](https://tcmsp-e.com/target.php?qt=38) | CHRM1 |
| [2-methoxyfuranodiene](https://tcmsp-e.com/molecule.php?qn=1160) | [Androgen receptor](https://tcmsp-e.com/target.php?qt=48) | AR |
| [2-methoxyfuranodiene](https://tcmsp-e.com/molecule.php?qn=1160) | [Nitric-oxide synthase, endothelial](https://tcmsp-e.com/target.php?qt=95) | NOS3 |
| [2-methoxyfuranodiene](https://tcmsp-e.com/molecule.php?qn=1160) | [Retinoic acid receptor RXR-alpha](https://tcmsp-e.com/target.php?qt=158) | RXRA |
| [2-methoxyfuranodiene](https://tcmsp-e.com/molecule.php?qn=1160) | [5-hydroxytryptamine 2A receptor](https://tcmsp-e.com/target.php?qt=175) | HTR2A |
| [2-methoxyfuranodiene](https://tcmsp-e.com/molecule.php?qn=1160) | [Muscarinic acetylcholine receptor M2](https://tcmsp-e.com/target.php?qt=210) | CHRM2 |
| [2-methoxyfuranodiene](https://tcmsp-e.com/molecule.php?qn=1160) | [Beta-2 adrenergic receptor](https://tcmsp-e.com/target.php?qt=261) | ADRB2 |
| [2-methoxyfuranodiene](https://tcmsp-e.com/molecule.php?qn=1160) | [Sodium-dependent serotonin transporter](https://tcmsp-e.com/target.php?qt=290) | SLC6A4 |
| [2-methoxyfuranodiene](https://tcmsp-e.com/molecule.php?qn=1160) | [Beta-1 adrenergic receptor](https://tcmsp-e.com/target.php?qt=63) | ADRB1 |
| [2-methoxyfuranodiene](https://tcmsp-e.com/molecule.php?qn=1160) | [Prostaglandin G/H synthase 2](https://tcmsp-e.com/target.php?qt=94) | PTGS2 |
| [2-methoxyfuranodiene](https://tcmsp-e.com/molecule.php?qn=1160) | [CGMP-inhibited 3',5'-cyclic phosphodiesterase A](https://tcmsp-e.com/target.php?qt=172) | PDE3A |
| [2-methoxyfuranodiene](https://tcmsp-e.com/molecule.php?qn=1160) | [Alpha-1A adrenergic receptor](https://tcmsp-e.com/target.php?qt=191) | ADRA1D |
| [2-methoxyfuranodiene](https://tcmsp-e.com/molecule.php?qn=1160) | [Alpha-1B adrenergic receptor](https://tcmsp-e.com/target.php?qt=216) | ADRA1B |
| [2-methoxyfuranodiene](https://tcmsp-e.com/molecule.php?qn=1160) | [Sodium-dependent dopamine transporter](https://tcmsp-e.com/target.php?qt=239) | SLC6A3 |
| [2-methoxyfuranodiene](https://tcmsp-e.com/molecule.php?qn=1160) | [Gamma-aminobutyric acid receptor subunit alpha-1](https://tcmsp-e.com/target.php?qt=309) | GABRA1 |
| [EIC](https://tcmsp-e.com/molecule.php?qn=131) | [Prostaglandin G/H synthase 1](https://tcmsp-e.com/target.php?qt=6) | PTGS1 |
| [EIC](https://tcmsp-e.com/molecule.php?qn=131) | [Prostaglandin G/H synthase 2](https://tcmsp-e.com/target.php?qt=94) | PTGS2 |
| [EIC](https://tcmsp-e.com/molecule.php?qn=131) | [Retinoic acid receptor RXR-alpha](https://tcmsp-e.com/target.php?qt=158) | RXRA |
| [EIC](https://tcmsp-e.com/molecule.php?qn=131) | [Nuclear receptor coactivator 2](https://tcmsp-e.com/target.php?qt=3276) | NCOA2 |
| [EIC](https://tcmsp-e.com/molecule.php?qn=131) | [Lysozyme](https://tcmsp-e.com/target.php?qt=1172) | LYZ |
| [EIC](https://tcmsp-e.com/molecule.php?qn=131) | [Sodium-dependent noradrenaline transporter](https://tcmsp-e.com/target.php?qt=186) | SLC6A2 |
| [EIC](https://tcmsp-e.com/molecule.php?qn=131) | [Gamma-aminobutyric-acid receptor alpha-2 subunit](https://tcmsp-e.com/target.php?qt=141) |  |
| [EIC](https://tcmsp-e.com/molecule.php?qn=131) | [Gamma-aminobutyric acid receptor subunit alpha-1](https://tcmsp-e.com/target.php?qt=309) | GABRA1 |
| [EIC](https://tcmsp-e.com/molecule.php?qn=131) | [Transient receptor potential cation channel subfamily V member 1](https://tcmsp-e.com/target.php?qt=404) | TRPV1 |
| [EIC](https://tcmsp-e.com/molecule.php?qn=131) | [Muscarinic acetylcholine receptor M1](https://tcmsp-e.com/target.php?qt=38) | CHRM1 |
| [EIC](https://tcmsp-e.com/molecule.php?qn=131) | [Muscarinic acetylcholine receptor M2](https://tcmsp-e.com/target.php?qt=210) | CHRM2 |
| [METHYL LINOLEATE](https://tcmsp-e.com/molecule.php?qn=1641) | [Prostaglandin G/H synthase 1](https://tcmsp-e.com/target.php?qt=6) | PTGS1 |
| [METHYL LINOLEATE](https://tcmsp-e.com/molecule.php?qn=1641) | [Prostaglandin G/H synthase 2](https://tcmsp-e.com/target.php?qt=94) | PTGS2 |
| [METHYL LINOLEATE](https://tcmsp-e.com/molecule.php?qn=1641) | [Nuclear receptor coactivator 2](https://tcmsp-e.com/target.php?qt=3276) | NCOA2 |
| [METHYL LINOLEATE](https://tcmsp-e.com/molecule.php?qn=1641) | [Retinoic acid receptor RXR-alpha](https://tcmsp-e.com/target.php?qt=158) | RXRA |
| [Perlolyrine](https://tcmsp-e.com/molecule.php?qn=2140) | [Thrombin](https://tcmsp-e.com/target.php?qt=17) | SERPIND1 |
| [Perlolyrine](https://tcmsp-e.com/molecule.php?qn=2140) | [Prostaglandin G/H synthase 2](https://tcmsp-e.com/target.php?qt=94) | PTGS2 |
| [Perlolyrine](https://tcmsp-e.com/molecule.php?qn=2140) | [Retinoic acid receptor RXR-alpha](https://tcmsp-e.com/target.php?qt=158) | RXRA |
| [Diop](https://tcmsp-e.com/molecule.php?qn=2879) | [Sodium channel protein type 5 subunit alpha](https://tcmsp-e.com/target.php?qt=70) | SCN5A |
| [Diop](https://tcmsp-e.com/molecule.php?qn=2879) | [Beta-2 adrenergic receptor](https://tcmsp-e.com/target.php?qt=261) | ADRB2 |
| [Diop](https://tcmsp-e.com/molecule.php?qn=2879) | [Muscarinic acetylcholine receptor M3](https://tcmsp-e.com/target.php?qt=16) | CHRM3 |
| [ZINC03978781](https://tcmsp-e.com/molecule.php?qn=3036) | [Progesterone receptor](https://tcmsp-e.com/target.php?qt=209) | PGR |
| [ZINC03978781](https://tcmsp-e.com/molecule.php?qn=3036) | [Nuclear receptor coactivator 2](https://tcmsp-e.com/target.php?qt=3276) | NCOA2 |
| [ZINC03978781](https://tcmsp-e.com/molecule.php?qn=3036) | [Mineralocorticoid receptor](https://tcmsp-e.com/target.php?qt=252) | NR3C2 |
| [Stigmasterol](https://tcmsp-e.com/molecule.php?qn=449) | [Progesterone receptor](https://tcmsp-e.com/target.php?qt=209) | PGR |
| [Stigmasterol](https://tcmsp-e.com/molecule.php?qn=449) | [Mineralocorticoid receptor](https://tcmsp-e.com/target.php?qt=252) | NR3C2 |
| [Stigmasterol](https://tcmsp-e.com/molecule.php?qn=449) | [Nuclear receptor coactivator 2](https://tcmsp-e.com/target.php?qt=3276) | NCOA2 |
| [Stigmasterol](https://tcmsp-e.com/molecule.php?qn=449) | [Alcohol dehydrogenase 1C](https://tcmsp-e.com/target.php?qt=727) | ADH1C |
| [Stigmasterol](https://tcmsp-e.com/molecule.php?qn=449) | [Retinoic acid receptor RXR-alpha](https://tcmsp-e.com/target.php?qt=158) | RXRA |
| [Stigmasterol](https://tcmsp-e.com/molecule.php?qn=449) | [Nuclear receptor coactivator 1](https://tcmsp-e.com/target.php?qt=3279) | NCOA1 |
| [Stigmasterol](https://tcmsp-e.com/molecule.php?qn=449) | [Prostaglandin G/H synthase 1](https://tcmsp-e.com/target.php?qt=6) | PTGS1 |
| [Stigmasterol](https://tcmsp-e.com/molecule.php?qn=449) | [Prostaglandin G/H synthase 2](https://tcmsp-e.com/target.php?qt=94) | PTGS2 |
| [Stigmasterol](https://tcmsp-e.com/molecule.php?qn=449) | [Alpha-2A adrenergic receptor](https://tcmsp-e.com/target.php?qt=105) | ADRA2A |
| [Stigmasterol](https://tcmsp-e.com/molecule.php?qn=449) | [Sodium-dependent noradrenaline transporter](https://tcmsp-e.com/target.php?qt=186) | SLC6A2 |
| [Stigmasterol](https://tcmsp-e.com/molecule.php?qn=449) | [Sodium-dependent dopamine transporter](https://tcmsp-e.com/target.php?qt=239) | SLC6A3 |
| [Stigmasterol](https://tcmsp-e.com/molecule.php?qn=449) | [Beta-2 adrenergic receptor](https://tcmsp-e.com/target.php?qt=261) | ADRB2 |
| [Stigmasterol](https://tcmsp-e.com/molecule.php?qn=449) | [Aldose reductase](https://tcmsp-e.com/target.php?qt=288) | AKR1B1 |
| [Stigmasterol](https://tcmsp-e.com/molecule.php?qn=449) | [Urokinase-type plasminogen activator](https://tcmsp-e.com/target.php?qt=346) | PLAU |
| [Stigmasterol](https://tcmsp-e.com/molecule.php?qn=449) | [Leukotriene A-4 hydrolase](https://tcmsp-e.com/target.php?qt=521) | LTA4H |
| [Stigmasterol](https://tcmsp-e.com/molecule.php?qn=449) | [Amine oxidase [flavin-containing] B](https://tcmsp-e.com/target.php?qt=565) | MAOB |
| [Stigmasterol](https://tcmsp-e.com/molecule.php?qn=449) | [Amine oxidase [flavin-containing] A](https://tcmsp-e.com/target.php?qt=566) | MAOA |
| [Stigmasterol](https://tcmsp-e.com/molecule.php?qn=449) | [Chymotrypsinogen B](https://tcmsp-e.com/target.php?qt=1696) | CTRB1 |
| [Stigmasterol](https://tcmsp-e.com/molecule.php?qn=449) | [Muscarinic acetylcholine receptor M3](https://tcmsp-e.com/target.php?qt=16) | CHRM3 |
| [Stigmasterol](https://tcmsp-e.com/molecule.php?qn=449) | [Muscarinic acetylcholine receptor M1](https://tcmsp-e.com/target.php?qt=38) | CHRM1 |
| [Stigmasterol](https://tcmsp-e.com/molecule.php?qn=449) | [Beta-1 adrenergic receptor](https://tcmsp-e.com/target.php?qt=63) | ADRB1 |
| [Stigmasterol](https://tcmsp-e.com/molecule.php?qn=449) | [Sodium channel protein type 5 subunit alpha](https://tcmsp-e.com/target.php?qt=70) | SCN5A |
| [Stigmasterol](https://tcmsp-e.com/molecule.php?qn=449) | [5-hydroxytryptamine 2A receptor](https://tcmsp-e.com/target.php?qt=175) | HTR2A |
| [Stigmasterol](https://tcmsp-e.com/molecule.php?qn=449) | [Alpha-1A adrenergic receptor](https://tcmsp-e.com/target.php?qt=191) | ADRA1D |
| [Stigmasterol](https://tcmsp-e.com/molecule.php?qn=449) | [Muscarinic acetylcholine receptor M2](https://tcmsp-e.com/target.php?qt=210) | CHRM2 |
| [Stigmasterol](https://tcmsp-e.com/molecule.php?qn=449) | [Alpha-1B adrenergic receptor](https://tcmsp-e.com/target.php?qt=216) | ADRA1B |
| [Stigmasterol](https://tcmsp-e.com/molecule.php?qn=449) | [Gamma-aminobutyric acid receptor subunit alpha-1](https://tcmsp-e.com/target.php?qt=309) | GABRA1 |
| [Shekanin](https://tcmsp-e.com/molecule.php?qn=3766) | [DNA topoisomerase II](https://tcmsp-e.com/target.php?qt=287) | TOP2B |
| [Shekanin](https://tcmsp-e.com/molecule.php?qn=3766) | [Coagulation factor VII](https://tcmsp-e.com/target.php?qt=123) | F7 |
| [tectorigenin](https://tcmsp-e.com/molecule.php?qn=3767) | [Nitric oxide synthase, inducible](https://tcmsp-e.com/target.php?qt=3) | NOS2 |
| [tectorigenin](https://tcmsp-e.com/molecule.php?qn=3767) | [Prostaglandin G/H synthase 1](https://tcmsp-e.com/target.php?qt=6) | PTGS1 |
| [tectorigenin](https://tcmsp-e.com/molecule.php?qn=3767) | [Estrogen receptor](https://tcmsp-e.com/target.php?qt=46) | ESR1 |
| [tectorigenin](https://tcmsp-e.com/molecule.php?qn=3767) | [Androgen receptor](https://tcmsp-e.com/target.php?qt=48) | AR |
| [tectorigenin](https://tcmsp-e.com/molecule.php?qn=3767) | [Peroxisome proliferator activated receptor gamma](https://tcmsp-e.com/target.php?qt=78) | PPARG |
| [tectorigenin](https://tcmsp-e.com/molecule.php?qn=3767) | [Prostaglandin G/H synthase 2](https://tcmsp-e.com/target.php?qt=94) | PTGS2 |
| [tectorigenin](https://tcmsp-e.com/molecule.php?qn=3767) | [Estrogen receptor beta](https://tcmsp-e.com/target.php?qt=307) | ESR2 |
| [tectorigenin](https://tcmsp-e.com/molecule.php?qn=3767) | [Mitogen-activated protein kinase 14](https://tcmsp-e.com/target.php?qt=402) | MAPK14 |
| [tectorigenin](https://tcmsp-e.com/molecule.php?qn=3767) | [Glycogen synthase kinase-3 beta](https://tcmsp-e.com/target.php?qt=422) | GSK3B |
| [tectorigenin](https://tcmsp-e.com/molecule.php?qn=3767) | [Heat shock protein HSP 90](https://tcmsp-e.com/target.php?qt=444) | HSP90AA1 |
| [tectorigenin](https://tcmsp-e.com/molecule.php?qn=3767) | [Cell division protein kinase 2](https://tcmsp-e.com/target.php?qt=482) | CDK2 |
| [tectorigenin](https://tcmsp-e.com/molecule.php?qn=3767) | [Serine/threonine-protein kinase Chk1](https://tcmsp-e.com/target.php?qt=647) | CHEK1 |
| [tectorigenin](https://tcmsp-e.com/molecule.php?qn=3767) | [Trypsin-1](https://tcmsp-e.com/target.php?qt=2928) | PRSS1 |
| [tectorigenin](https://tcmsp-e.com/molecule.php?qn=3767) | [Cyclin-A2](https://tcmsp-e.com/target.php?qt=3025) | CCNA2 |
| [tectorigenin](https://tcmsp-e.com/molecule.php?qn=3767) | [Calmodulin](https://tcmsp-e.com/target.php?qt=3907) | CALM3 |
| [tectorigenin](https://tcmsp-e.com/molecule.php?qn=3767) | [Retinoic acid receptor RXR-alpha](https://tcmsp-e.com/target.php?qt=158) | RXRA |
| [tectorigenin](https://tcmsp-e.com/molecule.php?qn=3767) | [Nuclear receptor coactivator 1](https://tcmsp-e.com/target.php?qt=3279) | NCOA1 |
| [tectorigenin](https://tcmsp-e.com/molecule.php?qn=3767) | [Telomerase protein component 1](https://tcmsp-e.com/target.php?qt=4549) | TEP1 |
| [tectorigenin](https://tcmsp-e.com/molecule.php?qn=3767) | [Interleukin-1 beta](https://tcmsp-e.com/target.php?qt=418) | IL1B |
| [tectorigenin](https://tcmsp-e.com/molecule.php?qn=3767) | [Prostaglandin E2 receptor EP3 subtype](https://tcmsp-e.com/target.php?qt=593) | PTGER3 |
| [tectorigenin](https://tcmsp-e.com/molecule.php?qn=3767) | [Puromycin-sensitive aminopeptidase](https://tcmsp-e.com/target.php?qt=4484) | NPEPPS |
| [tectorigenin](https://tcmsp-e.com/molecule.php?qn=3767) | [Aldose reductase](https://tcmsp-e.com/target.php?qt=288) | AKR1B1 |
| [tectorigenin](https://tcmsp-e.com/molecule.php?qn=3767) | [SAM pointed domain-containing Ets transcription factor](https://tcmsp-e.com/target.php?qt=4507) | SPDEF |
| [tectorigenin](https://tcmsp-e.com/molecule.php?qn=3767) | [Telomerase reverse transcriptase](https://tcmsp-e.com/target.php?qt=4550) | TERT |
| [tectorigenin](https://tcmsp-e.com/molecule.php?qn=3767) | [Metalloproteinase inhibitor 3](https://tcmsp-e.com/target.php?qt=4343) | TIMP3 |
| [7-Methoxy-2-methyl isoflavone](https://tcmsp-e.com/molecule.php?qn=3896) | [Nitric oxide synthase, inducible](https://tcmsp-e.com/target.php?qt=3) | NOS2 |
| [7-Methoxy-2-methyl isoflavone](https://tcmsp-e.com/molecule.php?qn=3896) | [Prostaglandin G/H synthase 1](https://tcmsp-e.com/target.php?qt=6) | PTGS1 |
| [7-Methoxy-2-methyl isoflavone](https://tcmsp-e.com/molecule.php?qn=3896) | [Dopamine D1 receptor](https://tcmsp-e.com/target.php?qt=7) | DRD1 |
| [7-Methoxy-2-methyl isoflavone](https://tcmsp-e.com/molecule.php?qn=3896) | [Muscarinic acetylcholine receptor M3](https://tcmsp-e.com/target.php?qt=16) | CHRM3 |
| [7-Methoxy-2-methyl isoflavone](https://tcmsp-e.com/molecule.php?qn=3896) | [Thrombin](https://tcmsp-e.com/target.php?qt=17) | SERPIND1 |
| [7-Methoxy-2-methyl isoflavone](https://tcmsp-e.com/molecule.php?qn=3896) | [Muscarinic acetylcholine receptor M1](https://tcmsp-e.com/target.php?qt=38) | CHRM1 |
| [7-Methoxy-2-methyl isoflavone](https://tcmsp-e.com/molecule.php?qn=3896) | [Estrogen receptor](https://tcmsp-e.com/target.php?qt=46) | ESR1 |
| [7-Methoxy-2-methyl isoflavone](https://tcmsp-e.com/molecule.php?qn=3896) | [Androgen receptor](https://tcmsp-e.com/target.php?qt=48) | AR |
| [7-Methoxy-2-methyl isoflavone](https://tcmsp-e.com/molecule.php?qn=3896) | [Beta-1 adrenergic receptor](https://tcmsp-e.com/target.php?qt=63) | ADRB1 |
| [7-Methoxy-2-methyl isoflavone](https://tcmsp-e.com/molecule.php?qn=3896) | [Sodium channel protein type 5 subunit alpha](https://tcmsp-e.com/target.php?qt=70) | SCN5A |
| [7-Methoxy-2-methyl isoflavone](https://tcmsp-e.com/molecule.php?qn=3896) | [Peroxisome proliferator activated receptor gamma](https://tcmsp-e.com/target.php?qt=78) | PPARG |
| [7-Methoxy-2-methyl isoflavone](https://tcmsp-e.com/molecule.php?qn=3896) | [Prostaglandin G/H synthase 2](https://tcmsp-e.com/target.php?qt=94) | PTGS2 |
| [7-Methoxy-2-methyl isoflavone](https://tcmsp-e.com/molecule.php?qn=3896) | [Retinoic acid receptor RXR-alpha](https://tcmsp-e.com/target.php?qt=158) | RXRA |
| [7-Methoxy-2-methyl isoflavone](https://tcmsp-e.com/molecule.php?qn=3896) | [Acetylcholinesterase](https://tcmsp-e.com/target.php?qt=165) | ACHE |
| [7-Methoxy-2-methyl isoflavone](https://tcmsp-e.com/molecule.php?qn=3896) | [CGMP-inhibited 3',5'-cyclic phosphodiesterase A](https://tcmsp-e.com/target.php?qt=172) | PDE3A |
| [7-Methoxy-2-methyl isoflavone](https://tcmsp-e.com/molecule.php?qn=3896) | [Alpha-1B adrenergic receptor](https://tcmsp-e.com/target.php?qt=216) | ADRA1B |
| [7-Methoxy-2-methyl isoflavone](https://tcmsp-e.com/molecule.php?qn=3896) | [Sodium-dependent dopamine transporter](https://tcmsp-e.com/target.php?qt=239) | SLC6A3 |
| [7-Methoxy-2-methyl isoflavone](https://tcmsp-e.com/molecule.php?qn=3896) | [Beta-2 adrenergic receptor](https://tcmsp-e.com/target.php?qt=261) | ADRB2 |
| [7-Methoxy-2-methyl isoflavone](https://tcmsp-e.com/molecule.php?qn=3896) | [Alpha-1D adrenergic receptor](https://tcmsp-e.com/target.php?qt=272) | ADRA1D |
| [7-Methoxy-2-methyl isoflavone](https://tcmsp-e.com/molecule.php?qn=3896) | [Sodium-dependent serotonin transporter](https://tcmsp-e.com/target.php?qt=290) | SLC6A4 |
| [7-Methoxy-2-methyl isoflavone](https://tcmsp-e.com/molecule.php?qn=3896) | [Estrogen receptor beta](https://tcmsp-e.com/target.php?qt=307) | ESR2 |
| [7-Methoxy-2-methyl isoflavone](https://tcmsp-e.com/molecule.php?qn=3896) | [Gamma-aminobutyric acid receptor subunit alpha-1](https://tcmsp-e.com/target.php?qt=309) | GABRA1 |
| [7-Methoxy-2-methyl isoflavone](https://tcmsp-e.com/molecule.php?qn=3896) | [Dipeptidyl peptidase IV](https://tcmsp-e.com/target.php?qt=332) | DPP4 |
| [7-Methoxy-2-methyl isoflavone](https://tcmsp-e.com/molecule.php?qn=3896) | [Mitogen-activated protein kinase 14](https://tcmsp-e.com/target.php?qt=402) | MAPK14 |
| [7-Methoxy-2-methyl isoflavone](https://tcmsp-e.com/molecule.php?qn=3896) | [Glycogen synthase kinase-3 beta](https://tcmsp-e.com/target.php?qt=422) | GSK3B |
| [7-Methoxy-2-methyl isoflavone](https://tcmsp-e.com/molecule.php?qn=3896) | [Heat shock protein HSP 90](https://tcmsp-e.com/target.php?qt=444) | HSP90AA1 |
| [7-Methoxy-2-methyl isoflavone](https://tcmsp-e.com/molecule.php?qn=3896) | [Cell division protein kinase 2](https://tcmsp-e.com/target.php?qt=482) | CDK2 |
| [7-Methoxy-2-methyl isoflavone](https://tcmsp-e.com/molecule.php?qn=3896) | [Leukotriene A-4 hydrolase](https://tcmsp-e.com/target.php?qt=521) | LTA4H |
| [7-Methoxy-2-methyl isoflavone](https://tcmsp-e.com/molecule.php?qn=3896) | [Amine oxidase [flavin-containing] B](https://tcmsp-e.com/target.php?qt=565) | MAOB |
| [7-Methoxy-2-methyl isoflavone](https://tcmsp-e.com/molecule.php?qn=3896) | [Serine/threonine-protein kinase Chk1](https://tcmsp-e.com/target.php?qt=647) | CHEK1 |
| [7-Methoxy-2-methyl isoflavone](https://tcmsp-e.com/molecule.php?qn=3896) | [Trypsin-1](https://tcmsp-e.com/target.php?qt=2928) | PRSS1 |
| [7-Methoxy-2-methyl isoflavone](https://tcmsp-e.com/molecule.php?qn=3896) | [Cyclin-A2](https://tcmsp-e.com/target.php?qt=3025) | CCNA2 |
| [7-Methoxy-2-methyl isoflavone](https://tcmsp-e.com/molecule.php?qn=3896) | [Nuclear receptor coactivator 1](https://tcmsp-e.com/target.php?qt=3279) | NCOA1 |
| [7-Methoxy-2-methyl isoflavone](https://tcmsp-e.com/molecule.php?qn=3896) | [cAMP-dependent protein kinase inhibitor alpha](https://tcmsp-e.com/target.php?qt=3586) | PKIA |
| [7-Methoxy-2-methyl isoflavone](https://tcmsp-e.com/molecule.php?qn=3896) | [Calmodulin](https://tcmsp-e.com/target.php?qt=3907) | CALM3 |
| [7-Methoxy-2-methyl isoflavone](https://tcmsp-e.com/molecule.php?qn=3896) | [Muscarinic acetylcholine receptor M5](https://tcmsp-e.com/target.php?qt=87) | CHRM5 |
| [7-Methoxy-2-methyl isoflavone](https://tcmsp-e.com/molecule.php?qn=3896) | [Nitric-oxide synthase, endothelial](https://tcmsp-e.com/target.php?qt=95) | NOS3 |
| [7-Methoxy-2-methyl isoflavone](https://tcmsp-e.com/molecule.php?qn=3896) | [Mu-type opioid receptor](https://tcmsp-e.com/target.php?qt=299) | OPRM1 |
| [7-Methoxy-2-methyl isoflavone](https://tcmsp-e.com/molecule.php?qn=3896) | [Nuclear receptor coactivator 2](https://tcmsp-e.com/target.php?qt=3276) | NCOA2 |
| [Spinasterol](https://tcmsp-e.com/molecule.php?qn=4355) | [Progesterone receptor](https://tcmsp-e.com/target.php?qt=209) | PGR |
| [Spinasterol](https://tcmsp-e.com/molecule.php?qn=4355) | [Mineralocorticoid receptor](https://tcmsp-e.com/target.php?qt=252) | NR3C2 |
| [Spinasterol](https://tcmsp-e.com/molecule.php?qn=4355) | [Nuclear receptor coactivator 2](https://tcmsp-e.com/target.php?qt=3276) | NCOA2 |
| [atractylenolideII](https://tcmsp-e.com/molecule.php?qn=44) | [Gamma-aminobutyric acid receptor subunit alpha-1](https://tcmsp-e.com/target.php?qt=309) | GABRA1 |
| [atractylenolideII](https://tcmsp-e.com/molecule.php?qn=44) | [Glutamate receptor 2](https://tcmsp-e.com/target.php?qt=3216) | GRIA2 |
| [atractylenolide iii](https://tcmsp-e.com/molecule.php?qn=45) | [Gamma-aminobutyric acid receptor subunit alpha-1](https://tcmsp-e.com/target.php?qt=309) | GABRA1 |
| [atractylenolide iii](https://tcmsp-e.com/molecule.php?qn=45) | [Glutamate receptor 2](https://tcmsp-e.com/target.php?qt=3216) | GRIA2 |
| [atractylenolide iii](https://tcmsp-e.com/molecule.php?qn=45) | [Tumor necrosis factor](https://tcmsp-e.com/target.php?qt=265) | TNF |
| [Encecalin](https://tcmsp-e.com/molecule.php?qn=4623) | [Nitric oxide synthase, inducible](https://tcmsp-e.com/target.php?qt=3) | NOS2 |
| [Encecalin](https://tcmsp-e.com/molecule.php?qn=4623) | [Prostaglandin G/H synthase 1](https://tcmsp-e.com/target.php?qt=6) | PTGS1 |
| [Encecalin](https://tcmsp-e.com/molecule.php?qn=4623) | [Dopamine D1 receptor](https://tcmsp-e.com/target.php?qt=7) | DRD1 |
| [Encecalin](https://tcmsp-e.com/molecule.php?qn=4623) | [Muscarinic acetylcholine receptor M3](https://tcmsp-e.com/target.php?qt=16) | CHRM3 |
| [Encecalin](https://tcmsp-e.com/molecule.php?qn=4623) | [Muscarinic acetylcholine receptor M1](https://tcmsp-e.com/target.php?qt=38) | CHRM1 |
| [Encecalin](https://tcmsp-e.com/molecule.php?qn=4623) | [Estrogen receptor](https://tcmsp-e.com/target.php?qt=46) | ESR1 |
| [Encecalin](https://tcmsp-e.com/molecule.php?qn=4623) | [Sodium channel protein type 5 subunit alpha](https://tcmsp-e.com/target.php?qt=70) | SCN5A |
| [Encecalin](https://tcmsp-e.com/molecule.php?qn=4623) | [Prostaglandin G/H synthase 2](https://tcmsp-e.com/target.php?qt=94) | PTGS2 |
| [Encecalin](https://tcmsp-e.com/molecule.php?qn=4623) | [Nitric-oxide synthase, endothelial](https://tcmsp-e.com/target.php?qt=95) | NOS3 |
| [Encecalin](https://tcmsp-e.com/molecule.php?qn=4623) | [Alpha-2A adrenergic receptor](https://tcmsp-e.com/target.php?qt=105) | ADRA2A |
| [Encecalin](https://tcmsp-e.com/molecule.php?qn=4623) | [Muscarinic acetylcholine receptor M4](https://tcmsp-e.com/target.php?qt=154) | CHRM4 |
| [Encecalin](https://tcmsp-e.com/molecule.php?qn=4623) | [Retinoic acid receptor RXR-alpha](https://tcmsp-e.com/target.php?qt=158) | RXRA |
| [Encecalin](https://tcmsp-e.com/molecule.php?qn=4623) | [CGMP-inhibited 3',5'-cyclic phosphodiesterase A](https://tcmsp-e.com/target.php?qt=172) | PDE3A |
| [Encecalin](https://tcmsp-e.com/molecule.php?qn=4623) | [5-hydroxytryptamine 2A receptor](https://tcmsp-e.com/target.php?qt=175) | HTR2A |
| [Encecalin](https://tcmsp-e.com/molecule.php?qn=4623) | [Sodium-dependent noradrenaline transporter](https://tcmsp-e.com/target.php?qt=186) | SLC6A2 |
| [Encecalin](https://tcmsp-e.com/molecule.php?qn=4623) | [Alpha-1A adrenergic receptor](https://tcmsp-e.com/target.php?qt=191) | ADRA1D |
| [Encecalin](https://tcmsp-e.com/molecule.php?qn=4623) | [5-hydroxytryptamine 2C receptor](https://tcmsp-e.com/target.php?qt=203) | HTR2C |
| [Encecalin](https://tcmsp-e.com/molecule.php?qn=4623) | [Muscarinic acetylcholine receptor M2](https://tcmsp-e.com/target.php?qt=210) | CHRM2 |
| [Encecalin](https://tcmsp-e.com/molecule.php?qn=4623) | [Alpha-2B adrenergic receptor](https://tcmsp-e.com/target.php?qt=214) | ADRA2B |
| [Encecalin](https://tcmsp-e.com/molecule.php?qn=4623) | [Alpha-1B adrenergic receptor](https://tcmsp-e.com/target.php?qt=216) | ADRA1B |
| [Encecalin](https://tcmsp-e.com/molecule.php?qn=4623) | [Sodium-dependent dopamine transporter](https://tcmsp-e.com/target.php?qt=239) | SLC6A3 |
| [Encecalin](https://tcmsp-e.com/molecule.php?qn=4623) | [Beta-2 adrenergic receptor](https://tcmsp-e.com/target.php?qt=261) | ADRB2 |
| [Encecalin](https://tcmsp-e.com/molecule.php?qn=4623) | [Sodium-dependent serotonin transporter](https://tcmsp-e.com/target.php?qt=290) | SLC6A4 |
| [Encecalin](https://tcmsp-e.com/molecule.php?qn=4623) | [Mu-type opioid receptor](https://tcmsp-e.com/target.php?qt=299) | OPRM1 |
| [Encecalin](https://tcmsp-e.com/molecule.php?qn=4623) | [Gamma-aminobutyric acid receptor subunit alpha-1](https://tcmsp-e.com/target.php?qt=309) | GABRA1 |
| [Encecalin](https://tcmsp-e.com/molecule.php?qn=4623) | [Heat shock protein HSP 90](https://tcmsp-e.com/target.php?qt=444) | HSP90AA1 |
| [Encecalin](https://tcmsp-e.com/molecule.php?qn=4623) | [Beta-lactamase](https://tcmsp-e.com/target.php?qt=499) | LACTBL1 |
| [Encecalin](https://tcmsp-e.com/molecule.php?qn=4623) | [Glutamate receptor 2](https://tcmsp-e.com/target.php?qt=3216) | GRIA2 |
| [Encecalin](https://tcmsp-e.com/molecule.php?qn=4623) | [Muscarinic acetylcholine receptor M5](https://tcmsp-e.com/target.php?qt=87) | CHRM5 |
| [Frutinone A](https://tcmsp-e.com/molecule.php?qn=5321) | [Prostaglandin G/H synthase 1](https://tcmsp-e.com/target.php?qt=6) | PTGS1 |
| [Frutinone A](https://tcmsp-e.com/molecule.php?qn=5321) | [Thrombin](https://tcmsp-e.com/target.php?qt=17) | SERPIND1 |
| [Frutinone A](https://tcmsp-e.com/molecule.php?qn=5321) | [Androgen receptor](https://tcmsp-e.com/target.php?qt=48) | AR |
| [Frutinone A](https://tcmsp-e.com/molecule.php?qn=5321) | [Sodium channel protein type 5 subunit alpha](https://tcmsp-e.com/target.php?qt=70) | SCN5A |
| [Frutinone A](https://tcmsp-e.com/molecule.php?qn=5321) | [Peroxisome proliferator activated receptor gamma](https://tcmsp-e.com/target.php?qt=78) | PPARG |
| [Frutinone A](https://tcmsp-e.com/molecule.php?qn=5321) | [Prostaglandin G/H synthase 2](https://tcmsp-e.com/target.php?qt=94) | PTGS2 |
| [Frutinone A](https://tcmsp-e.com/molecule.php?qn=5321) | [Retinoic acid receptor RXR-alpha](https://tcmsp-e.com/target.php?qt=158) | RXRA |
| [Frutinone A](https://tcmsp-e.com/molecule.php?qn=5321) | [CGMP-inhibited 3',5'-cyclic phosphodiesterase A](https://tcmsp-e.com/target.php?qt=172) | PDE3A |
| [Frutinone A](https://tcmsp-e.com/molecule.php?qn=5321) | [Beta-2 adrenergic receptor](https://tcmsp-e.com/target.php?qt=261) | ADRB2 |
| [Frutinone A](https://tcmsp-e.com/molecule.php?qn=5321) | [Gamma-aminobutyric acid receptor subunit alpha-1](https://tcmsp-e.com/target.php?qt=309) | GABRA1 |
| [Frutinone A](https://tcmsp-e.com/molecule.php?qn=5321) | [Dipeptidyl peptidase IV](https://tcmsp-e.com/target.php?qt=332) | DPP4 |
| [Frutinone A](https://tcmsp-e.com/molecule.php?qn=5321) | [Heat shock protein HSP 90](https://tcmsp-e.com/target.php?qt=444) | HSP90AA1 |
| [Frutinone A](https://tcmsp-e.com/molecule.php?qn=5321) | [Acetylcholinesterase](https://tcmsp-e.com/target.php?qt=165) | ACHE |
| [luteolin](https://tcmsp-e.com/molecule.php?qn=6) | [Prostaglandin G/H synthase 1](https://tcmsp-e.com/target.php?qt=6) | PTGS1 |
| [luteolin](https://tcmsp-e.com/molecule.php?qn=6) | [Androgen receptor](https://tcmsp-e.com/target.php?qt=48) | AR |
| [luteolin](https://tcmsp-e.com/molecule.php?qn=6) | [Prostaglandin G/H synthase 2](https://tcmsp-e.com/target.php?qt=94) | PTGS2 |
| [luteolin](https://tcmsp-e.com/molecule.php?qn=6) | [Heat shock protein HSP 90](https://tcmsp-e.com/target.php?qt=444) | HSP90AA1 |
| [luteolin](https://tcmsp-e.com/molecule.php?qn=6) | [Trypsin-1](https://tcmsp-e.com/target.php?qt=2928) | PRSS1 |
| [luteolin](https://tcmsp-e.com/molecule.php?qn=6) | [Nuclear receptor coactivator 2](https://tcmsp-e.com/target.php?qt=3276) | NCOA2 |
| [luteolin](https://tcmsp-e.com/molecule.php?qn=6) | [Dipeptidyl peptidase IV](https://tcmsp-e.com/target.php?qt=332) | DPP4 |
| [luteolin](https://tcmsp-e.com/molecule.php?qn=6) | [Transcription factor p65](https://tcmsp-e.com/target.php?qt=4565) | RELA |
| [luteolin](https://tcmsp-e.com/molecule.php?qn=6) | [Epidermal growth factor receptor](https://tcmsp-e.com/target.php?qt=298) | EGFR |
| [luteolin](https://tcmsp-e.com/molecule.php?qn=6) | [RAC-alpha serine/threonine-protein kinase](https://tcmsp-e.com/target.php?qt=4490) | AKT1 |
| [luteolin](https://tcmsp-e.com/molecule.php?qn=6) | [Vascular endothelial growth factor A](https://tcmsp-e.com/target.php?qt=740) | VEGFC |
| [luteolin](https://tcmsp-e.com/molecule.php?qn=6) | [G1/S-specific cyclin-D1](https://tcmsp-e.com/target.php?qt=4214) | CCND1 |
| [luteolin](https://tcmsp-e.com/molecule.php?qn=6) | [Bcl-2-like protein 1](https://tcmsp-e.com/target.php?qt=4054) | BCL2L1 |
| [luteolin](https://tcmsp-e.com/molecule.php?qn=6) | [Cyclin-dependent kinase inhibitor 1](https://tcmsp-e.com/target.php?qt=4141) | CDKN1A |
| [luteolin](https://tcmsp-e.com/molecule.php?qn=6) | [Caspase-9](https://tcmsp-e.com/target.php?qt=4090) | CASP9 |
| [luteolin](https://tcmsp-e.com/molecule.php?qn=6) | [72 kDa type IV collagenase](https://tcmsp-e.com/target.php?qt=238) | MMP2 |
| [luteolin](https://tcmsp-e.com/molecule.php?qn=6) | [Matrix metalloproteinase-9](https://tcmsp-e.com/target.php?qt=4334) | MMP9 |
| [luteolin](https://tcmsp-e.com/molecule.php?qn=6) | [Mitogen-activated protein kinase 1](https://tcmsp-e.com/target.php?qt=354) | MAPK1 |
| [luteolin](https://tcmsp-e.com/molecule.php?qn=6) | [Interleukin-10](https://tcmsp-e.com/target.php?qt=4292) | IL10 |
| [luteolin](https://tcmsp-e.com/molecule.php?qn=6) | [Retinoblastoma-associated protein](https://tcmsp-e.com/target.php?qt=2915) | RB1 |
| [luteolin](https://tcmsp-e.com/molecule.php?qn=6) | [Cell division protein kinase 4](https://tcmsp-e.com/target.php?qt=573) | CDK4 |
| [luteolin](https://tcmsp-e.com/molecule.php?qn=6) | [Tumor necrosis factor](https://tcmsp-e.com/target.php?qt=265) | TNF |
| [luteolin](https://tcmsp-e.com/molecule.php?qn=6) | [Transcription factor AP-1](https://tcmsp-e.com/target.php?qt=414) | JUN |
| [luteolin](https://tcmsp-e.com/molecule.php?qn=6) | [Interleukin-6](https://tcmsp-e.com/target.php?qt=351) | IL6 |
| [luteolin](https://tcmsp-e.com/molecule.php?qn=6) | [Caspase-3](https://tcmsp-e.com/target.php?qt=4087) | CASP3 |
| [luteolin](https://tcmsp-e.com/molecule.php?qn=6) | [Cellular tumor antigen p53](https://tcmsp-e.com/target.php?qt=646) | TP53 |
| [luteolin](https://tcmsp-e.com/molecule.php?qn=6) | [NF-kappa-B inhibitor alpha](https://tcmsp-e.com/target.php?qt=4394) | NFKBIA |
| [luteolin](https://tcmsp-e.com/molecule.php?qn=6) | [Xanthine dehydrogenase/oxidase](https://tcmsp-e.com/target.php?qt=568) | XDH |
| [luteolin](https://tcmsp-e.com/molecule.php?qn=6) | [DNA topoisomerase 1](https://tcmsp-e.com/target.php?qt=1293) | TOP1 |
| [luteolin](https://tcmsp-e.com/molecule.php?qn=6) | [E3 ubiquitin-protein ligase Mdm2](https://tcmsp-e.com/target.php?qt=4179) | MDM2 |
| [luteolin](https://tcmsp-e.com/molecule.php?qn=6) | [Amyloid beta A4 protein](https://tcmsp-e.com/target.php?qt=648) | APP |
| [luteolin](https://tcmsp-e.com/molecule.php?qn=6) | [Interstitial collagenase](https://tcmsp-e.com/target.php?qt=353) | MMP1 |
| [luteolin](https://tcmsp-e.com/molecule.php?qn=6) | [Proliferating cell nuclear antigen](https://tcmsp-e.com/target.php?qt=4450) | PCNA |
| [luteolin](https://tcmsp-e.com/molecule.php?qn=6) | [Receptor tyrosine-protein kinase erbB-2](https://tcmsp-e.com/target.php?qt=4496) | ERBB2 |
| [luteolin](https://tcmsp-e.com/molecule.php?qn=6) | [Peroxisome proliferator-activated receptor gamma](https://tcmsp-e.com/target.php?qt=4422) | PPARG |
| [luteolin](https://tcmsp-e.com/molecule.php?qn=6) | [Heme oxygenase 1](https://tcmsp-e.com/target.php?qt=2132) | HMOX1 |
| [luteolin](https://tcmsp-e.com/molecule.php?qn=6) | [Caspase-7](https://tcmsp-e.com/target.php?qt=3575) | CASP7 |
| [luteolin](https://tcmsp-e.com/molecule.php?qn=6) | [Intercellular adhesion molecule 1](https://tcmsp-e.com/target.php?qt=4287) | ICAM1 |
| [luteolin](https://tcmsp-e.com/molecule.php?qn=6) | [Induced myeloid leukemia cell differentiation protein Mcl-1](https://tcmsp-e.com/target.php?qt=4269) | MCL1 |
| [luteolin](https://tcmsp-e.com/molecule.php?qn=6) | [Baculoviral IAP repeat-containing protein 5](https://tcmsp-e.com/target.php?qt=4044) | BIRC5 |
| [luteolin](https://tcmsp-e.com/molecule.php?qn=6) | [Interleukin-2](https://tcmsp-e.com/target.php?qt=3978) | IL2 |
| [luteolin](https://tcmsp-e.com/molecule.php?qn=6) | [G2/mitotic-specific cyclin-B1](https://tcmsp-e.com/target.php?qt=4219) | CCNB1 |
| [luteolin](https://tcmsp-e.com/molecule.php?qn=6) | [Tyrosinase](https://tcmsp-e.com/target.php?qt=4590) | TYR |
| [luteolin](https://tcmsp-e.com/molecule.php?qn=6) | [Interferon gamma](https://tcmsp-e.com/target.php?qt=365) | IFNG |
| [luteolin](https://tcmsp-e.com/molecule.php?qn=6) | [Interleukin-4](https://tcmsp-e.com/target.php?qt=4301) | IL4 |
| [luteolin](https://tcmsp-e.com/molecule.php?qn=6) | [DNA topoisomerase 2-alpha](https://tcmsp-e.com/target.php?qt=4172) | TOP2A |
| [luteolin](https://tcmsp-e.com/molecule.php?qn=6) | [Glutathione S-transferase P](https://tcmsp-e.com/target.php?qt=733) | GSTP1 |
| [luteolin](https://tcmsp-e.com/molecule.php?qn=6) | [Baculoviral IAP repeat-containing protein 4](https://tcmsp-e.com/target.php?qt=4043) | XIAP |
| [luteolin](https://tcmsp-e.com/molecule.php?qn=6) | [Solute carrier family 2, facilitated glucose transporter member 4](https://tcmsp-e.com/target.php?qt=4535) | SLC2A4 |
| [luteolin](https://tcmsp-e.com/molecule.php?qn=6) | [Insulin receptor](https://tcmsp-e.com/target.php?qt=11) | INSR |
| [luteolin](https://tcmsp-e.com/molecule.php?qn=6) | [CD40 ligand](https://tcmsp-e.com/target.php?qt=4107) | CD40LG |
| [luteolin](https://tcmsp-e.com/molecule.php?qn=6) | [Prostaglandin E synthase](https://tcmsp-e.com/target.php?qt=4456) | PTGES |
| [luteolin](https://tcmsp-e.com/molecule.php?qn=6) | [Kinetochore protein Nuf2](https://tcmsp-e.com/target.php?qt=4311) | NUF2 |
| [luteolin](https://tcmsp-e.com/molecule.php?qn=6) | [Adenylate cyclase type 2](https://tcmsp-e.com/target.php?qt=4015) | ADCY2 |
| [luteolin](https://tcmsp-e.com/molecule.php?qn=6) | [Hepatocyte growth factor receptor](https://tcmsp-e.com/target.php?qt=349) | MET |
| [stigmast-7-enol](https://tcmsp-e.com/molecule.php?qn=6774) | [Progesterone receptor](https://tcmsp-e.com/target.php?qt=209) | PGR |
| [stigmast-7-enol](https://tcmsp-e.com/molecule.php?qn=6774) | [Nuclear receptor coactivator 2](https://tcmsp-e.com/target.php?qt=3276) | NCOA2 |
| [3-beta-Hydroxymethyllenetanshiquinone](https://tcmsp-e.com/molecule.php?qn=7059) | [Dopamine D1 receptor](https://tcmsp-e.com/target.php?qt=7) | DRD1 |
| [3-beta-Hydroxymethyllenetanshiquinone](https://tcmsp-e.com/molecule.php?qn=7059) | [Thrombin](https://tcmsp-e.com/target.php?qt=17) | SERPIND1 |
| [3-beta-Hydroxymethyllenetanshiquinone](https://tcmsp-e.com/molecule.php?qn=7059) | [Muscarinic acetylcholine receptor M1](https://tcmsp-e.com/target.php?qt=38) | CHRM1 |
| [3-beta-Hydroxymethyllenetanshiquinone](https://tcmsp-e.com/molecule.php?qn=7059) | [Prostaglandin G/H synthase 2](https://tcmsp-e.com/target.php?qt=94) | PTGS2 |
| [3-beta-Hydroxymethyllenetanshiquinone](https://tcmsp-e.com/molecule.php?qn=7059) | [Carbonic anhydrase II](https://tcmsp-e.com/target.php?qt=117) | CA2 |
| [3-beta-Hydroxymethyllenetanshiquinone](https://tcmsp-e.com/molecule.php?qn=7059) | [Retinoic acid receptor RXR-alpha](https://tcmsp-e.com/target.php?qt=158) | RXRA |
| [3-beta-Hydroxymethyllenetanshiquinone](https://tcmsp-e.com/molecule.php?qn=7059) | [Delta-type opioid receptor](https://tcmsp-e.com/target.php?qt=163) | OPRD1 |
| [3-beta-Hydroxymethyllenetanshiquinone](https://tcmsp-e.com/molecule.php?qn=7059) | [Acetylcholinesterase](https://tcmsp-e.com/target.php?qt=165) | ACHE |
| [3-beta-Hydroxymethyllenetanshiquinone](https://tcmsp-e.com/molecule.php?qn=7059) | [Alpha-1A adrenergic receptor](https://tcmsp-e.com/target.php?qt=191) | ADRA1D |
| [3-beta-Hydroxymethyllenetanshiquinone](https://tcmsp-e.com/molecule.php?qn=7059) | [Beta-2 adrenergic receptor](https://tcmsp-e.com/target.php?qt=261) | ADRB2 |
| [3-beta-Hydroxymethyllenetanshiquinone](https://tcmsp-e.com/molecule.php?qn=7059) | [Mu-type opioid receptor](https://tcmsp-e.com/target.php?qt=299) | OPRM1 |
| [3-beta-Hydroxymethyllenetanshiquinone](https://tcmsp-e.com/molecule.php?qn=7059) | [Dipeptidyl peptidase IV](https://tcmsp-e.com/target.php?qt=332) | DPP4 |
| [3-beta-Hydroxymethyllenetanshiquinone](https://tcmsp-e.com/molecule.php?qn=7059) | [Heat shock protein HSP 90](https://tcmsp-e.com/target.php?qt=444) | HSP90AA1 |
| [3-beta-Hydroxymethyllenetanshiquinone](https://tcmsp-e.com/molecule.php?qn=7059) | [Trypsin-1](https://tcmsp-e.com/target.php?qt=2928) | PRSS1 |
| [3-beta-Hydroxymethyllenetanshiquinone](https://tcmsp-e.com/molecule.php?qn=7059) | [Nuclear receptor coactivator 1](https://tcmsp-e.com/target.php?qt=3279) | NCOA1 |
| [methyl icosa-11,14-dienoate](https://tcmsp-e.com/molecule.php?qn=7514) | [Nuclear receptor coactivator 2](https://tcmsp-e.com/target.php?qt=3276) | NCOA2 |
| [apigenin](https://tcmsp-e.com/molecule.php?qn=8) | [Prostaglandin G/H synthase 1](https://tcmsp-e.com/target.php?qt=6) | PTGS1 |
| [apigenin](https://tcmsp-e.com/molecule.php?qn=8) | [Androgen receptor](https://tcmsp-e.com/target.php?qt=48) | AR |
| [apigenin](https://tcmsp-e.com/molecule.php?qn=8) | [Prostaglandin G/H synthase 2](https://tcmsp-e.com/target.php?qt=94) | PTGS2 |
| [apigenin](https://tcmsp-e.com/molecule.php?qn=8) | [Heat shock protein HSP 90](https://tcmsp-e.com/target.php?qt=444) | HSP90AA1 |
| [apigenin](https://tcmsp-e.com/molecule.php?qn=8) | [Trypsin-1](https://tcmsp-e.com/target.php?qt=2928) | PRSS1 |
| [apigenin](https://tcmsp-e.com/molecule.php?qn=8) | [Nuclear receptor coactivator 2](https://tcmsp-e.com/target.php?qt=3276) | NCOA2 |
| [apigenin](https://tcmsp-e.com/molecule.php?qn=8) | [Sodium channel protein type 5 subunit alpha](https://tcmsp-e.com/target.php?qt=70) | SCN5A |
| [apigenin](https://tcmsp-e.com/molecule.php?qn=8) | [Coagulation factor Xa](https://tcmsp-e.com/target.php?qt=79) | F7 |
| [apigenin](https://tcmsp-e.com/molecule.php?qn=8) | [Coagulation factor VII](https://tcmsp-e.com/target.php?qt=123) | F7 |
| [apigenin](https://tcmsp-e.com/molecule.php?qn=8) | [DNA topoisomerase II](https://tcmsp-e.com/target.php?qt=287) | TOP2B |
| [apigenin](https://tcmsp-e.com/molecule.php?qn=8) | [Dipeptidyl peptidase IV](https://tcmsp-e.com/target.php?qt=332) | DPP4 |
| [apigenin](https://tcmsp-e.com/molecule.php?qn=8) | [Calmodulin](https://tcmsp-e.com/target.php?qt=3907) | CALM3 |
| [apigenin](https://tcmsp-e.com/molecule.php?qn=8) | [Transcription factor p65](https://tcmsp-e.com/target.php?qt=4565) | RELA |
| [apigenin](https://tcmsp-e.com/molecule.php?qn=8) | [RAC-alpha serine/threonine-protein kinase](https://tcmsp-e.com/target.php?qt=4490) | AKT1 |
| [apigenin](https://tcmsp-e.com/molecule.php?qn=8) | [Vascular endothelial growth factor A](https://tcmsp-e.com/target.php?qt=740) | VEGFC |
| [apigenin](https://tcmsp-e.com/molecule.php?qn=8) | [G1/S-specific cyclin-D1](https://tcmsp-e.com/target.php?qt=4214) | CCND1 |
| [apigenin](https://tcmsp-e.com/molecule.php?qn=8) | [Apoptosis regulator Bcl-2](https://tcmsp-e.com/target.php?qt=86) | BCL2 |
| [apigenin](https://tcmsp-e.com/molecule.php?qn=8) | [Bcl-2-like protein 1](https://tcmsp-e.com/target.php?qt=4054) | BCL2L1 |
| [apigenin](https://tcmsp-e.com/molecule.php?qn=8) | [Proto-oncogene c-Fos](https://tcmsp-e.com/target.php?qt=4478) | FOS |
| [apigenin](https://tcmsp-e.com/molecule.php?qn=8) | [Cyclin-dependent kinase inhibitor 1](https://tcmsp-e.com/target.php?qt=4141) | CDKN1A |
| [apigenin](https://tcmsp-e.com/molecule.php?qn=8) | [Eukaryotic translation initiation factor 6](https://tcmsp-e.com/target.php?qt=4197) | EIF6 |
| [apigenin](https://tcmsp-e.com/molecule.php?qn=8) | [Apoptosis regulator BAX](https://tcmsp-e.com/target.php?qt=4033) | BAX |
| [apigenin](https://tcmsp-e.com/molecule.php?qn=8) | [Caspase-9](https://tcmsp-e.com/target.php?qt=4090) | CASP9 |
| [apigenin](https://tcmsp-e.com/molecule.php?qn=8) | [Urokinase-type plasminogen activator](https://tcmsp-e.com/target.php?qt=346) | PLAU |
| [apigenin](https://tcmsp-e.com/molecule.php?qn=8) | [Matrix metalloproteinase-9](https://tcmsp-e.com/target.php?qt=4334) | MMP9 |
| [apigenin](https://tcmsp-e.com/molecule.php?qn=8) | [Retinoblastoma-associated protein](https://tcmsp-e.com/target.php?qt=2915) | RB1 |
| [apigenin](https://tcmsp-e.com/molecule.php?qn=8) | [Cell division protein kinase 4](https://tcmsp-e.com/target.php?qt=573) | CDK4 |
| [apigenin](https://tcmsp-e.com/molecule.php?qn=8) | [Tumor necrosis factor](https://tcmsp-e.com/target.php?qt=265) | TNF |
| [apigenin](https://tcmsp-e.com/molecule.php?qn=8) | [Transcription factor AP-1](https://tcmsp-e.com/target.php?qt=414) | JUN |
| [apigenin](https://tcmsp-e.com/molecule.php?qn=8) | [Cell division protein kinase 6](https://tcmsp-e.com/target.php?qt=571) | CDK6 |
| [apigenin](https://tcmsp-e.com/molecule.php?qn=8) | [Eukaryotic translation elongation factor 1 epsilon-1](https://tcmsp-e.com/target.php?qt=4195) | EEF1E1 |
| [apigenin](https://tcmsp-e.com/molecule.php?qn=8) | [Activator of 90 kDa heat shock protein ATPase homolog 1](https://tcmsp-e.com/target.php?qt=4007) | AHSA1 |
| [apigenin](https://tcmsp-e.com/molecule.php?qn=8) | [Caspase-3](https://tcmsp-e.com/target.php?qt=4087) | CASP3 |
| [apigenin](https://tcmsp-e.com/molecule.php?qn=8) | [Cellular tumor antigen p53](https://tcmsp-e.com/target.php?qt=646) | TP53 |
| [apigenin](https://tcmsp-e.com/molecule.php?qn=8) | [NF-kappa-B inhibitor alpha](https://tcmsp-e.com/target.php?qt=4394) | NFKBIA |
| [apigenin](https://tcmsp-e.com/molecule.php?qn=8) | [Ornithine decarboxylase](https://tcmsp-e.com/target.php?qt=153) | ODC1 |
| [apigenin](https://tcmsp-e.com/molecule.php?qn=8) | [E3 ubiquitin-protein ligase Mdm2](https://tcmsp-e.com/target.php?qt=4179) | MDM2 |
| [apigenin](https://tcmsp-e.com/molecule.php?qn=8) | [Bcl2 antagonist of cell death](https://tcmsp-e.com/target.php?qt=4050) | BAD |
| [apigenin](https://tcmsp-e.com/molecule.php?qn=8) | [Interstitial collagenase](https://tcmsp-e.com/target.php?qt=353) | MMP1 |
| [apigenin](https://tcmsp-e.com/molecule.php?qn=8) | [Hypoxia-inducible factor 1-alpha](https://tcmsp-e.com/target.php?qt=4263) | HIF1A |
| [apigenin](https://tcmsp-e.com/molecule.php?qn=8) | [Insulin-like growth factor 1 receptor](https://tcmsp-e.com/target.php?qt=4274) | IGF1R |
| [apigenin](https://tcmsp-e.com/molecule.php?qn=8) | [Protein CBFA2T1](https://tcmsp-e.com/target.php?qt=4464) | RUNX1T1 |
| [apigenin](https://tcmsp-e.com/molecule.php?qn=8) | [Cell division control protein 2 homolog](https://tcmsp-e.com/target.php?qt=431) | CDK1 |
| [apigenin](https://tcmsp-e.com/molecule.php?qn=8) | [Acetyl-CoA carboxylase 1](https://tcmsp-e.com/target.php?qt=231) | ACACA |
| [apigenin](https://tcmsp-e.com/molecule.php?qn=8) | [Heme oxygenase 1](https://tcmsp-e.com/target.php?qt=2132) | HMOX1 |
| [apigenin](https://tcmsp-e.com/molecule.php?qn=8) | [Intercellular adhesion molecule 1](https://tcmsp-e.com/target.php?qt=4287) | ICAM1 |
| [apigenin](https://tcmsp-e.com/molecule.php?qn=8) | [Induced myeloid leukemia cell differentiation protein Mcl-1](https://tcmsp-e.com/target.php?qt=4269) | MCL1 |
| [apigenin](https://tcmsp-e.com/molecule.php?qn=8) | [G1/S-specific cyclin-D2](https://tcmsp-e.com/target.php?qt=4215) | CCND2 |
| [apigenin](https://tcmsp-e.com/molecule.php?qn=8) | [Interleukin-2](https://tcmsp-e.com/target.php?qt=3978) | IL2 |
| [apigenin](https://tcmsp-e.com/molecule.php?qn=8) | [G2/mitotic-specific cyclin-B1](https://tcmsp-e.com/target.php?qt=4219) | CCNB1 |
| [apigenin](https://tcmsp-e.com/molecule.php?qn=8) | [Plasminogen activator inhibitor 1](https://tcmsp-e.com/target.php?qt=4429) | SERPINE1 |
| [apigenin](https://tcmsp-e.com/molecule.php?qn=8) | [Interferon gamma](https://tcmsp-e.com/target.php?qt=365) | IFNG |
| [apigenin](https://tcmsp-e.com/molecule.php?qn=8) | [Interleukin-4](https://tcmsp-e.com/target.php?qt=4301) | IL4 |
| [apigenin](https://tcmsp-e.com/molecule.php?qn=8) | [NF-kappa-B essential modulator](https://tcmsp-e.com/target.php?qt=4393) | IKBKG |
| [apigenin](https://tcmsp-e.com/molecule.php?qn=8) | [Cytochrome P450 19A1](https://tcmsp-e.com/target.php?qt=4148) | CYP19A1 |
| [apigenin](https://tcmsp-e.com/molecule.php?qn=8) | [Baculoviral IAP repeat-containing protein 4](https://tcmsp-e.com/target.php?qt=4043) | XIAP |
| [apigenin](https://tcmsp-e.com/molecule.php?qn=8) | [26S proteasome non-ATPase regulatory subunit 3](https://tcmsp-e.com/target.php?qt=3993) | PSMD3 |
| [apigenin](https://tcmsp-e.com/molecule.php?qn=8) | [Solute carrier family 2, facilitated glucose transporter member 4](https://tcmsp-e.com/target.php?qt=4535) | SLC2A4 |
| [apigenin](https://tcmsp-e.com/molecule.php?qn=8) | [Insulin receptor](https://tcmsp-e.com/target.php?qt=11) | INSR |
| [apigenin](https://tcmsp-e.com/molecule.php?qn=8) | [CD40 ligand](https://tcmsp-e.com/target.php?qt=4107) | CD40LG |
| [apigenin](https://tcmsp-e.com/molecule.php?qn=8) | [Cytochrome c](https://tcmsp-e.com/target.php?qt=3611) | CYCS |
| [apigenin](https://tcmsp-e.com/molecule.php?qn=8) | [CASP8 and FADD-like apoptosis regulator](https://tcmsp-e.com/target.php?qt=4083) | CFLAR |
| [apigenin](https://tcmsp-e.com/molecule.php?qn=8) | [Alpha- and gamma-adaptin-binding protein p34](https://tcmsp-e.com/target.php?qt=4023) | AAGAB |
| [apigenin](https://tcmsp-e.com/molecule.php?qn=8) | [Insulin](https://tcmsp-e.com/target.php?qt=2882) | INS |
| [apigenin](https://tcmsp-e.com/molecule.php?qn=8) | [Low affinity immunoglobulin epsilon Fc receptor](https://tcmsp-e.com/target.php?qt=4326) | FCER2 |
| [apigenin](https://tcmsp-e.com/molecule.php?qn=8) | [Interleukin-13](https://tcmsp-e.com/target.php?qt=4293) | IL13 |
| [apigenin](https://tcmsp-e.com/molecule.php?qn=8) | [High affinity immunoglobulin epsilon receptor subunit beta](https://tcmsp-e.com/target.php?qt=784) | MS4A2 |
| [apigenin](https://tcmsp-e.com/molecule.php?qn=8) | [Intestinal-type alkaline phosphatase](https://tcmsp-e.com/target.php?qt=4304) | ALPI |
| [apigenin](https://tcmsp-e.com/molecule.php?qn=8) | [Proteasome activator complex subunit 3](https://tcmsp-e.com/target.php?qt=4460) | PSME3 |
| [apigenin](https://tcmsp-e.com/molecule.php?qn=8) | [Glucose-6-phosphatase](https://tcmsp-e.com/target.php?qt=4229) | G6PC |
| [apigenin](https://tcmsp-e.com/molecule.php?qn=8) | [Adenomatous polyposis coli protein](https://tcmsp-e.com/target.php?qt=4012) | APC |
| [apigenin](https://tcmsp-e.com/molecule.php?qn=8) | [Transient receptor potential cation channel subfamily M member 2](https://tcmsp-e.com/target.php?qt=4573) | TRPM2 |
| [apigenin](https://tcmsp-e.com/molecule.php?qn=8) | [Aldo-keto reductase family 1 member C3](https://tcmsp-e.com/target.php?qt=222) | AKR1C3 |
| [apigenin](https://tcmsp-e.com/molecule.php?qn=8) | [Sodium/iodide cotransporter](https://tcmsp-e.com/target.php?qt=4527) | SLC5A5 |
| [apigenin](https://tcmsp-e.com/molecule.php?qn=8) | [Dolichyl-phosphate beta-glucosyltransferase](https://tcmsp-e.com/target.php?qt=4173) | ALG5 |
| [(1R)-2,3,4,9-tetrahydro-1H-$b-carboline-1-carboxylic acid](https://tcmsp-e.com/molecule.php?qn=8375) | [Prostaglandin G/H synthase 1](https://tcmsp-e.com/target.php?qt=6) | PTGS1 |
| [(1R)-2,3,4,9-tetrahydro-1H-$b-carboline-1-carboxylic acid](https://tcmsp-e.com/molecule.php?qn=8375) | [Beta-1 adrenergic receptor](https://tcmsp-e.com/target.php?qt=63) | ADRB1 |
| [(1R)-2,3,4,9-tetrahydro-1H-$b-carboline-1-carboxylic acid](https://tcmsp-e.com/molecule.php?qn=8375) | [Sodium channel protein type 5 subunit alpha](https://tcmsp-e.com/target.php?qt=70) | SCN5A |
| [(1R)-2,3,4,9-tetrahydro-1H-$b-carboline-1-carboxylic acid](https://tcmsp-e.com/molecule.php?qn=8375) | [Peroxisome proliferator activated receptor gamma](https://tcmsp-e.com/target.php?qt=78) | PPARG |
| [(1R)-2,3,4,9-tetrahydro-1H-$b-carboline-1-carboxylic acid](https://tcmsp-e.com/molecule.php?qn=8375) | [Prostaglandin G/H synthase 2](https://tcmsp-e.com/target.php?qt=94) | PTGS2 |
| [(1R)-2,3,4,9-tetrahydro-1H-$b-carboline-1-carboxylic acid](https://tcmsp-e.com/molecule.php?qn=8375) | [Nitric-oxide synthase, endothelial](https://tcmsp-e.com/target.php?qt=95) | NOS3 |
| [(1R)-2,3,4,9-tetrahydro-1H-$b-carboline-1-carboxylic acid](https://tcmsp-e.com/molecule.php?qn=8375) | [CGMP-inhibited 3',5'-cyclic phosphodiesterase A](https://tcmsp-e.com/target.php?qt=172) | PDE3A |
| [(1R)-2,3,4,9-tetrahydro-1H-$b-carboline-1-carboxylic acid](https://tcmsp-e.com/molecule.php?qn=8375) | [Gamma-aminobutyric acid receptor subunit alpha-1](https://tcmsp-e.com/target.php?qt=309) | GABRA1 |
| [(1R)-2,3,4,9-tetrahydro-1H-$b-carboline-1-carboxylic acid](https://tcmsp-e.com/molecule.php?qn=8375) | [Heat shock protein HSP 90](https://tcmsp-e.com/target.php?qt=444) | HSP90AA1 |
| [(1R)-2,3,4,9-tetrahydro-1H-$b-carboline-1-carboxylic acid](https://tcmsp-e.com/molecule.php?qn=8375) | [Beta-lactamase](https://tcmsp-e.com/target.php?qt=499) | LACTBL1 |
| [(1R)-2,3,4,9-tetrahydro-1H-$b-carboline-1-carboxylic acid](https://tcmsp-e.com/molecule.php?qn=8375) | [Leukotriene A-4 hydrolase](https://tcmsp-e.com/target.php?qt=521) | LTA4H |
| [(1R)-2,3,4,9-tetrahydro-1H-$b-carboline-1-carboxylic acid](https://tcmsp-e.com/molecule.php?qn=8375) | [Amine oxidase [flavin-containing] B](https://tcmsp-e.com/target.php?qt=565) | MAOB |
| [(1R)-2,3,4,9-tetrahydro-1H-$b-carboline-1-carboxylic acid](https://tcmsp-e.com/molecule.php?qn=8375) | [cAMP-dependent protein kinase inhibitor alpha](https://tcmsp-e.com/target.php?qt=3586) | PKIA |
| [tangshenoside I_qt](https://tcmsp-e.com/molecule.php?qn=8380) | [Nitric oxide synthase, inducible](https://tcmsp-e.com/target.php?qt=3) | NOS2 |
| [tangshenoside I_qt](https://tcmsp-e.com/molecule.php?qn=8380) | [Coagulation factor Xa](https://tcmsp-e.com/target.php?qt=79) | F7 |
| [tangshenoside I_qt](https://tcmsp-e.com/molecule.php?qn=8380) | [Prostaglandin G/H synthase 2](https://tcmsp-e.com/target.php?qt=94) | PTGS2 |
| [7-(beta-Xylosyl)cephalomannine_qt](https://tcmsp-e.com/molecule.php?qn=8393) | [Tubulin beta-1 chain](https://tcmsp-e.com/target.php?qt=3444) | TUBB1 |
| [Codonopsine](https://tcmsp-e.com/molecule.php?qn=8395) | [Dopamine D1 receptor](https://tcmsp-e.com/target.php?qt=7) | DRD1 |
| [Codonopsine](https://tcmsp-e.com/molecule.php?qn=8395) | [Muscarinic acetylcholine receptor M3](https://tcmsp-e.com/target.php?qt=16) | CHRM3 |
| [Codonopsine](https://tcmsp-e.com/molecule.php?qn=8395) | [Muscarinic acetylcholine receptor M1](https://tcmsp-e.com/target.php?qt=38) | CHRM1 |
| [Codonopsine](https://tcmsp-e.com/molecule.php?qn=8395) | [Androgen receptor](https://tcmsp-e.com/target.php?qt=48) | AR |
| [Codonopsine](https://tcmsp-e.com/molecule.php?qn=8395) | [Beta-1 adrenergic receptor](https://tcmsp-e.com/target.php?qt=63) | ADRB1 |
| [Codonopsine](https://tcmsp-e.com/molecule.php?qn=8395) | [Sodium channel protein type 5 subunit alpha](https://tcmsp-e.com/target.php?qt=70) | SCN5A |
| [Codonopsine](https://tcmsp-e.com/molecule.php?qn=8395) | [Muscarinic acetylcholine receptor M5](https://tcmsp-e.com/target.php?qt=87) | CHRM5 |
| [Codonopsine](https://tcmsp-e.com/molecule.php?qn=8395) | [Prostaglandin G/H synthase 2](https://tcmsp-e.com/target.php?qt=94) | PTGS2 |
| [Codonopsine](https://tcmsp-e.com/molecule.php?qn=8395) | [Alpha-2A adrenergic receptor](https://tcmsp-e.com/target.php?qt=105) | ADRA2A |
| [Codonopsine](https://tcmsp-e.com/molecule.php?qn=8395) | [Alpha-2C adrenergic receptor](https://tcmsp-e.com/target.php?qt=126) | ADRA2C |
| [Codonopsine](https://tcmsp-e.com/molecule.php?qn=8395) | [Delta-type opioid receptor](https://tcmsp-e.com/target.php?qt=163) | OPRD1 |
| [Codonopsine](https://tcmsp-e.com/molecule.php?qn=8395) | [CGMP-inhibited 3',5'-cyclic phosphodiesterase A](https://tcmsp-e.com/target.php?qt=172) | PDE3A |
| [Codonopsine](https://tcmsp-e.com/molecule.php?qn=8395) | [Sodium-dependent noradrenaline transporter](https://tcmsp-e.com/target.php?qt=186) | SLC6A2 |
| [Codonopsine](https://tcmsp-e.com/molecule.php?qn=8395) | [Alpha-1A adrenergic receptor](https://tcmsp-e.com/target.php?qt=191) | ADRA1D |
| [Codonopsine](https://tcmsp-e.com/molecule.php?qn=8395) | [Alpha-1B adrenergic receptor](https://tcmsp-e.com/target.php?qt=216) | ADRA1B |
| [Codonopsine](https://tcmsp-e.com/molecule.php?qn=8395) | [Sodium-dependent dopamine transporter](https://tcmsp-e.com/target.php?qt=239) | SLC6A3 |
| [Codonopsine](https://tcmsp-e.com/molecule.php?qn=8395) | [Beta-2 adrenergic receptor](https://tcmsp-e.com/target.php?qt=261) | ADRB2 |
| [Codonopsine](https://tcmsp-e.com/molecule.php?qn=8395) | [Sodium-dependent serotonin transporter](https://tcmsp-e.com/target.php?qt=290) | SLC6A4 |
| [Codonopsine](https://tcmsp-e.com/molecule.php?qn=8395) | [Gamma-aminobutyric acid receptor subunit alpha-1](https://tcmsp-e.com/target.php?qt=309) | GABRA1 |
| [Coelogin](https://tcmsp-e.com/molecule.php?qn=8396) | [Nitric oxide synthase, inducible](https://tcmsp-e.com/target.php?qt=3) | NOS2 |
| [Coelogin](https://tcmsp-e.com/molecule.php?qn=8396) | [Prostaglandin G/H synthase 1](https://tcmsp-e.com/target.php?qt=6) | PTGS1 |
| [Coelogin](https://tcmsp-e.com/molecule.php?qn=8396) | [Androgen receptor](https://tcmsp-e.com/target.php?qt=48) | AR |
| [Coelogin](https://tcmsp-e.com/molecule.php?qn=8396) | [Sodium channel protein type 5 subunit alpha](https://tcmsp-e.com/target.php?qt=70) | SCN5A |
| [Coelogin](https://tcmsp-e.com/molecule.php?qn=8396) | [Prostaglandin G/H synthase 2](https://tcmsp-e.com/target.php?qt=94) | PTGS2 |
| [Coelogin](https://tcmsp-e.com/molecule.php?qn=8396) | [Retinoic acid receptor RXR-alpha](https://tcmsp-e.com/target.php?qt=158) | RXRA |
| [Coelogin](https://tcmsp-e.com/molecule.php?qn=8396) | [Acetylcholinesterase](https://tcmsp-e.com/target.php?qt=165) | ACHE |
| [Coelogin](https://tcmsp-e.com/molecule.php?qn=8396) | [DNA topoisomerase II](https://tcmsp-e.com/target.php?qt=287) | TOP2B |
| [Coelogin](https://tcmsp-e.com/molecule.php?qn=8396) | [Heat shock protein HSP 90](https://tcmsp-e.com/target.php?qt=444) | HSP90AA1 |
| [Daturilin](https://tcmsp-e.com/molecule.php?qn=8397) | [Glucocorticoid receptor](https://tcmsp-e.com/target.php?qt=308) | NR3C1 |
| [glycitein](https://tcmsp-e.com/molecule.php?qn=8400) | [Prostaglandin G/H synthase 1](https://tcmsp-e.com/target.php?qt=6) | PTGS1 |
| [glycitein](https://tcmsp-e.com/molecule.php?qn=8400) | [Estrogen receptor](https://tcmsp-e.com/target.php?qt=46) | ESR1 |
| [glycitein](https://tcmsp-e.com/molecule.php?qn=8400) | [Androgen receptor](https://tcmsp-e.com/target.php?qt=48) | AR |
| [glycitein](https://tcmsp-e.com/molecule.php?qn=8400) | [Peroxisome proliferator activated receptor gamma](https://tcmsp-e.com/target.php?qt=78) | PPARG |
| [glycitein](https://tcmsp-e.com/molecule.php?qn=8400) | [Prostaglandin G/H synthase 2](https://tcmsp-e.com/target.php?qt=94) | PTGS2 |
| [glycitein](https://tcmsp-e.com/molecule.php?qn=8400) | [Retinoic acid receptor RXR-alpha](https://tcmsp-e.com/target.php?qt=158) | RXRA |
| [glycitein](https://tcmsp-e.com/molecule.php?qn=8400) | [CGMP-inhibited 3',5'-cyclic phosphodiesterase A](https://tcmsp-e.com/target.php?qt=172) | PDE3A |
| [glycitein](https://tcmsp-e.com/molecule.php?qn=8400) | [Estrogen receptor beta](https://tcmsp-e.com/target.php?qt=307) | ESR2 |
| [glycitein](https://tcmsp-e.com/molecule.php?qn=8400) | [Mitogen-activated protein kinase 14](https://tcmsp-e.com/target.php?qt=402) | MAPK14 |
| [glycitein](https://tcmsp-e.com/molecule.php?qn=8400) | [Glycogen synthase kinase-3 beta](https://tcmsp-e.com/target.php?qt=422) | GSK3B |
| [glycitein](https://tcmsp-e.com/molecule.php?qn=8400) | [Heat shock protein HSP 90](https://tcmsp-e.com/target.php?qt=444) | HSP90AA1 |
| [glycitein](https://tcmsp-e.com/molecule.php?qn=8400) | [Cell division protein kinase 2](https://tcmsp-e.com/target.php?qt=482) | CDK2 |
| [glycitein](https://tcmsp-e.com/molecule.php?qn=8400) | [Serine/threonine-protein kinase Chk1](https://tcmsp-e.com/target.php?qt=647) | CHEK1 |
| [glycitein](https://tcmsp-e.com/molecule.php?qn=8400) | [Trypsin-1](https://tcmsp-e.com/target.php?qt=2928) | PRSS1 |
| [glycitein](https://tcmsp-e.com/molecule.php?qn=8400) | [Cyclin-A2](https://tcmsp-e.com/target.php?qt=3025) | CCNA2 |
| [glycitein](https://tcmsp-e.com/molecule.php?qn=8400) | [Calmodulin](https://tcmsp-e.com/target.php?qt=3907) | CALM3 |
| [glycitein](https://tcmsp-e.com/molecule.php?qn=8400) | [Nuclear receptor coactivator 1](https://tcmsp-e.com/target.php?qt=3279) | NCOA1 |
| [glycitein](https://tcmsp-e.com/molecule.php?qn=8400) | [Nitric oxide synthase, inducible](https://tcmsp-e.com/target.php?qt=3) | NOS2 |
| [glycitein](https://tcmsp-e.com/molecule.php?qn=8400) | [Amyloid beta A4 protein](https://tcmsp-e.com/target.php?qt=648) | APP |
| [glycitein](https://tcmsp-e.com/molecule.php?qn=8400) | [Collagenase 3](https://tcmsp-e.com/target.php?qt=377) | MMP13 |
| [glycitein](https://tcmsp-e.com/molecule.php?qn=8400) | [Neutrophil collagenase](https://tcmsp-e.com/target.php?qt=468) | MMP8 |
| [(8S,9S,10R,13R,14S,17R)-17-[(E,2R,5S)-5-ethyl-6-methylhept-3-en-2-yl]-10,13-dimethyl-1,2,4,7,8,9,11,12,14,15,16,17-dodecahydrocyclopenta[a]phenanthren-3-one](https://tcmsp-e.com/molecule.php?qn=8407) | [Progesterone receptor](https://tcmsp-e.com/target.php?qt=209) | PGR |
| [(8S,9S,10R,13R,14S,17R)-17-[(E,2R,5S)-5-ethyl-6-methylhept-3-en-2-yl]-10,13-dimethyl-1,2,4,7,8,9,11,12,14,15,16,17-dodecahydrocyclopenta[a]phenanthren-3-one](https://tcmsp-e.com/molecule.php?qn=8407) | [Mineralocorticoid receptor](https://tcmsp-e.com/target.php?qt=252) | NR3C2 |
| [11-Hydroxyrankinidine](https://tcmsp-e.com/molecule.php?qn=8411) | [Estrogen receptor](https://tcmsp-e.com/target.php?qt=46) | ESR1 |
| [11-Hydroxyrankinidine](https://tcmsp-e.com/molecule.php?qn=8411) | [Sodium channel protein type 5 subunit alpha](https://tcmsp-e.com/target.php?qt=70) | SCN5A |
| [11-Hydroxyrankinidine](https://tcmsp-e.com/molecule.php?qn=8411) | [Mu-type opioid receptor](https://tcmsp-e.com/target.php?qt=299) | OPRM1 |
| [11-Hydroxyrankinidine](https://tcmsp-e.com/molecule.php?qn=8411) | [Cell division protein kinase 2](https://tcmsp-e.com/target.php?qt=482) | CDK2 |
| [Furanodiene](https://tcmsp-e.com/molecule.php?qn=899) | [Muscarinic acetylcholine receptor M3](https://tcmsp-e.com/target.php?qt=16) | CHRM3 |
| [Furanodiene](https://tcmsp-e.com/molecule.php?qn=899) | [Thrombin](https://tcmsp-e.com/target.php?qt=17) | SERPIND1 |
| [Furanodiene](https://tcmsp-e.com/molecule.php?qn=899) | [Muscarinic acetylcholine receptor M1](https://tcmsp-e.com/target.php?qt=38) | CHRM1 |
| [Furanodiene](https://tcmsp-e.com/molecule.php?qn=899) | [Prostaglandin G/H synthase 2](https://tcmsp-e.com/target.php?qt=94) | PTGS2 |
| [Furanodiene](https://tcmsp-e.com/molecule.php?qn=899) | [Nitric-oxide synthase, endothelial](https://tcmsp-e.com/target.php?qt=95) | NOS3 |
| [Furanodiene](https://tcmsp-e.com/molecule.php?qn=899) | [Sodium-dependent dopamine transporter](https://tcmsp-e.com/target.php?qt=239) | SLC6A3 |
| [Furanodiene](https://tcmsp-e.com/molecule.php?qn=899) | [Beta-2 adrenergic receptor](https://tcmsp-e.com/target.php?qt=261) | ADRB2 |
| [Furanodiene](https://tcmsp-e.com/molecule.php?qn=899) | [Sodium-dependent serotonin transporter](https://tcmsp-e.com/target.php?qt=290) | SLC6A4 |
| [Furanodiene](https://tcmsp-e.com/molecule.php?qn=899) | [Dipeptidyl peptidase IV](https://tcmsp-e.com/target.php?qt=332) | DPP4 |
| [delta 7-stigmastenol](https://tcmsp-e.com/molecule.php?qn=95) | [Progesterone receptor](https://tcmsp-e.com/target.php?qt=209) | PGR |
