# Supplementary material for: The mechanistic study of codonopsis pilosula on laryngeal squamous cell carcinoma based on network pharmacology and experimental validation
Source: Front Pharmacol. 2025 Apr 25;16:1542116. doi: 10.3389/fphar.2025.1542116 (PMC12061682; doi:10.3389/fphar.2025.1542116)
Supplement: Supplementary file 1 [file DataSheet1.zip › Supplementary Material/Supplementary_Table S3.docx]

**Supplementary Table S3.** Effective compounds and targets of Codonopsis pilosula.

| **Chemical compound** | **Target genes** | **Druglikeness Grading** |
| --- | --- | --- |
| O-(O-Methoxyphenoxy)Phenol | ALB | good |
| O-(O-Methoxyphenoxy)Phenol | AR | good |
| O-(O-Methoxyphenoxy)Phenol | ESR1 | good |
| O-(O-Methoxyphenoxy)Phenol | ESR2 | good |
| O-(O-Methoxyphenoxy)Phenol | PGR | good |
| Syringaldehyde | AR | good |
| Syringaldehyde | ESR1 | good |
| Syringaldehyde | ESR2 | good |
| Syringaldehyde | GLO1 | good |
| Syringaldehyde | PGR | good |
| Syringaldehyde | SHBG | good |
| Lauric Acid | ABAT | moderate |
| Lauric Acid | ABL1 | moderate |
| Lauric Acid | ACADSB | moderate |
| Lauric Acid | ACOX1 | moderate |
| Lauric Acid | ACSL3 | moderate |
| Lauric Acid | ACSL4 | moderate |
| Lauric Acid | ADH5 | moderate |
| Lauric Acid | ALDH5A1 | moderate |
| Lauric Acid | ARF1 | moderate |
| Lauric Acid | ARF6 | moderate |
| Lauric Acid | C8G | moderate |
| Lauric Acid | CALM1 | moderate |
| Lauric Acid | CNR1 | moderate |
| Lauric Acid | CNR2 | moderate |
| Lauric Acid | CYP2C8 | moderate |
| Lauric Acid | ECI2 | moderate |
| Lauric Acid | ELOVL4 | moderate |
| Lauric Acid | FADS1 | moderate |
| Lauric Acid | FADS2 | moderate |
| Lauric Acid | FFAR1 | moderate |
| Lauric Acid | FKBP1A | moderate |
| Lauric Acid | FURIN | moderate |
| Lauric Acid | GLTP | moderate |
| Lauric Acid | GM2A | moderate |
| Lauric Acid | GUCA1A | moderate |
| Lauric Acid | HAO1 | moderate |
| Lauric Acid | HDAC2 | moderate |
| Lauric Acid | HDAC9 | moderate |
| Lauric Acid | HNF4A | moderate |
| Lauric Acid | HNF4G | moderate |
| Lauric Acid | INS | moderate |
| Lauric Acid | LALBA | moderate |
| Lauric Acid | LIP3 | moderate |
| Lauric Acid | LTB4R | moderate |
| Lauric Acid | LTB4R2 | moderate |
| Lauric Acid | LTF | moderate |
| Lauric Acid | LY96 | moderate |
| Lauric Acid | NCOA1 | moderate |
| Lauric Acid | NR1H4 | moderate |
| Lauric Acid | OGDH | moderate |
| Lauric Acid | PAEP | moderate |
| Lauric Acid | PKIA | moderate |
| Lauric Acid | PLA2G1B | moderate |
| Lauric Acid | PLA2G2A | moderate |
| Lauric Acid | PLA2G2D | moderate |
| Lauric Acid | PLA2G2E | moderate |
| Lauric Acid | PMP2 | moderate |
| Lauric Acid | PPARA | moderate |
| Lauric Acid | PPARD | moderate |
| Lauric Acid | PPARG | moderate |
| Lauric Acid | PPA_RS05235 | moderate |
| Lauric Acid | PPP3CA | moderate |
| Lauric Acid | PPP3R1 | moderate |
| Lauric Acid | PPT1 | moderate |
| Lauric Acid | PRKACA | moderate |
| Lauric Acid | PTGS1 | moderate |
| Lauric Acid | PTGS2 | moderate |
| Lauric Acid | PVR | moderate |
| Lauric Acid | RCVRN | moderate |
| Lauric Acid | RHO | moderate |
| Lauric Acid | RXRA | moderate |
| Lauric Acid | RXRB | moderate |
| Lauric Acid | RXRG | moderate |
| Lauric Acid | S100B | moderate |
| Lauric Acid | SCN10A | moderate |
| Lauric Acid | SCN11A | moderate |
| Lauric Acid | SCN1A | moderate |
| Lauric Acid | SCN1B | moderate |
| Lauric Acid | SCN2A | moderate |
| Lauric Acid | SCN2B | moderate |
| Lauric Acid | SCN3A | moderate |
| Lauric Acid | SCN3B | moderate |
| Lauric Acid | SCN4A | moderate |
| Lauric Acid | SCN4B | moderate |
| Lauric Acid | SCN5A | moderate |
| Lauric Acid | SCN7A | moderate |
| Lauric Acid | SCN8A | moderate |
| Lauric Acid | SCN9A | moderate |
| Lauric Acid | SEC14L2 | moderate |
| Lauric Acid | SLC8A1 | moderate |
| Lauric Acid | SREBF1 | moderate |
| Lauric Acid | TLR4 | moderate |
| Lauric Acid | TM1468 | moderate |
| Lauric Acid | TRAPPC3 | moderate |
| Lauric Acid | TRPV1 | moderate |
| Lauric Acid | VLDLR | moderate |
| Caprylic Acid | ABAT | moderate |
| Caprylic Acid | ABL1 | moderate |
| Caprylic Acid | ACADSB | moderate |
| Caprylic Acid | ACOX1 | moderate |
| Caprylic Acid | ACSL3 | moderate |
| Caprylic Acid | ACSL4 | moderate |
| Caprylic Acid | ADH5 | moderate |
| Caprylic Acid | ALDH5A1 | moderate |
| Caprylic Acid | ARF1 | moderate |
| Caprylic Acid | ARF6 | moderate |
| Caprylic Acid | C8G | moderate |
| Caprylic Acid | CALM1 | moderate |
| Caprylic Acid | CNR1 | moderate |
| Caprylic Acid | CNR2 | moderate |
| Caprylic Acid | CYP2C8 | moderate |
| Caprylic Acid | ECI2 | moderate |
| Caprylic Acid | ELOVL4 | moderate |
| Caprylic Acid | FADS1 | moderate |
| Caprylic Acid | FADS2 | moderate |
| Caprylic Acid | FFAR1 | moderate |
| Caprylic Acid | FKBP1A | moderate |
| Caprylic Acid | FURIN | moderate |
| Caprylic Acid | GLTP | moderate |
| Caprylic Acid | GM2A | moderate |
| Caprylic Acid | GUCA1A | moderate |
| Caprylic Acid | HAO1 | moderate |
| Caprylic Acid | HDAC2 | moderate |
| Caprylic Acid | HDAC9 | moderate |
| Caprylic Acid | HNF4A | moderate |
| Caprylic Acid | HNF4G | moderate |
| Caprylic Acid | INS | moderate |
| Caprylic Acid | LALBA | moderate |
| Caprylic Acid | LIP3 | moderate |
| Caprylic Acid | LTB4R | moderate |
| Caprylic Acid | LTB4R2 | moderate |
| Caprylic Acid | LTF | moderate |
| Caprylic Acid | LY96 | moderate |
| Caprylic Acid | NCOA1 | moderate |
| Caprylic Acid | NR1H4 | moderate |
| Caprylic Acid | OGDH | moderate |
| Caprylic Acid | PAEP | moderate |
| Caprylic Acid | PKIA | moderate |
| Caprylic Acid | PLA2G1B | moderate |
| Caprylic Acid | PLA2G2A | moderate |
| Caprylic Acid | PLA2G2D | moderate |
| Caprylic Acid | PLA2G2E | moderate |
| Caprylic Acid | PMP2 | moderate |
| Caprylic Acid | PPARA | moderate |
| Caprylic Acid | PPARD | moderate |
| Caprylic Acid | PPARG | moderate |
| Caprylic Acid | PPA_RS05235 | moderate |
| Caprylic Acid | PPP3CA | moderate |
| Caprylic Acid | PPP3R1 | moderate |
| Caprylic Acid | PPT1 | moderate |
| Caprylic Acid | PRKACA | moderate |
| Caprylic Acid | PTGS1 | moderate |
| Caprylic Acid | PTGS2 | moderate |
| Caprylic Acid | PVR | moderate |
| Caprylic Acid | RCVRN | moderate |
| Caprylic Acid | RHO | moderate |
| Caprylic Acid | RXRA | moderate |
| Caprylic Acid | RXRB | moderate |
| Caprylic Acid | RXRG | moderate |
| Caprylic Acid | S100B | moderate |
| Caprylic Acid | SCN10A | moderate |
| Caprylic Acid | SCN11A | moderate |
| Caprylic Acid | SCN1A | moderate |
| Caprylic Acid | SCN1B | moderate |
| Caprylic Acid | SCN2A | moderate |
| Caprylic Acid | SCN2B | moderate |
| Caprylic Acid | SCN3A | moderate |
| Caprylic Acid | SCN3B | moderate |
| Caprylic Acid | SCN4A | moderate |
| Caprylic Acid | SCN4B | moderate |
| Caprylic Acid | SCN5A | moderate |
| Caprylic Acid | SCN7A | moderate |
| Caprylic Acid | SCN8A | moderate |
| Caprylic Acid | SCN9A | moderate |
| Caprylic Acid | SEC14L2 | moderate |
| Caprylic Acid | SLC8A1 | moderate |
| Caprylic Acid | SREBF1 | moderate |
| Caprylic Acid | TLR4 | moderate |
| Caprylic Acid | TM1468 | moderate |
| Caprylic Acid | TRAPPC3 | moderate |
| Caprylic Acid | TRPV1 | moderate |
| Caprylic Acid | VLDLR | moderate |
| Atractylenolide III | AADACL2 | moderate |
| Atractylenolide III | AKR1C1 | moderate |
| Atractylenolide III | AKR1C2 | moderate |
| Atractylenolide III | AR | moderate |
| Atractylenolide III | ESR1 | moderate |
| Atractylenolide III | ESR2 | moderate |
| Atractylenolide III | GABRA1 | moderate |
| Atractylenolide III | GABRA2 | moderate |
| Atractylenolide III | GABRA3 | moderate |
| Atractylenolide III | GABRA4 | moderate |
| Atractylenolide III | GABRA5 | moderate |
| Atractylenolide III | GABRA6 | moderate |
| Atractylenolide III | GABRB1 | moderate |
| Atractylenolide III | GABRB2 | moderate |
| Atractylenolide III | GABRB3 | moderate |
| Atractylenolide III | GABRD | moderate |
| Atractylenolide III | GABRE | moderate |
| Atractylenolide III | GABRG1 | moderate |
| Atractylenolide III | GABRG2 | moderate |
| Atractylenolide III | GABRG3 | moderate |
| Atractylenolide III | GABRP | moderate |
| Atractylenolide III | GABRQ | moderate |
| Atractylenolide III | GRIN1 | moderate |
| Atractylenolide III | GRIN2A | moderate |
| Atractylenolide III | GRIN2B | moderate |
| Atractylenolide III | GRIN2C | moderate |
| Atractylenolide III | GRIN2D | moderate |
| Atractylenolide III | GRIN3A | moderate |
| Atractylenolide III | GRIN3B | moderate |
| Atractylenolide III | HSD11B2 | moderate |
| Atractylenolide III | HSD17B1 | moderate |
| Atractylenolide III | IL1B | moderate |
| Atractylenolide III | IL6 | moderate |
| Atractylenolide III | NCOA2 | moderate |
| Atractylenolide III | NFKB1 | moderate |
| Atractylenolide III | NFKB2 | moderate |
| Atractylenolide III | NPPB | moderate |
| Atractylenolide III | NR1I2 | moderate |
| Atractylenolide III | NR1I3 | moderate |
| Atractylenolide III | NR3C2 | moderate |
| Atractylenolide III | PGR | moderate |
| Atractylenolide III | PPARA | moderate |
| Atractylenolide III | SIGMAR1 | moderate |
| Atractylenolide III | SULT2A1 | moderate |
| Atractylenolide III | SULT2B1 | moderate |
| Atractylenolide III | TNF | moderate |
| Caproic Acid | ABL1 | moderate |
| Caproic Acid | ACSL3 | moderate |
| Caproic Acid | ACSL4 | moderate |
| Caproic Acid | ADH5 | moderate |
| Caproic Acid | ARF1 | moderate |
| Caproic Acid | ARF6 | moderate |
| Caproic Acid | C8G | moderate |
| Caproic Acid | CALM1 | moderate |
| Caproic Acid | CYP2C8 | moderate |
| Caproic Acid | ECI2 | moderate |
| Caproic Acid | ELOVL4 | moderate |
| Caproic Acid | FADS1 | moderate |
| Caproic Acid | FADS2 | moderate |
| Caproic Acid | FFAR1 | moderate |
| Caproic Acid | FKBP1A | moderate |
| Caproic Acid | FURIN | moderate |
| Caproic Acid | GLTP | moderate |
| Caproic Acid | GM2A | moderate |
| Caproic Acid | GUCA1A | moderate |
| Caproic Acid | HNF4A | moderate |
| Caproic Acid | HNF4G | moderate |
| Caproic Acid | INS | moderate |
| Caproic Acid | LALBA | moderate |
| Caproic Acid | LIP3 | moderate |
| Caproic Acid | LTF | moderate |
| Caproic Acid | LY96 | moderate |
| Caproic Acid | NCOA1 | moderate |
| Caproic Acid | NR1H4 | moderate |
| Caproic Acid | PAEP | moderate |
| Caproic Acid | PKIA | moderate |
| Caproic Acid | PLA2G1B | moderate |
| Caproic Acid | PLA2G2A | moderate |
| Caproic Acid | PLA2G2D | moderate |
| Caproic Acid | PLA2G2E | moderate |
| Caproic Acid | PMP2 | moderate |
| Caproic Acid | PPARA | moderate |
| Caproic Acid | PPARD | moderate |
| Caproic Acid | PPARG | moderate |
| Caproic Acid | PPA_RS05235 | moderate |
| Caproic Acid | PPP3CA | moderate |
| Caproic Acid | PPP3R1 | moderate |
| Caproic Acid | PPT1 | moderate |
| Caproic Acid | PRKACA | moderate |
| Caproic Acid | PTGS1 | moderate |
| Caproic Acid | PTGS2 | moderate |
| Caproic Acid | PVR | moderate |
| Caproic Acid | RCVRN | moderate |
| Caproic Acid | RHO | moderate |
| Caproic Acid | RXRA | moderate |
| Caproic Acid | S100B | moderate |
| Caproic Acid | SEC14L2 | moderate |
| Caproic Acid | SLC8A1 | moderate |
| Caproic Acid | TLR4 | moderate |
| Caproic Acid | TM1468 | moderate |
| Caproic Acid | TRAPPC3 | moderate |
| Caproic Acid | TRPV1 | moderate |
| Caproic Acid | VLDLR | moderate |
| Enanthic Acid | ABAT | moderate |
| Enanthic Acid | ABL1 | moderate |
| Enanthic Acid | ACADSB | moderate |
| Enanthic Acid | ACOX1 | moderate |
| Enanthic Acid | ACSL3 | moderate |
| Enanthic Acid | ACSL4 | moderate |
| Enanthic Acid | ADH5 | moderate |
| Enanthic Acid | ALDH5A1 | moderate |
| Enanthic Acid | ARF1 | moderate |
| Enanthic Acid | ARF6 | moderate |
| Enanthic Acid | C8G | moderate |
| Enanthic Acid | CALM1 | moderate |
| Enanthic Acid | CNR1 | moderate |
| Enanthic Acid | CNR2 | moderate |
| Enanthic Acid | CYP2C8 | moderate |
| Enanthic Acid | ECI2 | moderate |
| Enanthic Acid | ELOVL4 | moderate |
| Enanthic Acid | FADS1 | moderate |
| Enanthic Acid | FADS2 | moderate |
| Enanthic Acid | FFAR1 | moderate |
| Enanthic Acid | FKBP1A | moderate |
| Enanthic Acid | FURIN | moderate |
| Enanthic Acid | GLTP | moderate |
| Enanthic Acid | GM2A | moderate |
| Enanthic Acid | GUCA1A | moderate |
| Enanthic Acid | HAO1 | moderate |
| Enanthic Acid | HDAC2 | moderate |
| Enanthic Acid | HDAC9 | moderate |
| Enanthic Acid | HNF4A | moderate |
| Enanthic Acid | HNF4G | moderate |
| Enanthic Acid | INS | moderate |
| Enanthic Acid | LALBA | moderate |
| Enanthic Acid | LIP3 | moderate |
| Enanthic Acid | LTB4R | moderate |
| Enanthic Acid | LTB4R2 | moderate |
| Enanthic Acid | LTF | moderate |
| Enanthic Acid | LY96 | moderate |
| Enanthic Acid | NCOA1 | moderate |
| Enanthic Acid | NR1H4 | moderate |
| Enanthic Acid | OGDH | moderate |
| Enanthic Acid | PAEP | moderate |
| Enanthic Acid | PKIA | moderate |
| Enanthic Acid | PLA2G1B | moderate |
| Enanthic Acid | PLA2G2A | moderate |
| Enanthic Acid | PLA2G2D | moderate |
| Enanthic Acid | PLA2G2E | moderate |
| Enanthic Acid | PMP2 | moderate |
| Enanthic Acid | PPARA | moderate |
| Enanthic Acid | PPARD | moderate |
| Enanthic Acid | PPARG | moderate |
| Enanthic Acid | PPA_RS05235 | moderate |
| Enanthic Acid | PPP3CA | moderate |
| Enanthic Acid | PPP3R1 | moderate |
| Enanthic Acid | PPT1 | moderate |
| Enanthic Acid | PRKACA | moderate |
| Enanthic Acid | PTGS1 | moderate |
| Enanthic Acid | PTGS2 | moderate |
| Enanthic Acid | PVR | moderate |
| Enanthic Acid | RCVRN | moderate |
| Enanthic Acid | RHO | moderate |
| Enanthic Acid | RXRA | moderate |
| Enanthic Acid | RXRB | moderate |
| Enanthic Acid | RXRG | moderate |
| Enanthic Acid | S100B | moderate |
| Enanthic Acid | SCN10A | moderate |
| Enanthic Acid | SCN11A | moderate |
| Enanthic Acid | SCN1A | moderate |
| Enanthic Acid | SCN1B | moderate |
| Enanthic Acid | SCN2A | moderate |
| Enanthic Acid | SCN2B | moderate |
| Enanthic Acid | SCN3A | moderate |
| Enanthic Acid | SCN3B | moderate |
| Enanthic Acid | SCN4A | moderate |
| Enanthic Acid | SCN4B | moderate |
| Enanthic Acid | SCN5A | moderate |
| Enanthic Acid | SCN7A | moderate |
| Enanthic Acid | SCN8A | moderate |
| Enanthic Acid | SCN9A | moderate |
| Enanthic Acid | SEC14L2 | moderate |
| Enanthic Acid | SLC8A1 | moderate |
| Enanthic Acid | SREBF1 | moderate |
| Enanthic Acid | TLR4 | moderate |
| Enanthic Acid | TM1468 | moderate |
| Enanthic Acid | TRAPPC3 | moderate |
| Enanthic Acid | TRPV1 | moderate |
| Enanthic Acid | VLDLR | moderate |
| Nonanoic Acid | ABAT | moderate |
| Nonanoic Acid | ABL1 | moderate |
| Nonanoic Acid | ACADSB | moderate |
| Nonanoic Acid | ACOX1 | moderate |
| Nonanoic Acid | ACSL3 | moderate |
| Nonanoic Acid | ACSL4 | moderate |
| Nonanoic Acid | ADH5 | moderate |
| Nonanoic Acid | ALDH5A1 | moderate |
| Nonanoic Acid | ARF1 | moderate |
| Nonanoic Acid | ARF6 | moderate |
| Nonanoic Acid | C8G | moderate |
| Nonanoic Acid | CALM1 | moderate |
| Nonanoic Acid | CNR1 | moderate |
| Nonanoic Acid | CNR2 | moderate |
| Nonanoic Acid | CYP2C8 | moderate |
| Nonanoic Acid | ECI2 | moderate |
| Nonanoic Acid | ELOVL4 | moderate |
| Nonanoic Acid | FADS1 | moderate |
| Nonanoic Acid | FADS2 | moderate |
| Nonanoic Acid | FFAR1 | moderate |
| Nonanoic Acid | FKBP1A | moderate |
| Nonanoic Acid | FURIN | moderate |
| Nonanoic Acid | GLTP | moderate |
| Nonanoic Acid | GM2A | moderate |
| Nonanoic Acid | GUCA1A | moderate |
| Nonanoic Acid | HAO1 | moderate |
| Nonanoic Acid | HDAC2 | moderate |
| Nonanoic Acid | HDAC9 | moderate |
| Nonanoic Acid | HNF4A | moderate |
| Nonanoic Acid | HNF4G | moderate |
| Nonanoic Acid | INS | moderate |
| Nonanoic Acid | LALBA | moderate |
| Nonanoic Acid | LIP3 | moderate |
| Nonanoic Acid | LTB4R | moderate |
| Nonanoic Acid | LTB4R2 | moderate |
| Nonanoic Acid | LTF | moderate |
| Nonanoic Acid | LY96 | moderate |
| Nonanoic Acid | NCOA1 | moderate |
| Nonanoic Acid | NR1H4 | moderate |
| Nonanoic Acid | OGDH | moderate |
| Nonanoic Acid | PAEP | moderate |
| Nonanoic Acid | PKIA | moderate |
| Nonanoic Acid | PLA2G1B | moderate |
| Nonanoic Acid | PLA2G2A | moderate |
| Nonanoic Acid | PLA2G2D | moderate |
| Nonanoic Acid | PLA2G2E | moderate |
| Nonanoic Acid | PMP2 | moderate |
| Nonanoic Acid | PPARA | moderate |
| Nonanoic Acid | PPARD | moderate |
| Nonanoic Acid | PPARG | moderate |
| Nonanoic Acid | PPA_RS05235 | moderate |
| Nonanoic Acid | PPP3CA | moderate |
| Nonanoic Acid | PPP3R1 | moderate |
| Nonanoic Acid | PPT1 | moderate |
| Nonanoic Acid | PRKACA | moderate |
| Nonanoic Acid | PTGS1 | moderate |
| Nonanoic Acid | PTGS2 | moderate |
| Nonanoic Acid | PVR | moderate |
| Nonanoic Acid | RCVRN | moderate |
| Nonanoic Acid | RHO | moderate |
| Nonanoic Acid | RXRA | moderate |
| Nonanoic Acid | RXRB | moderate |
| Nonanoic Acid | RXRG | moderate |
| Nonanoic Acid | S100B | moderate |
| Nonanoic Acid | SCN10A | moderate |
| Nonanoic Acid | SCN11A | moderate |
| Nonanoic Acid | SCN1A | moderate |
| Nonanoic Acid | SCN1B | moderate |
| Nonanoic Acid | SCN2A | moderate |
| Nonanoic Acid | SCN2B | moderate |
| Nonanoic Acid | SCN3A | moderate |
| Nonanoic Acid | SCN3B | moderate |
| Nonanoic Acid | SCN4A | moderate |
| Nonanoic Acid | SCN4B | moderate |
| Nonanoic Acid | SCN5A | moderate |
| Nonanoic Acid | SCN7A | moderate |
| Nonanoic Acid | SCN8A | moderate |
| Nonanoic Acid | SCN9A | moderate |
| Nonanoic Acid | SEC14L2 | moderate |
| Nonanoic Acid | SLC8A1 | moderate |
| Nonanoic Acid | SREBF1 | moderate |
| Nonanoic Acid | TLR4 | moderate |
| Nonanoic Acid | TM1468 | moderate |
| Nonanoic Acid | TRAPPC3 | moderate |
| Nonanoic Acid | TRPV1 | moderate |
| Nonanoic Acid | VLDLR | moderate |
| Niacin | HCAR2 | moderate |
| Niacin | HCAR3 | moderate |
| Niacin | NNMT | moderate |
| Niacin | QPRT | moderate |
| Azelaic Acid | AKR1D1 | moderate |
| Azelaic Acid | ALAD | moderate |
| Azelaic Acid | ALDH5A1 | moderate |
| Azelaic Acid | ASPH | moderate |
| Azelaic Acid | BBOX1 | moderate |
| Azelaic Acid | CPA1 | moderate |
| Azelaic Acid | HBA1 | moderate |
| Azelaic Acid | HBB | moderate |
| Azelaic Acid | HSD17B6 | moderate |
| Azelaic Acid | OXCT1 | moderate |
| Azelaic Acid | OXCT2 | moderate |
| Azelaic Acid | P3H1 | moderate |
| Azelaic Acid | P3H2 | moderate |
| Azelaic Acid | P3H3 | moderate |
| Azelaic Acid | P4HA1 | moderate |
| Azelaic Acid | P4HA2 | moderate |
| Azelaic Acid | PLOD1 | moderate |
| Azelaic Acid | PLOD3 | moderate |
| Azelaic Acid | SDHA | moderate |
| Azelaic Acid | SDHB | moderate |
| Azelaic Acid | SDHC | moderate |
| Azelaic Acid | SDHD | moderate |
| Azelaic Acid | SLC13A1 | moderate |
| Azelaic Acid | SLC13A2 | moderate |
| Azelaic Acid | SLC13A3 | moderate |
| Azelaic Acid | SLC25A10 | moderate |
| Azelaic Acid | SRD5A2 | moderate |
| Azelaic Acid | SUCLA2 | moderate |
| Azelaic Acid | SUCLG1 | moderate |
| Azelaic Acid | SUCLG2 | moderate |
| Azelaic Acid | SUCNR1 | moderate |
| Azelaic Acid | TMLHE | moderate |
| Azelaic Acid | TYR | moderate |
| N-Hexyl-Î’-D-Glucopyranoside | ABO | moderate |
| N-Hexyl-Î’-D-Glucopyranoside | ADRB1 | moderate |
| N-Hexyl-Î’-D-Glucopyranoside | AQP1 | moderate |
| N-Hexyl-Î’-D-Glucopyranoside | BXE_RS00860 | moderate |
| N-Hexyl-Î’-D-Glucopyranoside | CLPS | moderate |
| N-Hexyl-Î’-D-Glucopyranoside | CYC1 | moderate |
| N-Hexyl-Î’-D-Glucopyranoside | CYP2B6 | moderate |
| N-Hexyl-Î’-D-Glucopyranoside | ETFDH | moderate |
| N-Hexyl-Î’-D-Glucopyranoside | FKBP1A | moderate |
| N-Hexyl-Î’-D-Glucopyranoside | KCNJ3 | moderate |
| N-Hexyl-Î’-D-Glucopyranoside | LCT | moderate |
| N-Hexyl-Î’-D-Glucopyranoside | MIP | moderate |
| N-Hexyl-Î’-D-Glucopyranoside | MT-CYB | moderate |
| N-Hexyl-Î’-D-Glucopyranoside | PNLIP | moderate |
| N-Hexyl-Î’-D-Glucopyranoside | PPARD | moderate |
| N-Hexyl-Î’-D-Glucopyranoside | RARG | moderate |
| N-Hexyl-Î’-D-Glucopyranoside | RHO | moderate |
| N-Hexyl-Î’-D-Glucopyranoside | SDHA | moderate |
| N-Hexyl-Î’-D-Glucopyranoside | SDHB | moderate |
| N-Hexyl-Î’-D-Glucopyranoside | SDHC | moderate |
| N-Hexyl-Î’-D-Glucopyranoside | SDHD | moderate |
| N-Hexyl-Î’-D-Glucopyranoside | SERPINA1 | moderate |
| N-Hexyl-Î’-D-Glucopyranoside | TGM3 | moderate |
| N-Hexyl-Î’-D-Glucopyranoside | TTHA1133 | moderate |
| N-Hexyl-Î’-D-Glucopyranoside | TTHA1134 | moderate |
| N-Hexyl-Î’-D-Glucopyranoside | TTHA1135 | moderate |
| N-Hexyl-Î’-D-Glucopyranoside | UQCR10 | moderate |
| N-Hexyl-Î’-D-Glucopyranoside | UQCRB | moderate |
| N-Hexyl-Î’-D-Glucopyranoside | UQCRC1 | moderate |
| N-Hexyl-Î’-D-Glucopyranoside | UQCRC2 | moderate |
| N-Hexyl-Î’-D-Glucopyranoside | UQCRFS1 | moderate |
| N-Hexyl-Î’-D-Glucopyranoside | UQCRH | moderate |
| N-Hexyl-Î’-D-Glucopyranoside | UQCRQ | moderate |
| Methyl Caprylate | IGHG1 | moderate |
| Methyl Caprylate | LCN2 | moderate |
| (X{2212})-Nicotine | CHAT | moderate |
| (X{2212})-Nicotine | CHRNA10 | moderate |
| (X{2212})-Nicotine | CHRNA2 | moderate |
| (X{2212})-Nicotine | CHRNA3 | moderate |
| (X{2212})-Nicotine | CHRNA4 | moderate |
| (X{2212})-Nicotine | CHRNA5 | moderate |
| (X{2212})-Nicotine | CHRNA6 | moderate |
| (X{2212})-Nicotine | CHRNA7 | moderate |
| (X{2212})-Nicotine | CHRNA9 | moderate |
| (X{2212})-Nicotine | CHRNB2 | moderate |
| (X{2212})-Nicotine | CHRNB3 | moderate |
| (X{2212})-Nicotine | CHRNB4 | moderate |
| (X{2212})-Nicotine | CYP19A1 | moderate |
