# Supplementary material for: The mechanistic study of codonopsis pilosula on laryngeal squamous cell carcinoma based on network pharmacology and experimental validation
Source: Front Pharmacol. 2025 Apr 25;16:1542116. doi: 10.3389/fphar.2025.1542116 (PMC12061682; doi:10.3389/fphar.2025.1542116)
Supplement: Supplementary file 1 [file DataSheet1.zip › Supplementary Material/Supplementary_Table S4.docx]

**Supplementary Table S4.** Effective compounds and targets of Codonopsis pilosula.

| **Chemical compound** | **Target genes** |
| --- | --- |
| PYRIDINE | TEK |
| PYRIDINE | GRM5 |
| PYRIDINE | BTK |
| PYRIDINE | SIRT3 |
| PYRIDINE | TTK |
| PYRIDINE | DSPP |
| Pyridoxal | GLUD1 |
| Azelaic Acid | BAX |
| Azelaic Acid | SRD5A3 |
| Azelaic Acid | TYR |
| Azelaic Acid | SRD5A2 |
| Azelaic Acid | AKR1D1 |
| Azelaic Acid | SRD5A1 |
| Atractylenolide III | CASP3 |
| Atractylenolide III | CASP9 |
| Atractylenolide III | BAX |
| Atractylenolide III | BCL2 |
| Nicotine | CYP19A1 |
| Nicotine | PTHLH |
| Nicotine | PNPLA2 |
| Nicotine | MCL1 |
| Nicotine | CHRNE |
| Nicotine | CHRFAM7A |
| Nicotine | BDNF |
| Nicotine | CHRNA2 |
| Nicotine | CDH2 |
| Nicotine | TNFSF9 |
| Nicotine | ERBB4 |
| Nicotine | CDH1 |
| Nicotine | CHRNA5 |
| Nicotine | CHRND |
| Nicotine | CRHR2 |
| Nicotine | DRD3 |
| Nicotine | CYP3A4 |
| Nicotine | CHAT |
| Nicotine | CHRNB4 |
| Nicotine | HDAC6 |
| Nicotine | CHRNB1 |
| Nicotine | TET2 |
| Nicotine | NR1H3 |
| Nicotine | PAK1 |
| Nicotine | ESR2 |
| Nicotine | GHRL |
| Nicotine | AKT1 |
| Nicotine | CHRNA6 |
| Nicotine | VIM |
| Nicotine | NRG3 |
| Nicotine | CHRNA3 |
| Nicotine | MMP9 |
| Nicotine | CHRNB2 |
| Nicotine | NR3C1 |
| Nicotine | PIK3CB |
| Nicotine | MGMT |
| Nicotine | CARTPT |
| Nicotine | CHRNG |
| Nicotine | CHRNA9 |
| Nicotine | EGR1 |
| Nicotine | SLC1A3 |
| Nicotine | CHGA |
| Nicotine | MMP2 |
| Nicotine | SHC1 |
| Nicotine | CRP |
| Nicotine | CHRNA7 |
| Nicotine | CRHR1 |
| Nicotine | HINT1 |
| Nicotine | CREB1 |
| Nicotine | FOS |
| Nicotine | NGF |
| Nicotine | ABCB1 |
| Nicotine | CRHBP |
| Nicotine | NFKB1 |
| Nicotine | UGT2B10 |
| Nicotine | CD24 |
| Nicotine | CHRNA10 |
| Nicotine | VCAM1 |
| Nicotine | PPP1R12A |
| Nicotine | CHRNB3 |
| Nicotine | GAD1 |
| Nicotine | CHRNA4 |
| Nicotine | SCARB1 |
| Nicotine | MAPK1 |
| Nicotine | FLG |
| Nicotine | ABCA1 |
| Nicotine | GAST |
| Nicotine | STAT3 |
| Nicotine | CHRNA1 |
| Nicotine | MAPK3 |
| Nicotine | ABCG1 |
| ERGOTAMINE | DRD5 |
| ERGOTAMINE | ADRA2C |
| ERGOTAMINE | HTR2A |
| ERGOTAMINE | ADRA2A |
| ERGOTAMINE | HTR1F |
| ERGOTAMINE | HTR2C |
| ERGOTAMINE | HTR1A |
| ERGOTAMINE | HTR1D |
| ERGOTAMINE | ADRA1A |
| ERGOTAMINE | ADRA1B |
| ERGOTAMINE | DRD2 |
| ERGOTAMINE | DRD1 |
| ERGOTAMINE | HTR1B |
| ERGOTAMINE | ADRA1D |
| ERGOTAMINE | HTR2B |
| 1-Octanol | TRPA1 |
| D-Arabino-Hex-2-Ulo-Pyranose | FHIT |
| 1,3-Dicyclohexylurea | EPHX2 |
| Palmitic Acid | APOM |
| Palmitic Acid | LALBA |
| Palmitic Acid | IL1A |
| Palmitic Acid | RHO |
| Palmitic Acid | PPT1 |
| Palmitic Acid | IDE |
| Palmitic Acid | PAEP |
| Palmitic Acid | ABHD15 |
| Palmitic Acid | CRTC2 |
| Palmitic Acid | TRAPPC3 |
| Palmitic Acid | YWHAZ |
| Palmitic Acid | HNF4G |
| Palmitic Acid | IL10 |
| Palmitic Acid | TNF |
| Palmitic Acid | PPARA |
| Palmitic Acid | GUCA2A |
| Palmitic Acid | CYP2C8 |
| Palmitic Acid | TLR2 |
| Palmitic Acid | PMP2 |
| Palmitic Acid | PTEN |
| Palmitic Acid | BCL2 |
| Borneol | TRPM8 |
| Sulforaphane | TXNRD1 |
| Sulforaphane | MIR135B |
| Sulforaphane | SLC2A3 |
| Sulforaphane | RASAL2 |
| Sulforaphane | CCL2 |
| Sulforaphane | GCLC |
| Sulforaphane | SERTAD1 |
| Sulforaphane | STAT5B |
| Sulforaphane | AQP3 |
| Sulforaphane | MERTK |
| Sulforaphane | ICAM1 |
| Sulforaphane | HDAC9 |
| Sulforaphane | KEAP1 |
| Sulforaphane | CTNNB1 |
| Sulforaphane | PDLIM1 |
| Sulforaphane | NFKB1 |
| Sulforaphane | VCAM1 |
| Sulforaphane | STAT5A |
| Sulforaphane | LIPE |
| Sulforaphane | MIR140 |
| Sulforaphane | AGER |
| Sulforaphane | ALDH1A1 |
| Sulforaphane | TLR4 |
| Sulforaphane | AHR |
| Sulforaphane | NFE2L2 |
| Tectoridin | PPARA |
| Emodin | CASP3 |
| Emodin | KDR |
| Emodin | ABCB11 |
| Emodin | EGF |
| Emodin | NAT10 |
| Emodin | AQP1 |
| Emodin | SLPI |
| Emodin | PTP4A3 |
| Emodin | CYP1A1 |
| Emodin | CTNNB1 |
| Emodin | NFKB1 |
| Emodin | ATRNL1 |
| Emodin | MAOB |
| Emodin | TLR4 |
| Emodin | ATF4 |
| Emodin | PPARG |
| Emodin | BTK |
| Emodin | HSPA5 |
| Emodin | TNFSF10 |
| Emodin | NFATC1 |
| Emodin | PRKAA2 |
| Emodin | SARS |
| Emodin | RUNX2 |
| Emodin | EGR1 |
| Emodin | PRKAA1 |
| Emodin | FLT4 |
| Emodin | MYC |
| Emodin | ALPL |
| Emodin | BCL2 |
| Emodin | CSNK2A2 |
| Emodin | ASRGL1 |
| Emodin | CCL27 |
| Emodin | FXR1 |
| Emodin | ROS1 |
| Emodin | MIR371A |
| Emodin | HIF1A |
| Emodin | AR |
| Emodin | ESR1 |
| Emodin | NOS2 |
| Emodin | CLDN5 |
| Emodin | MTA3 |
| Emodin | SLC2A4 |
| Emodin | MTOR |
| Emodin | SLC2A1 |
| Emodin | TP53 |
| Emodin | IL1B |
| Emodin | CDH1 |
| Emodin | PPP1R14A |
| Emodin | TWIST1 |
| Emodin | KIT |
| Emodin | SIRT1 |
| Emodin | AKT1 |
| Emodin | BGLAP |
| Emodin | MMP1 |
| Emodin | VIM |
| Emodin | SMAD3 |
| Emodin | JAK1 |
| Emodin | PDLIM3 |
| Emodin | CYP1A2 |
| Emodin | FLT1 |
| Emodin | MAPK1 |
| Emodin | MMP9 |
| Emodin | FOXD3 |
| Emodin | ATHS |
| Emodin | TGFB2 |
| Emodin | SRC |
| Emodin | TNFSF11 |
| Emodin | ALPP |
| Emodin | HSD11B1 |
| Emodin | PRKCD |
| Emodin | BAX |
| Emodin | JAK2 |
| Emodin | HSP90AA1 |
| Emodin | STAT3 |
| Emodin | TNF |
| Emodin | TGFB1 |
| Emodin | EDN1 |
| Emodin | CSF2 |
| Emodin | FOS |
| Emodin | MIR145 |
| Emodin | MAPK3 |
| Emodin | ABCB1 |
| Emodin | FASLG |
| Emodin | AQP5 |
| Emodin | PGAM1 |
| Emodin | OCLN |
| Emodin | DDIT3 |
| Octanoic Acid | FABP1 |
| Octanoic Acid | ISYNA1 |
| D-Methionine | METAP2 |
| ETHYL PALMITATE | AGT |
| Lauric Acid | LY96 |
| Lauric Acid | HNF4A |
| Lauric Acid | PLA2G2D |
| Lauric Acid | ADH5 |
| Lauric Acid | GCG |
| Lauric Acid | VLDLR |
| Lauric Acid | PLA2G2A |
| Lauric Acid | C8G |
| Lauric Acid | TLR4 |
| Lauric Acid | GM2A |
| Lauric Acid | PPARA |
| Lauric Acid | LTF |
| Lauric Acid | GLTP |
| Cyclanoline | ACHE |
| Alpha-Spinasterol | NR1H3 |
| Alpha-Spinasterol | NR1H2 |
| Alpha-Spinasterol | TRPV1 |
| Vanillic Acid | PPARG |
| Vanillic Acid | TYR |
| Vanillic Acid | CEBPA |
| Vanillic Acid | CA3 |
| Vanillic Acid | HIF1A |
| Vanillic Acid | NOS3 |
| Vanillic Acid | TYRP1 |
| Glycine | ALAS1 |
| Glycine | GLRB |
| Glycine | GLYAT |
| Glycine | AGXT |
| Glycine | GARS |
| Glycine | GCAT |
| Glycine | GSS |
| Glycine | GPR18 |
| Glycine | AGXT2 |
| Glycine | ALAS2 |
| Glycine | GNMT |
| Glycine | GRIN2C |
| Glycine | GLYATL1 |
| Glycine | GATM |
| Glycine | GLRA1 |
| Glycine | GLRA3 |
| Glycine | GRIN2A |
| Glycine | SHMT1 |
| Glycine | GLRA2 |
| Glycine | PIPOX |
| Glycine | GLYATL2 |
| Glycine | BAAT |
| Glycine | SHMT2 |
| Glycine | GRIN3B |
| Glycycoumarin | MAPK8 |
| Glycycoumarin | CASP3 |
| Glycycoumarin | UGT1A9 |
| Glycycoumarin | NFE2L2 |
| Vitamin D3 | CYP19A1 |
| Vitamin D3 | TXN |
| Vitamin D3 | IGF1 |
| Vitamin D3 | CASR |
| Vitamin D3 | TGFA |
| Vitamin D3 | TNFRSF11B |
| Vitamin D3 | S100G |
| Vitamin D3 | MMP2 |
| Vitamin D3 | FOXP3 |
| Vitamin D3 | TLR2 |
| Vitamin D3 | DYSF |
| Vitamin D3 | AR |
| Vitamin D3 | TG |
| Vitamin D3 | DHCR7 |
| Vitamin D3 | PTH |
| Vitamin D3 | RELA |
| Vitamin D3 | CAMP |
| Vitamin D3 | IL10 |
| Vitamin D3 | VDR |
| Vitamin D3 | DBP |
| Vitamin D3 | IL4 |
| Vitamin D3 | TNFSF11 |
| Vitamin D3 | IL6 |
| Vitamin D3 | CYP24A1 |
| Vitamin D3 | CD86 |
| Vitamin D3 | RXRA |
| Vitamin D3 | CCR6 |
| Vitamin D3 | NFKB1 |
| Vitamin D3 | TLR4 |
| Vitamin D3 | CD55 |
| Vitamin D3 | CXCL8 |
| Vitamin D3 | REN |
| Vitamin D3 | CAMKK2 |
| Vitamin D3 | PRL |
| Vitamin D3 | CYP27B1 |
| Vitamin D3 | NFKBIA |
| Vitamin D3 | SMO |
| Vitamin D3 | IGFBP3 |
| Vitamin D3 | GLI1 |
| Vitamin D3 | GJA1 |
| Vitamin D3 | AKT1 |
| Vitamin D3 | CD14 |
| Vitamin D3 | CD40 |
| Vitamin D3 | PTGS2 |
| Vitamin D3 | CYP11A1 |
| Vitamin D3 | MAPK1 |
| Vitamin D3 | MMP9 |
| Vitamin D3 | CCR7 |
| Vitamin D3 | CSRP1 |
| Vitamin D3 | CD80 |
| Vitamin D3 | CD83 |
| Vitamin D3 | MAPK3 |
| Stearic Acid | PLA2G2D |
| Stearic Acid | CPT1B |
| Stearic Acid | HDAC6 |
| Stearic Acid | CDK1 |
| Stearic Acid | PTPN1 |
| Stearic Acid | PPARA |
| Stearic Acid | ABCB1 |
| Stearic Acid | ENPP7 |
| Stearic Acid | MAPK1 |
| Tetrandrine | CASP3 |
| Tetrandrine | TSC2 |
| Tetrandrine | CDKN1A |
| Tetrandrine | BECN1 |
| Tetrandrine | CTNNB1 |
| Tetrandrine | NFKB1 |
| Tetrandrine | MCL1 |
| Tetrandrine | NFATC1 |
| Tetrandrine | KHDRBS1 |
| Tetrandrine | SMAD7 |
| Tetrandrine | RB1 |
| Tetrandrine | SQSTM1 |
| Tetrandrine | MYC |
| Tetrandrine | GTF2H1 |
| Tetrandrine | BCL2 |
| Tetrandrine | MAP1LC3A |
| Tetrandrine | NUP62 |
| Tetrandrine | MTOR |
| Tetrandrine | TP53 |
| Tetrandrine | PRKACA |
| Tetrandrine | CCND1 |
| Tetrandrine | MTA1 |
| Tetrandrine | CDKN1B |
| Tetrandrine | AKT1 |
| Tetrandrine | CBL |
| Tetrandrine | MAPK1 |
| Tetrandrine | SRC |
| Tetrandrine | DCTN4 |
| Tetrandrine | BAX |
| Tetrandrine | CD44 |
| Tetrandrine | SYK |
| Tetrandrine | NOTCH1 |
| Tetrandrine | PIK3C3 |
| Tetrandrine | SKP2 |
| Tetrandrine | ABCB1 |
| Tetrandrine | AHR |
| BETA-SITOSTEROL | CASP3 |
| BETA-SITOSTEROL | PRKCA |
| BETA-SITOSTEROL | CASP9 |
| BETA-SITOSTEROL | MAP2 |
| BETA-SITOSTEROL | PRKAA2 |
| BETA-SITOSTEROL | BCL2 |
| BETA-SITOSTEROL | CASP8 |
| BETA-SITOSTEROL | BAX |
| BETA-SITOSTEROL | TGFB1 |
| BETA-SITOSTEROL | EDN1 |
| Choline | PHOSPHO1 |
| Choline | MAPK14 |
| Choline | BCHE |
| Choline | CHRFAM7A |
| Choline | SIGMAR1 |
| Choline | CHRNA4 |
| Choline | PLD1 |
| Choline | ACHE |
| Choline | PCYT1B |
| Choline | HMOX1 |
| Choline | CHRNA7 |
| Choline | PEMT |
| Choline | PLD2 |
| Choline | TNF |
| Choline | VEGFC |
| Choline | PCYT1A |
| QSPL 173 | NR1H2 |
| (-)-Borneol | VEGFA |
| (-)-Borneol | TJP1 |
| (-)-Borneol | TRPM8 |
| (-)-Borneol | MMP9 |
| (-)-Borneol | UGT1A6 |
| Syringin | TRAF6 |
| Syringin | HSPA4 |
| Isorhamnetin | MAPK8 |
| Isorhamnetin | CASP3 |
| Isorhamnetin | OLR1 |
| Isorhamnetin | SLCO1B1 |
| Isorhamnetin | MAP2K1 |
| Isorhamnetin | CCNB1 |
| Isorhamnetin | MAPK14 |
| Isorhamnetin | WEE1 |
| Isorhamnetin | SLC47A1 |
| Isorhamnetin | NCF1 |
| Isorhamnetin | CASP8 |
| Isorhamnetin | CAMK2G |
| Isorhamnetin | TNF |
| Isorhamnetin | AKT1 |
| Isorhamnetin | PARP1 |
| Isorhamnetin | MAP2K7 |
| Isorhamnetin | PPARG |
| Isorhamnetin | EGFR |
| Isorhamnetin | PTGS2 |
| Isorhamnetin | CSK |
| Isorhamnetin | CRMP1 |
| Isorhamnetin | NOS2 |
| Isorhamnetin | XDH |
| Nonnanoic acid | ISYNA1 |
| Nonnanoic acid | OR51E1 |
| Beta-D-Ribofuranose | RYR1 |
| Methyl palmitate | IL10 |
| Methyl palmitate | PTGS2 |
| Methyl palmitate | TNF |
| Methyl palmitate | IL6 |
| DL-Glyceraldehyde | AKR1B1 |
| (-)-Alpha-Pinene | NFKB1 |
| Tetradecanoic Acid | DGKD |
| Tetradecanoic Acid | SIGMAR1 |
| Ferulic Acid | CYP2C9 |
| Ferulic Acid | ENO2 |
| Ferulic Acid | PRKAA1 |
| Ferulic Acid | MAP2K1 |
| Ferulic Acid | APP |
| Ferulic Acid | PVALB |
| Ferulic Acid | CTNNB1 |
| Ferulic Acid | BCHE |
| Ferulic Acid | PEA15 |
| Ferulic Acid | MAP2K2 |
| Ferulic Acid | PRDX2 |
| Ferulic Acid | HPCA |
| Ferulic Acid | CXCL8 |
| Ferulic Acid | MAOA |
| Ferulic Acid | ACHE |
| Ferulic Acid | NFE2L2 |
| Phenol | ALB |
| Phenol | ACE |
| Phenol | MB |
| Phenol | AGTR2 |
| Phenol | STAT3 |
| Azelastine | HRH2 |
| Azelastine | LTC4S |
| Azelastine | PLA2G1B |
| Azelastine | HRH1 |
| 7-Ketocholesterol | MAP1LC3B |
| 7-Ketocholesterol | CASP9 |
| 7-Ketocholesterol | BCL2 |
| 7-Ketocholesterol | TP53 |
| 7-Ketocholesterol | BID |
| 7-Ketocholesterol | SMO |
| 7-Ketocholesterol | CASP8 |
| 7-Ketocholesterol | PARP1 |
| 7-Ketocholesterol | AKT1 |
| 7-Ketocholesterol | BAX |
| Allopregnanolone | GABRQ |
| Allopregnanolone | GABRA3 |
| Allopregnanolone | GABRA6 |
| Allopregnanolone | NR1I2 |
| Allopregnanolone | GABRB2 |
| Allopregnanolone | GABRG1 |
| Allopregnanolone | GABRG3 |
| Allopregnanolone | GABRD |
| Allopregnanolone | GABRA2 |
| Allopregnanolone | GABRA4 |
| Allopregnanolone | GABRA1 |
| Allopregnanolone | GABRB3 |
| Allopregnanolone | GABRB1 |
| Allopregnanolone | GABRG2 |
| Allopregnanolone | GABRP |
| Allopregnanolone | GABRA5 |
| Allopregnanolone | GABRE |
| Alpha-D-Mannose | TPO |
| Alpha-D-Mannose | ITIH4 |
| Alpha-D-Mannose | POMK |
| Alpha-D-Mannose | PPARG |
| Alpha-D-Mannose | CD209 |
| Nicotinic Acid | DGAT2 |
| Nicotinic Acid | HCAR2 |
| Nicotinic Acid | NNMT |
| Nicotinic Acid | LIPG |
| Nicotinic Acid | SIRT1 |
| Nicotinic Acid | LPL |
| Nicotinic Acid | HP |
| Nicotinic Acid | HIF1A |
| Nicotinic Acid | APOB |
| Nicotinic Acid | MAGEC3 |
| Nicotinic Acid | MAPK14 |
| Nicotinic Acid | QPRT |
| Nicotinic Acid | ADIPOQ |
| Nicotinic Acid | NOS3 |
| Nicotinic Acid | CETP |
| Nicotinic Acid | TRPV1 |
| Nicotinic Acid | HCAR3 |
| Stigmasterol | ABCB11 |
| Stigmasterol | APP |
| Stigmasterol | TLR4 |
| Stigmasterol | AKT1 |
| Stigmasterol | ABCC2 |
| Stigmasterol | BACE1 |
| Stigmasterol | NR1H3 |
| Stigmasterol | NR1H2 |
| Stigmasterol | NR1H4 |
| Stigmasterol | ABCG5 |
| Stigmasterol | ABCG8 |
| (+)-L-Tartaric Acid | UGCG |
| 1-Butanol | PLD1 |
| Fangchinoline | ACHE |
| Linoleic Acid | CCL2 |
| Linoleic Acid | NPPB |
| Linoleic Acid | JUN |
| Linoleic Acid | NPPA |
| Linoleic Acid | POMC |
| Linoleic Acid | ICAM1 |
| Linoleic Acid | FOS |
| Linoleic Acid | LCN2 |
| Linoleic Acid | NFKB1 |
| Linoleic Acid | SCD |
| Linoleic Acid | BCL2 |
| Linoleic Acid | PLA2G7 |
| Linoleic Acid | PPARA |
| Linoleic Acid | NFATC4 |
| Lupeol | LAMP1 |
| Lupeol | CASP3 |
| Lupeol | CATSPER1 |
| Lupeol | AKR1B1 |
| Lupeol | TIPARP |
| Lupeol | PLK1 |
| Lupeol | IFNG |
| Lupeol | VEGFA |
| Lupeol | CDC25C |
| Lupeol | CDK1 |
| Lupeol | PTGS2 |
| Lupeol | MMP9 |
| Lupeol | SGK1 |
| Lupeol | BCL2 |
| Lupeol | STAT3 |
| Lupeol | PRF1 |
| Lupeol | KLK3 |
| Lupeol | EGFR |
| Lupeol | IL6 |
| Lupeol | AR |
| Lupeol | ESR1 |
| Lupeol | SFN |
| Methyl (4aR,7aS)-7-(Hydroxymethyl)-1-[3,4,5-Trihydroxy-6-(Hydroxymethyl)Oxan-2-Yl]Oxy-1,4a,5,7a-Tetrahydrocyclopenta[C]Pyran-4-Carboxylate | AKT1 |
| Methyl (4aR,7aS)-7-(Hydroxymethyl)-1-[3,4,5-Trihydroxy-6-(Hydroxymethyl)Oxan-2-Yl]Oxy-1,4a,5,7a-Tetrahydrocyclopenta[C]Pyran-4-Carboxylate | CTNNB1 |
| Methyl (4aR,7aS)-7-(Hydroxymethyl)-1-[3,4,5-Trihydroxy-6-(Hydroxymethyl)Oxan-2-Yl]Oxy-1,4a,5,7a-Tetrahydrocyclopenta[C]Pyran-4-Carboxylate | GLP1R |
| Methyl (4aR,7aS)-7-(Hydroxymethyl)-1-[3,4,5-Trihydroxy-6-(Hydroxymethyl)Oxan-2-Yl]Oxy-1,4a,5,7a-Tetrahydrocyclopenta[C]Pyran-4-Carboxylate | NR1H4 |
| Methyl (4aR,7aS)-7-(Hydroxymethyl)-1-[3,4,5-Trihydroxy-6-(Hydroxymethyl)Oxan-2-Yl]Oxy-1,4a,5,7a-Tetrahydrocyclopenta[C]Pyran-4-Carboxylate | WNT2 |
| Methyl (4aR,7aS)-7-(Hydroxymethyl)-1-[3,4,5-Trihydroxy-6-(Hydroxymethyl)Oxan-2-Yl]Oxy-1,4a,5,7a-Tetrahydrocyclopenta[C]Pyran-4-Carboxylate | GSK3B |
| Methyl (4aR,7aS)-7-(Hydroxymethyl)-1-[3,4,5-Trihydroxy-6-(Hydroxymethyl)Oxan-2-Yl]Oxy-1,4a,5,7a-Tetrahydrocyclopenta[C]Pyran-4-Carboxylate | TCF7L2 |
| Capsaicin | GAA |
| Capsaicin | TACR1 |
| Capsaicin | TGFA |
| Capsaicin | GFAP |
| Capsaicin | TNFRSF11B |
| Capsaicin | NRP1 |
| Capsaicin | F2RL1 |
| Capsaicin | PRKAA1 |
| Capsaicin | CYCS |
| Capsaicin | HSD3B2 |
| Capsaicin | GAP43 |
| Capsaicin | EPOR |
| Capsaicin | CAT |
| Capsaicin | PGP |
| Capsaicin | AR |
| Capsaicin | UCP1 |
| Capsaicin | TACR3 |
| Capsaicin | RAC1 |
| Capsaicin | HMOX1 |
| Capsaicin | ALOX5 |
| Capsaicin | ENOX2 |
| Capsaicin | TAC1 |
| Capsaicin | SPI1 |
| Capsaicin | RELA |
| Capsaicin | HSPA4 |
| Capsaicin | POMC |
| Capsaicin | NOS1 |
| Capsaicin | CREB1 |
| Capsaicin | MIR206 |
| Capsaicin | FOS |
| Capsaicin | CYP3A4 |
| Capsaicin | ABCB1 |
| Capsaicin | NFE2L2 |
| Capsaicin | CASP3 |
| Capsaicin | CALCA |
| Capsaicin | GLUL |
| Capsaicin | CTNNB1 |
| Capsaicin | SOD1 |
| Capsaicin | VEGFA |
| Capsaicin | PHB2 |
| Capsaicin | BCL2 |
| Capsaicin | NFKBIA |
| Capsaicin | TRPV1 |
| Capsaicin | TRPV2 |
| Capsaicin | SIRT1 |
| Capsaicin | GATA1 |
| Capsaicin | AKT1 |
| Capsaicin | TRPA1 |
| Capsaicin | MMP1 |
| Capsaicin | PTGS2 |
| Capsaicin | CYP1A2 |
| Capsaicin | MAPK1 |
| Capsaicin | HBB |
| Capsaicin | GYPA |
| Capsaicin | JUNB |
| Capsaicin | CDK8 |
| Capsaicin | KLK3 |
| Capsaicin | CTRC |
| L-Tryptophan | IDO1 |
| L-Tryptophan | WARS2 |
| L-Tryptophan | WARS |
| HEPTADECANE | NFKB1 |
| Glycitein | APP |
| Glycitein | MMP13 |
| Glycitein | MMP8 |
| Noscapine | SIGMAR1 |
| Noscapine | HIF1A |
| Noscapine | AHR |
| Tectorigenin | NPEPPS |
| Tectorigenin | IL1B |
| Tectorigenin | PTGER3 |
| Tectorigenin | IGF1R |
| Tectorigenin | AKR1B1 |
| Tectorigenin | FCER1A |
| Tectorigenin | SPDEF |
| Tectorigenin | PPARG |
| Tectorigenin | IRS1 |
| Tectorigenin | PTGS2 |
| Tectorigenin | TIMP3 |
| Tectorigenin | TERT |
| Tectorigenin | NOS2 |
