# Supplementary material for: The mechanistic study of codonopsis pilosula on laryngeal squamous cell carcinoma based on network pharmacology and experimental validation
Source: Front Pharmacol. 2025 Apr 25;16:1542116. doi: 10.3389/fphar.2025.1542116 (PMC12061682; doi:10.3389/fphar.2025.1542116)
Supplement: Supplementary file 1 [file DataSheet1.zip › Supplementary Material/Supplementary_Table S5.docx]

**Supplementary Table S5.** 179 codonopsis pilosula targets screened.

| **Number** | **Target genes** | **Number** | **Target genes** |
| --- | --- | --- | --- |
| 1 | PTGS2 | 91 | PPA_RS05235 |
| 2 | RXRA | 92 | PPP3CA |
| 3 | PTGS1 | 93 | PPP3R1 |
| 4 | AR | 94 | PVR |
| 5 | SCN5A | 95 | RCVRN |
| 6 | PPARG | 96 | S100B |
| 7 | NCOA2 | 97 | SEC14L2 |
| 8 | GABRA1 | 98 | SLC8A1 |
| 9 | HSP90AA1 | 99 | TM1468 |
| 10 | AKT1 | 100 | ABCB1 |
| 11 | PPARA | 101 | HTR2A |
| 12 | PGR | 102 | CHRM2 |
| 13 | NCOA1 | 103 | SLC6A2 |
| 14 | ESR1 | 104 | ADRA2A |
| 15 | CASP3 | 105 | AKR1B1 |
| 16 | BCL2 | 106 | MAOB |
| 17 | TLR4 | 107 | F7 |
| 18 | ADRB2 | 108 | GSK3B |
| 19 | TRPV1 | 109 | CDK2 |
| 20 | NFKB1 | 110 | CALM3 |
| 21 | CHRM1 | 111 | OPRM1 |
| 22 | NOS2 | 112 | RELA |
| 23 | ACHE | 113 | IL10 |
| 24 | TNF | 114 | NFKBIA |
| 25 | CHRM3 | 115 | APP |
| 26 | NOS3 | 116 | MMP1 |
| 27 | PDE3A | 117 | HMOX1 |
| 28 | ADRA1D | 118 | ICAM1 |
| 29 | ESR2 | 119 | MCL1 |
| 30 | PKIA | 120 | TYR |
| 31 | MMP9 | 121 | CYP19A1 |
| 32 | MAPK1 | 122 | ABAT |
| 33 | BAX | 123 | ACADSB |
| 34 | NR1H4 | 124 | ACOX1 |
| 35 | PLA2G2D | 125 | CNR1 |
| 36 | RHO | 126 | CNR2 |
| 37 | NR3C2 | 127 | HAO1 |
| 38 | SERPIND1 | 128 | HDAC2 |
| 39 | ADRB1 | 129 | LTB4R |
| 40 | ADRA1B | 130 | LTB4R2 |
| 41 | SLC6A3 | 131 | OGDH |
| 42 | MAPK14 | 132 | RXRB |
| 43 | PRSS1 | 133 | RXRG |
| 44 | DPP4 | 134 | SCN10A |
| 45 | INS | 135 | SCN11A |
| 46 | ADH5 | 136 | SCN1A |
| 47 | C8G | 137 | SCN1B |
| 48 | CYP2C8 | 138 | SCN2A |
| 49 | FKBP1A | 139 | SCN2B |
| 50 | GLTP | 140 | SCN3A |
| 51 | GM2A | 141 | SCN3B |
| 52 | HNF4A | 142 | SCN4A |
| 53 | HNF4G | 143 | SCN4B |
| 54 | LALBA | 144 | SCN7A |
| 55 | LTF | 145 | SCN8A |
| 56 | LY96 | 146 | SCN9A |
| 57 | PAEP | 147 | SREBF1 |
| 58 | PLA2G1B | 148 | SIGMAR1 |
| 59 | PLA2G2A | 149 | STAT3 |
| 60 | PMP2 | 150 | NFE2L2 |
| 61 | PPARD | 151 | LTA4H |
| 62 | PPT1 | 152 | TOP2B |
| 63 | PRKACA | 153 | CHEK1 |
| 64 | TRAPPC3 | 154 | CCNA2 |
| 65 | VLDLR | 155 | IL1B |
| 66 | CTNNB1 | 156 | CHRM5 |
| 67 | SLC6A4 | 157 | GRIA2 |
| 68 | DRD1 | 158 | EGFR |
| 69 | CASP9 | 159 | VEGFC |
| 70 | IL6 | 160 | CCND1 |
| 71 | TP53 | 161 | CDKN1A |
| 72 | FOS | 162 | MMP2 |
| 73 | HIF1A | 163 | RB1 |
| 74 | ABL1 | 164 | JUN |
| 75 | ACSL3 | 165 | CCNB1 |
| 76 | ACSL4 | 166 | IFNG |
| 77 | ALDH5A1 | 167 | IL4 |
| 78 | ARF1 | 168 | SLC2A4 |
| 79 | ARF6 | 169 | CDK1 |
| 80 | CALM1 | 170 | CHRNA4 |
| 81 | ECI2 | 171 | CHRNA7 |
| 82 | ELOVL4 | 172 | NR1H3 |
| 83 | FADS1 | 173 | MAPK3 |
| 84 | FADS2 | 174 | AHR |
| 85 | FFAR1 | 175 | PRKAA1 |
| 86 | FURIN | 176 | SIRT1 |
| 87 | GUCA1A | 177 | NR1H2 |
| 88 | HDAC9 | 178 | CASP8 |
| 89 | LIP3 | 179 | VEGFA |
| 90 | PLA2G2E |  |  |
