# Supplementary figures and images for: The mechanistic study of codonopsis pilosula on laryngeal squamous cell carcinoma based on network pharmacology and experimental validation
Source: Front Pharmacol. 2025 Apr 25;16:1542116. doi: 10.3389/fphar.2025.1542116 (PMC12061682; doi:10.3389/fphar.2025.1542116)

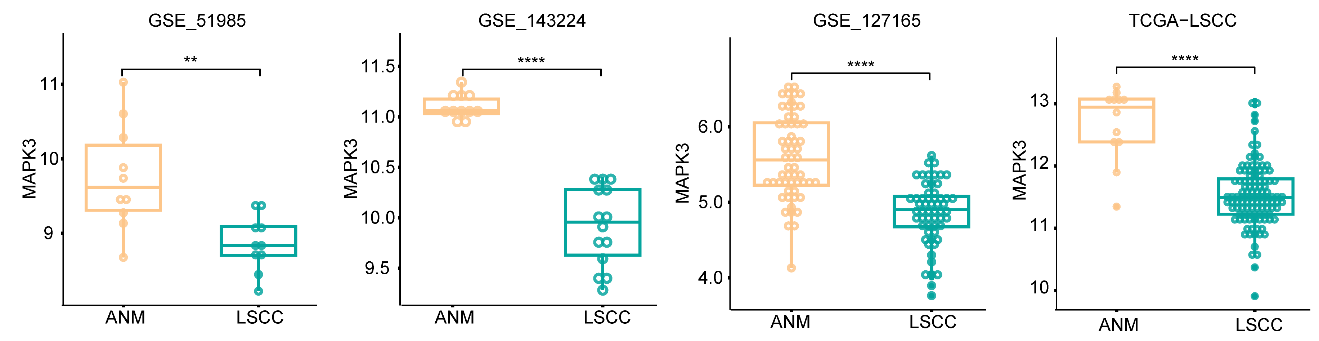
**Supplementary Figure S1.** The expression of MAPK3 in the GEO database (GSE_51985, GSE_143224, GSE_127165) and TCGA database

Supplement: Supplementary file 1 [file DataSheet1.zip › Supplementary Material/Supplementary_Figure S1.docx]

**Supplementary Figure S6.** Knockdown efficiency of siMAPK3 in LSCC cells detected by qPCR
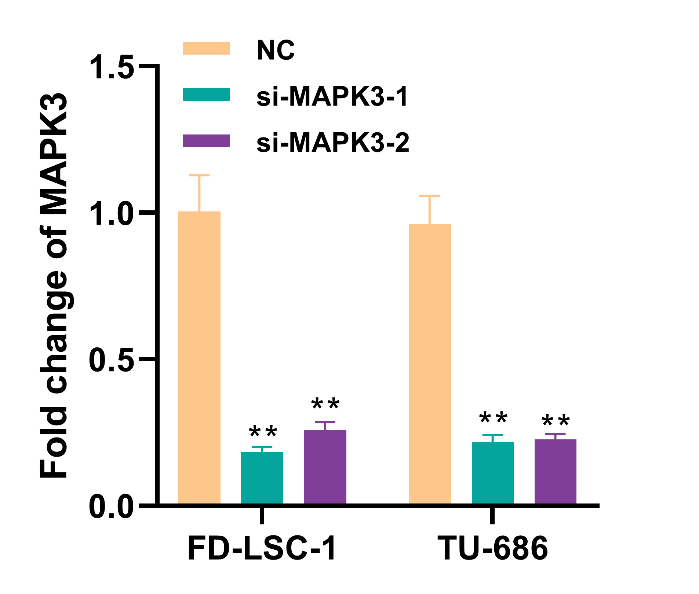

Supplement: Supplementary file 1 [file DataSheet1.zip › Supplementary Material/Supplementary_Figure S6.docx]
